# A selective agonist of the prostacyclin receptor alleviates microglial and astroglial neuroinflammatory responses through P38 and NLRP3

**DOI:** 10.3389/fimmu.2026.1664119

**Published:** 2026-03-25

**Authors:** Hyun-ju Lee, Sora Kang, Yoo Joo Jeong, Tae-Mi Jung, Jeong-Woo Hwang, Ji-Yeong Jang, Chan-Hu Gu, Seokjun Oh, Jeong-Heon Song, Minho Moon, Hyang-Sook Hoe

**Affiliations:** 1 Department of Neural Development and Disease, Korea Brain Research Institute (KBRI), Daegu, Republic of Korea; 2 AI-based Neurodevelopmental Diseases Digital Therapeutics Group, Korea Brain Research Institute (KBRI), Daegu, Republic of Korea; 3 Department of Brain Sciences, Daegu Gyeongbuk Institute of Science & Technology, Daegu, Republic of Korea; 4 Research Institute for Dementia Science, Konyang University, Daejeon, Republic of Korea

**Keywords:** IP receptor, LPS, neuroinflammation, NLRP3, P38, selexipag

## Abstract

**Introduction:**

The selective prostacyclin (IP) receptor agonist selexipag is FDA-approved for the treatment of pulmonary arterial hypertension. Selexipag also has anti-inflammatory effects in peripheral tissues, but the ability of selexipag to modulate central neuroinflammation has not been comprehensively examined. Therefore, this study investigated the effect of selexipag on LPS-mediated neuroinflammatory responses *in vitro* and *in vivo*.

**Methods:**

To examine the effects of selexipag on LPS-mediated proinflammatory responses, BV2 or primary microglial cells were treated with vehicle (1% DMSO) or selexipag in dose (0.5, 1.0, or 5.0 μM) and time (3, 6, or 24 h)-dependent manner. For *in vivo* experiments, C57BL/6N mice were injected daily for 7 days with vehicle (1% DMSO) or selexipag (1 mg/kg, i.p.). On the 7th day, the mice were administered PBS or LPS (10 mg/kg, i.p.) and sacrificed 8 h later. Neuroinflammation-associated gene and protein expression were analyzed *in vitro* and *in vivo* by real-time PCR, ELISA, immunofluorescence staining, and/or western blotting.

**Results and discussion:**

We investigated the effect of IP receptor agonist selexipag on LPS-mediated neuroinflammatory responses *in vitro* and *in vivo*. Here, we found that selexipag treatment significantly reduced LPS-induced proinflammatory mediator COX-2, IL-1β, IL-6, and TNF*-α* mRNA and/or protein levels in BV2 microglial cells and primary microglial cells. In LPS-treated C57BL/6N mice, selexipag administration significantly attenuated microgliosis/astrogliosis, proinflammatory mediator expression, and neuroinflammatory-associated dynamics molecules. In addition, selexipag treatment significantly inhibited LPS-induced NLRP3 inflammasome activation in BV2 microglial cells, primary microglial cells and C57BL/6N mice. Importantly, the anti-inflammatory effects of selexipag treatment in BV2 microglial cells were dependent on NLRP3. Moreover, selexipag administration significantly increased cAMP levels, decreased LPS-induced P38 phosphorylation, and suppressed LPS-induced proinflammatory responses via a P38-dependent manner in LPS-treated C57BL/6N mice and/or BV2 microglial cells. Taken altogether, the present results suggest that the selective IP receptor agonist selexipag may be a promising therapeutic candidate for mitigating neuroinflammation-associated neurological disorders.

## Introduction

Neuroinflammation, a fundamental pathological process characterized by sustained activation of glial cells in the central nervous system (CNS), is widely implicated in the development of neurodegenerative diseases, including Parkinson’s disease (PD) and Alzheimer’s disease (AD) (1, 2). Phenotypic shifts of microglia and astrocytes and their dynamic interactions play pivotal roles in CNS maintenance, including immune responses, integrity of the blood–brain barrier (BBB), and synapse formation and remodeling (3, 4). However, under pathological conditions such as infectious stimulation by lipopolysaccharide (LPS), disruption of glial homeostasis can exacerbate neuroinflammation that is associated with neurodegenerative disorder progression (5, 6). Persistent microglial activation induced by pathogens is associated with neurotoxicity through the excessive release of proinflammatory mediators, chemokines, and reactive oxygen species (ROS) (7, 8). Moreover, LPS-stimulated reactive astrocytes release inflammatory mediators and nitric oxide (NO), contributing to neuronal death (9, 10). Therefore, targeting the modulation of microglial and astrocytic activation to attenuate neuroinflammatory responses may represent a viable therapeutic strategy for neurological disorders associated with neuroinflammation.

The prostaglandin prostacyclin (IP) functions as an anti-inflammatory agent in cardiovascular and pulmonary diseases but exacerbates inflammatory responses in musculoskeletal diseases (11), suggesting that IP signaling can bidirectionally regulate inflammatory responses. Activation of the IP receptor stimulates cyclic adenosine monophosphate (cAMP) formation, which subsequently reduces proinflammatory mediator expression (12, 13). Interestingly, in a mouse model of pulmonary fibrosis, a synthetic analog of IP, iloprost, reduces the lung expression of proinflammatory and fibrotic mediators, such as TNF-α, TGF-1β, and IL-6, while increasing the expression of anti-inflammatory mediators (11). Selexipag, a selective IP receptor agonist approved by the FDA for the treatment of pulmonary arterial hypertension, has been shown to alleviate inflammation and vascular endothelial damage in LPS-induced respiratory distress syndrome by downregulating IL-1β, IL-6, and MCP-1 (14). Furthermore, MRE-269, the active metabolite of selexipag, reduces proinflammatory mediator expression and improves neurological function in aged rat models of ischemic stroke (15). These findings suggest that molecules that activate the IP receptor may have potential as therapeutic agents for reducing neuroinflammation.

The present study provides further support for the potential modulation of LPS-induced neuroinflammation in the CNS by selexipag based on the results of *in vitro* and *in vivo* experiments. Selexipag treatment significantly downregulated LPS-induced proinflammatory mediator levels in BV2 and primary microglial cells. In addition, selexipag pretreatment attenuated LPS-induced microgliosis, astrogliosis, and the expression of proinflammatory mediators and markers of reactive astrocytes/microglia associated with neurodegeneration in LPS-treated C57BL/6N mice. Moreover, selexipag treatment suppressed NLRP3 (NOD−like receptor family pyrin domain-containing 3) inflammasome activation in LPS-treated BV2 microglial cells, primary microglial cells, and/or C57BL/6N mice. Importantly, selexipag treatment reduced LPS-induced proinflammatory responses in an NLRP3-dependent manner in BV2 microglial cells. Furthermore, selexipag treatment increased cAMP levels, suppressed LPS-induced P38 phosphorylation, and downregulated LPS-induced proinflammatory responses in a P38-dependent manner. Collectively, these findings reveal a multifaceted modulation of LPS-induced neuroinflammatory responses by selexipag and underscore its potential as a therapeutic agent for inflammation-related neurological disorders.

## Materials and methods

### Ethics statement

All experiments were approved by the institutional biosafety committee (IBC) and performed in accordance with approved animal protocols of the Korea Brain Research Institute (KBRI, approval nos. IACUC-19-00049, IACUC-22-00044, and IACUC-24-00004).

### Selexipag

Selexipag was purchased from Selleckchem (Cat No. S3726, Houston, TX, USA). Lower doses (0.3 μM and 3.0 μM) of selexipag significantly decrease the expression of the proinflammatory mediator S100A4 in human systemic sclerosis (SSC) skin fibroblasts, whereas a higher dose (30 μM) has no effect on proinflammatory responses (16). Therefore, we chose selexipag doses of 0.5 μM, 1 μM, or 5 μM (in 1% DMSO) to investigate whether selexipag treatment modulates LPS-evoked inflammatory responses in BV2 or primary microglial cells. In addition, selexipag doses of 0.5 mg and 1.0 mg/kg (p.o., twice daily for 3 days) effectively alleviate LPS-mediated lung inflammation in mice, with superior regulatory effects of 1.0 mg/kg compared to 0.5 mg/kg (17). Therefore, we selected a dose of 1.0 mg/kg (dissolved in 1% DMSO in saline) as an optimal concentration for assessing the effects of selexipag treatment on LPS-mediated central neuroinflammation in C57BL/6N mice.

### BV2 microglial cells

BV2 microglial cells (Elabioscience Biotechnology Inc, Houston, TX, USA) were maintained as previously described (18). To examine the effect of selexipag on LPS-induced neuroinflammatory responses *in vitro*, BV2 microglial cells (2.0x10^5^/well) were treated with selexipag (0.5, 1 or 5 µM) or vehicle (1% DMSO) for 30 min and then challenged with LPS (200 ng/ml, L2630, *Escherichia coli*, Sigma-Aldrich, St. Louis, MO, USA) or PBS for 2.5 h, 5.5 h or 23.5 h.

### MTT assay

The 3-(4,5-dimethylthiazol-2-yl)-2,5-dipenyltetrazolium bromide (MTT; VWR Chemicals, Solon, OH, USA) assay was performed to evaluate the cytotoxicity of selexipag *in vitro*. BV2 microglial cells were incubated with vehicle (0.002, 0.01, 0.02, 0.05, or 0.1% DMSO) or selexipag (1, 5, 10, 25, or 50 µM) for 24 h. Next, the treated cells were incubated for 3 h with 0.4 mg/ml MTT solution. DMSO was added to dissolve the formazan crystal product, and 30 min later, the absorbance at 570 nm was measured in a SPECTROstar Nano microplate reader (BMG, Labtech, Ortenberg, Germany).

### ELISA in BV2 microglial cells

To examine the effect of selexipag treatment on LPS-induced proinflammatory responses at the protein level, BV2 microglial cells were treated with selexipag (0.5 µM) or vehicle (1% DMSO) for 30 min and then challenged with LPS (200 ng/ml) or PBS for 5.5 h. Next, the cells were lysed on ice for 5 min in lysis buffer (50 mM Tris, pH 7.4, 1% Triton X-100, 2 mM CaCl_2,_ 2 mM MgCl_2_) supplemented with protease inhibitor. The lysate was centrifuged at 12,000 rpm for 15 min, and protein assay reagents (Bio-Rad Laboratories, Hercules, CA, USA) were used to determine the protein concentration in the supernatant. COX-2, IL-1β, IL-6, and TNF-α protein levels were measured using a COX-2 ELISA kit (DYC4198-5; R&D Systems, Minneapolis, MN, USA) and IL-1β, IL-6, and TNF-α ELISA kits (88-7013A-88 for IL-1β, 88-7064–22 for IL-6, 88-7324–22 for TNF-α; Invitrogen, Waltham, Massachusetts, USA) according to the manufacturer’s instructions.

### Primary microglial cells

Primary glial cells were isolated from postnatal day 1 or 2 C57BL/6N mice according to an established protocol with minor modifications (19). Briefly, whole mouse brains were collected, mechanically dissociated, and filtered through 70-μm nylon mesh. The resulting mixed glial cultures (MGCs) were incubated in low-glucose DMEM (1000 mg/L glucose) supplemented with 10% FBS, 100 U/mL penicillin, and 100 μg/mL streptomycin. The MGCs were composed mainly of astrocytes and microglia and were maintained in a 5% CO_2_ incubator at 37 °C for 3 weeks. On day 21, the MGCs were subjected to mild trypsin digestion (0.5 M EDTA, 1 M CaCl_2_, and 0.25% trypsin–EDTA in low-glucose DMEM with 1% penicillin and streptomycin) for 40 min in a 5% CO_2_ incubator. After washing to remove the astrocytic layer, primary microglia were detached using trypsin–EDTA (0.25%) and centrifuged twice at 2000 rpm for 10 min before subsequent experiments. To investigate the effect of selexipag on LPS-induced neuroinflammatory responses, primary microglial cells (2.0x10^5^/well) were treated with selexipag (5 µM) or vehicle (1% DMSO) for 30 min and then stimulated with LPS (200 ng/ml) or PBS for 5.5 h.

### C57BL/6N mice

Eight-week-old C57BL/6N male mice (22–24 g; Koatech, Gyeonggi-do, Korea) were maintained in a pathogen-free facility with a 12-hr photoperiod in cages housing 4 mice each and access to food and water *ad libitum*. All mice were randomly assigned to treatment groups. To determine the effects of selexipag on acutely stimulated LPS-induced neuroinflammatory responses *in vivo*, we chose a high dose of LPS (10 mg/kg). In addition, since administration of 10 mg/kg LPS reduced the survival rate of mice 20 h after injection, we decided to euthanize the mice 8 h after LPS administration. Our previous studies confirmed that administration of 10 mg/kg LPS induces neuroinflammatory responses in the brains of C57BL/6N mice 8 h after injection (20–23).

We conducted two independent experiments to investigate the effect of selexipag *in vivo*. In the first experiment, C57BL/6N mice were injected (i.p.) with selexipag (1 mg/kg) or vehicle (1% DMSO) daily for 7 days. On day 7, the mice were treated (i.p.) with LPS (10 mg/kg) or PBS 30 min after the selexipag or vehicle (1% DMSO) injection. Eight hours after the LPS or PBS administration, the mice were sacrificed, and the brain was prepared for immunofluorescence (IF) staining. In the second experiment, C57BL/6N mice were treated as described above, and the bilateral cortical and the hippocampal regions were dissected for real-time PCR, western blotting, or ELISA. The dissected cortical and hippocampal tissues were stored at -80 °C until analysis.

### Immunofluorescence staining

To determine whether selexipag modulates neuroinflammatory responses *in vivo*, C57BL/6N mice were injected (i.p.) with selexipag (1 mg/kg) or vehicle (1% DMSO) daily for 7 days and sequentially treated with LPS (Sigma Aldrich, St. Louis, MO, USA, *Escherichia coli*,10 mg/kg, i.p.) or PBS for 8 hr. Subsequently, the mice were anesthetized with 2,2,2-tribromoethanol (2.5% v/v, 150 mg/kg, i.p., Sigma Aldrich, St. Louis, MO, USA) and transcardially perfused with PBS followed by 4% paraformaldehyde (PFA, Chembio, Seoul, Republic of Korea). The brains were removed, post-fixed in 4% PFA at 4 °C overnight and then cryoprotected in 30% sucrose at 4 °C for 2 days. Coronal brain sections (35 μm thick) were prepared using a Leica CM1850 cryostat (Wetzlar, Germany) and permeabilized for 1 h at room temperature in PBS containing 0.2% Triton X-100 (PBST) and 10% normal goat serum. The sections were immunostained for 24–72 h at 4 °C with anti-Iba-1, anti-GFAP, anti-COX-2, anti-IL-1β, or anti-TNF-α antibodies diluted in PBST, washed three times with PBST, and incubated with the secondary antibody at room temperature for 2 h. After a final three washes with PBS, the sections were mounted in DAPI-containing antifade mounting medium (Vector Laboratories, Burlingame, CA, USA). Images were acquired on a DMi8 fluorescence microscope (Leica Microsystems, Wetzlar, Germany) and analyzed by ImageJ (version 1.53e, National Institutes of Health, Bethesda, MD, USA). **Supplementary Table 1** provides the details of the primary and secondary antibodies. IF intensity was quantified using the results from 3–4 slices per mouse in each group (n = 5 mice per group x 3–4 slices, n = 19-20).

### Quantification of immunofluorescence staining

To measure the intensity of IF staining for Iba-1, GFAP, COX-2, IL-1β, and TNF-α in the cortex and/or hippocampal CA1, CA2, CA3, CA4 and/or DG regions of C57BL/6N mice, regions of interest (ROIs) were first selected in DAPI-stained images. Then, the intensity of IF staining for markers in each ROI was measured semi-automatically using the software ImageJ (version 1.53e, US National Institutes of Health, Bethesda, MD, USA). To quantify Iba-1- and GFAP-labeled regions of the brain, the cortical and hippocampal CA1 and DG regions were selected as the ROIs in DAPI-stained images, and the threshold for immune-reactivity was specified. The percentage immune-labeled area and the number of immune-positive cells in the selected regions were then calculated.

### Real-time PCR in BV2 microglial cells, primary microglial cells, and C57BL/6N mice

After treating BV2 microglial cells, primary microglial cells, or C57BL/6N mice with selexipag or vehicle and LPS as described above, TRIzol (Invitrogen, Waltham, MA, USA) was used to extract total RNA from cells or mouse brain tissue (cortex and hippocampus) according to the manufacturer’s recommendations. cDNA was reverse transcribed from the total RNA (1 μg) using the Superscript cDNA Premix Kit II with oligo (dT) primers (GeNetBio, Chungman, Korea). The cDNA was used as the template in real-time qPCR as described previously (20, 22). The sequences of the primers are provided in **Supplementary Table 2**. The GAPDH value was used to normalize cycle threshold (Ct) values, and fold changes were calculated relative to the control (vehicle-treated group).

### Target dependency validation via pharmacological inhibition of the IP receptor

To investigate the effect of an IP receptor antagonist itself on LPS-mediated proinflammatory mediator levels, BV2 microglial cells were treated with the IP receptor-selective antagonist BAY 73-1449 (0.01, 0.025 nM; MCE, Monmouth Junction, NJ, USA) or vehicle (1% DMSO) for 30 min followed by LPS (200 ng/ml) or PBS for 5.5 h, and real-time PCR was performed. Next, to determine if selexipag modulates LPS-induced neuroinflammation in an IP receptor (on-target)-dependent manner, BV2 microglial cells were treated first with BAY 73-1449 (1 μM (for ELISA) or 0.01 nM (for real-time PCR) MCE, Monmouth Junction, NJ, USA) or vehicle (1% DMSO) for 30 min, then with selexipag (5 µM) or vehicle (1% DMSO) treatment for 30 min, and finally with LPS (200 ng/ml) or PBS for 5.5 h. After confirming suppression of cAMP levels (IP receptor downstream signaling) by ELISA, proinflammatory mediator mRNA levels were measured by real-time PCR.

### P38 inhibition in BV2 microglial cells

To assess whether the anti-inflammatory effects of selexipag treatment against LPS treatment are mediated by suppression of P38 activity, BV2 microglial cells were treated first with the P38 antagonist SB305-580 (10 μM, Calbiochem, Sandiego, CA, USA) or vehicle (1% DMSO) for 30 min, then with selexipag (5 µM) or vehicle (1% DMSO) treatment for 30 min, and finally with LPS (200 ng/ml) or PBS for 5.5 h. Then, proinflammatory mediator mRNA levels were measured by real-time PCR.

### c-Jun inhibition in BV2 microglial cells

To investigate whether selexipag treatment downregulates LPS-stimulated proinflammatory responses via c-Jun, BV2 microglial cells were treated first with the c-Jun-selective antagonist c-Jun peptide (100 μM, MCE, Monmouth Junction, NJ, USA) or vehicle (D.W.) for 30 min, then with selexipag (5 µM) or vehicle (1% DMSO) treatment for 30 min, and finally with LPS (200 ng/ml) or PBS for 5.5 h. Proinflammatory mediator mRNA levels were measured by real-time PCR.

### cAMP ELISA in BV2 microglial cells and C57BL/6N mice

To examine intracellular cAMP levels, BV2 microglial cells treated as described above were lysed in 0.1 M HCl for 10 min at room temperature, and the lysate was centrifuged for 20 min at 4 °C and 12000 rpm. In addition, the cortical and hippocampus regions from C57BL/6N mice treated as described above were dissected and homogenized in RIPA lysis buffer (Merck Millipore, Billerica, MA, USA) containing protease and phosphatase inhibitor cocktail (1%; Thermo Scientific, Waltham, MA, USA). After incubation on ice for 1 h at 4 °C, the lysate was centrifuged for 20 min at 4 °C and 12000 rpm. For both lysates, the supernatant was collected, and cAMP levels were measured by ELISA (cAMP complete ELISA kit, ADI-901-163, Enzo Life Sciences, Farmingdale, NY, U.S.A.) according to the manufacturer’s instructions.

### Western blotting in BV2 microglial cells and C57BL/6N mice

To assess the effect of selexipag treatment on LPS-induced NLRP3, p-P38/P38, p-ERK/ERK, p-c-Jun^Ser73^/c-Jun and/or p-STAT3^Y705^/STAT3 levels, BV2 microglial cells treated as described above were harvested in lysis buffer (50 mM Tris, pH 7.4, 1% Triton X-100, 2 mM CaCl_2_ and 2 mM MgCl_2_) with 1% protease inhibitor and phosphatase inhibitors (Thermo Scientific, Waltham, MA, USA), and the lysate was centrifuged for 20 min at 4 °C and 12000 rpm. In addition, the bilateral cortex and hippocampus from C57BL/6N mice treated as described above were dissected and homogenized in RIPA lysis buffer (Merck Millipore, Billerica, MA, USA) supplemented with protease and phosphatase inhibitor cocktail (1%; Thermo Scientific, Waltham, MA, USA). After incubation on ice for 1 h at 4 °C, the lysate was centrifuged for 20 min at 4 °C and 12000 rpm. For both lysates, the supernatant was collected, and the protein concentration was quantified by the BCA method relative to a standard solution of BSA. Next, 10 µg of protein sample was loaded on an 8% SDS-PAGE gel, separated by electrophoresis, and transferred to a polyvinylidene difluoride (PVDF) membrane. After blocking with 5% skim milk at room temperature for 1 h, the membrane was incubated with primary antibody overnight at 4 °C. The next day, the membrane was incubated with the corresponding secondary antibody for 1 h, and then detection was realized by adding ECL Western Blotting Detection Reagent (GE Healthcare, Chicago, IL, USA). Images were acquired and analyzed by Fusion Capt Advance software (Vilber Lourmat). The primary and secondary antibodies used for western blotting are listed in **Supplementary Table 3**.

### siRNA transfection in BV2 microglial cells

To investigate whether the effects of selexipag treatment on LPS-induced neuroinflammatory responses are dependent on in its neuroinflammation-associated molecular target, NLRP3, BV2 microglial cells were transfected with small interfering RNA (siRNA) targeting mouse NLRP3 (Vector Biolabs, Malvern, PA, USA) as previously described with minor modifications (20). Briefly, NLRP3 siRNA or scramble siRNA was diluted in Opti-MEM medium (Thermo Scientific, Waltham, MA, USA) to a concentration of 400 nM, and Lipofectamine^®^ RNAiMAX reagent (Thermo Scientific, Waltham, MA, USA) was added. Transfection of BV2 microglial cells with the siRNA complex suspension (final siRNA concentration at 40 nM) was performed as previously described (18). Forty-one hour after transfection, the BV2 microglial cells were incubated in serum-free media for 1 h and then treated with selexipag (5 μM) or vehicle (1% DMSO) for 30 min followed by LPS (200 ng/ml) or PBS for 5.5 h. Finally, the BV2 microglial cells were harvested, and NLRP3 knockdown efficiency was analyzed by real-time PCR. After validating NLRP3 siRNA transfection, proinflammatory mediator mRNA levels were measured by real-time PCR.

### Statistical analysis

GraphPad Prism 10 software (GraphPad Software, San Diego, CA, USA) was used for graph generation and statistical analysis. Data are presented as individual data points and means ± standard errors of measurement (SEMs). For comparisons between two groups, Student’s *t-*test was used. For multiple comparisons, one-way analysis of variance (ANOVA) with Tukey’s *post hoc* or Newman-Keuls analysis was used. Asterisks indicate significance: *p < 0.05, **p < 0.01, and ***p < 0.001. A detailed statistical analysis is provided in **Supplementary Table 4**.

## Results

### Selexipag treatment suppresses LPS-induced proinflammatory mediator expression in BV2 microglial cells

**Figure 1:**
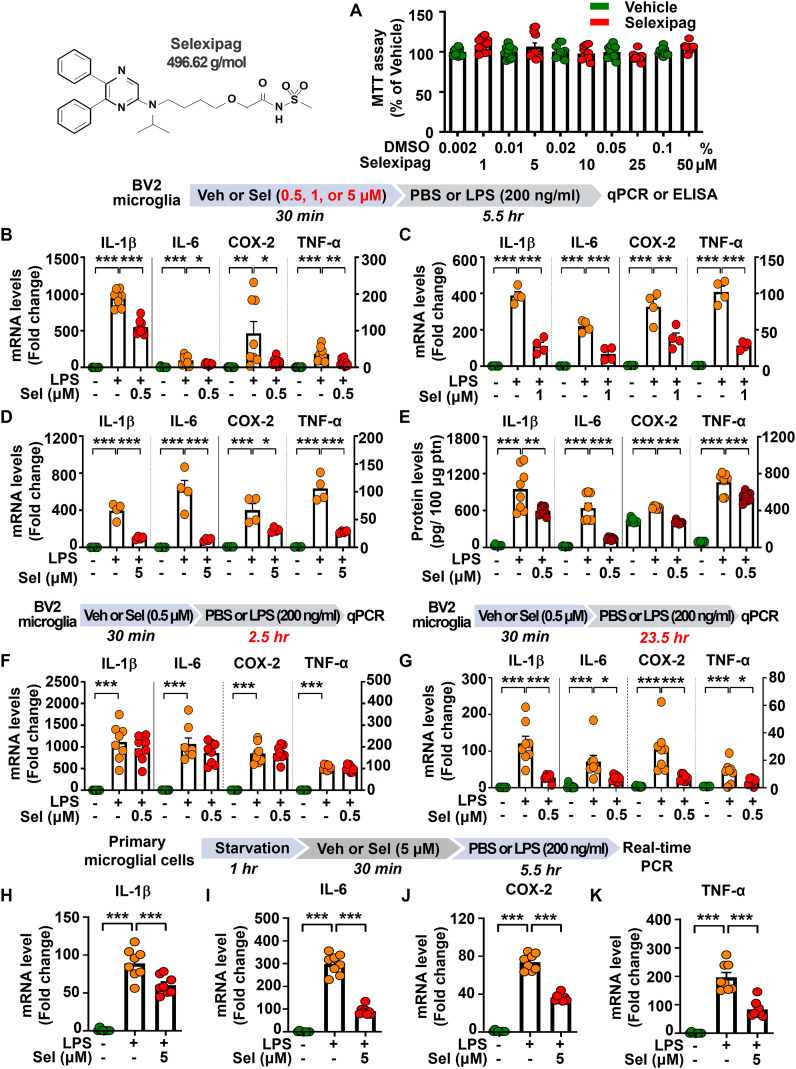
Selexipag treatment attenuates the induction of proinflammatory mediators by LPS in BV2 microglial cells and/or primary microglial cells. **(A)** MTT assays of the cytotoxicity of selexipag in BV2 microglial cells treated with vehicle (0.002%-0.1% DMSO) or selexipag (1, 5, 10, 25, or 50 μM) for 24 h (n = 11-12/group). **(B-D)** Real-time PCR of proinflammatory mediator mRNA expression in BV2 microglial cells treated with vehicle (1% DMSO) or 0.5, 1, or 5 µM selexipag for 30 min and then treated with LPS (200 ng/ml) or PBS for 5.5 h (n = 4-8/group). **(E)** ELISA of proinflammatory mediator expression in BV2 microglial cells treated with vehicle (1% DMSO) or 0.5 µM selexipag for 30 min and then treated with LPS (200 ng/ml) or PBS for 5.5 h (n =8/group). **(F)** Real-time PCR of proinflammatory mediator mRNA expression in BV2 microglial cells treated with vehicle (1% DMSO) or 0.5 µM selexipag for 30 min and then treated with LPS (200 ng/ml) or PBS for 2.5 h (n = 8/group). **(G)** Real-time PCR of proinflammatory mediator mRNA expression in BV2 microglial cells treated with vehicle (1% DMSO) or 0.5 µM selexipag for 30 min and then treated with LPS (200 ng/ml) or PBS for 23.5 h (n = 7-8/group). **(H-K)** Real-time PCR of proinflammatory mediator mRNA levels in primary microglial cells treated with vehicle (1% DMSO) or 5 µM selexipag for 30 min and then treated with PBS or LPS (200 ng/ml) for 5.5 h (n = 8/group). *p < 0.05, **p < 0.01, ***p < 0.001.

Before examining the effects of selexipag treatment on LPS-induced proinflammatory responses, we first evaluated its cytotoxicity *in vitro*. For this experiments, BV2 microglial cells were treated with vehicle (0.002, 0.01, 0.02, 0.05, or 0.1% DMSO) or selexipag (1, 5, 10, 25, or 50 µM) for 24 h, and cell viability was measured using the MTT assay. We found that selexipag treatment had no cytotoxic effects in BV2 microglial cells, even at the highest concentration (50 µM), compared with vehicle treatment (**Figure 1A**).

To investigate whether selexipag treatment affects LPS-evoked proinflammatory mediator levels in a dose-dependent manner *in vitro*, BV2 microglial cells were treated first with 0.5 µM, 1 µM or 5 µM selexipag or vehicle (1% DMSO) for 30 min and then with 200 ng/ml LPS or PBS for 5.5 h. Next, proinflammatory mediator mRNA levels were quantified by real-time PCR. All three doses of selexipag significantly reduced the LPS-induced proinflammatory mediators IL-1β, IL-6, COX-2, and TNF-α mRNA levels (**Figures 1B–D**).

Next, we performed ELISA to examine whether selexipag treatment alters the protein levels of LPS-induced proinflammatory mediators. To test this, BV2 microglial cells were treated with 0.5 µM selexipag or vehicle (1% DMSO) for 30 min, followed by 200 ng/ml LPS or PBS for 5.5 h, and the protein levels of IL-1β, IL-6, COX-2, and TNF-α were quantified by ELISA. Treatment with 0.5 µM selexipag significantly reduced the LPS-induced protein levels of the proinflammatory mediators IL-1β, IL-6, COX-2, and TNF-α in BV2 microglial cells (**Figure 1E**).

We then investigated the effects of selexipag on LPS-mediated proinflammatory responses in a time dependent manner. For these experiments, BV2 microglial cells were treated with 0.5 µM selexipag or vehicle (1% DMSO) for 30 min, followed by 200 ng/ml LPS or PBS for 2.5 h or 23.5 h. Quantification of IL-1β, IL-6, COX-2, and TNF-α mRNA levels by real-time PCR showed that treatment with We found that selexipag (0.5 µM) did not affect LPS-induced proinflammatory mediators mRNA expression when the total treatment time was for 3 h (**Figure 1F**), whereas the LPS-induced mRNA levels of the proinflammatory mediators IL-1β, IL-6, COX-2, and TNF-α were significantly reduced when the total exposure time to selexipag was 24 h (**Figure 1G**). These results suggest that selexipag attenuates LPS-induced proinflammatory mediator expression in BV2 microglial cells in a time-dependent manner.

### Selexipag treatment reduces LPS-induced proinflammatory mediator levels in primary microglial cells

To examine whether selexipag treatment modulates LPS-induced neuroinflammatory responses in primary microglial cells, the cells were treated with 5 µM selexipag or vehicle (1% DMSO) for 30 min, followed by treatment with 200 ng/ml LPS or PBS for 5.5 h. Subsequent real-time PCR analysis showed that selexipag treatment significantly reduced LPS-induced IL-1β, IL-6, COX-2, and TNF-α mRNA levels in primary microglial cells (**Figures 1H–K**), indicating that selexipag treatment diminishes LPS-mediated proinflammatory responses in primary microglial cells.

### Selexipag administration alleviates microgliosis and astrogliosis in LPS-treated C57BL/6N mice

**Figure 2:**
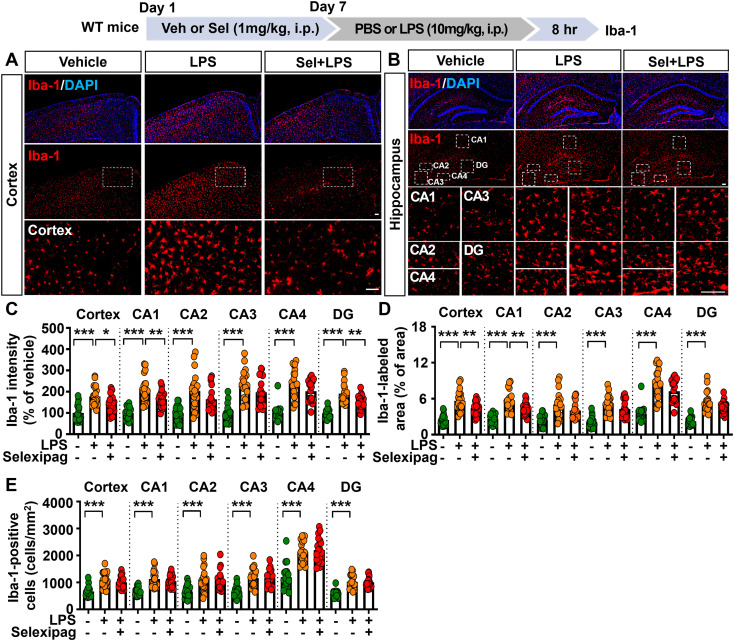
Selexipag administration suppresses LPS-mediated microglial activation and hypertrophy but not microglial migration in C57BL/6N mice. **(A, B)** Representative immunofluorescence staining with an anti-Iba-1 antibody of brain sections from C57B/L6 mice injected (i.p.) with vehicle (1% DMSO) or 1 mg/kg selexipag daily for 7 days, followed by LPS (10 mg/kg) or PBS on day 7. **(C-E)** Quantification of data from **(A, B)** (n = 19–20 brain sections from 5 mice/group). *p < 0.05, **p < 0.01, ***p < 0.001. Scale bar = 200 µm.

Since selexipag treatment downregulated LPS-induced proinflammatory mediator levels in BV2 microglial cells and primary microglial cells, we assessed the effects of selexipag administration on LPS-induced microglial activation *in vivo*. C57BL/6N mice were injected with 1 mg/kg selexipag (i.p.) or vehicle (1% DMSO) daily for 7 days; on day 7, the mice were injected with 10 mg/kg LPS (i.p.) or PBS after selexipag or vehicle injection. Subsequent IF staining of brain sections with an anti-Iba-1 antibody demonstrated that selexipag treatment reduced the LPS-induced increases in Iba-1 fluorescence intensity in the cortex and hippocampal CA1 and DG regions but not in the hippocampal CA2, CA3 and CA4 regions (**Figures 2A–C**). In addition, selexipag administration significantly reduced the LPS-induced Iba-1-labeled area in the cortex and hippocampal CA1 region, whereas no significant changes were observed in the hippocampal CA2, CA3, CA4 and DG regions (**Figures 2A, B, D**). However, selexipag treatment did not alter the LPS-induced increase in Iba-1-positive cell numbers in the brain (**Figures 2A, B, E**). These results demonstrate that selexipag selectively attenuates LPS-induced microglial activation in brain subregions rather than exerting a uniform effect throughout the hippocampus.

**Figure 3:**
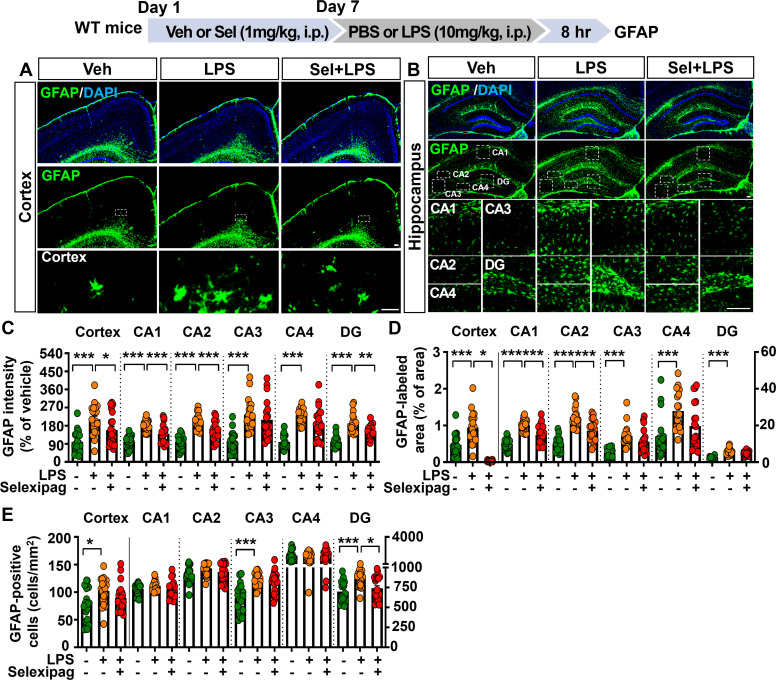
Selexipag treatment reduces LPS-induced astroglial activation, hypertrophy, and migration in C57BL/6N mice. **(A, B)** Representative images of immunofluorescence staining with an anti-GFAP antibody of brain sections from C57BL/6N mice injected (i.p.) with vehicle (1% DMSO) or 1 mg/kg selexipag daily for 7 days, followed by LPS (10 mg/kg) or PBS on day 7. **(C-E)** Quantification of data from **(A, B)** (n = 19–20 brain sections from 5 mice/group). *p < 0.05, **p < 0.01, ***p < 0.001. Scale bar = 200 µm.

We then examined the effects of selexipag treatment on astrogliosis in LPS-treated C57BL/6N mice. IF staining with an anti-GFAP antibody showed that selexipag treatment significantly suppressed LPS-induced GFAP fluorescence intensity in cortical layers III–V and the hippocampal CA1, CA2, and DG regions but not in the hippocampal CA3 and CA4 regions (**Figures 3A–C**). In addition, selexipag treatment significantly downregulated the LPS-induced increase in GFAP-labeled area in cortical layers III–V and the hippocampal CA1 and CA2 regions but not in the hippocampal CA3, CA4, and DG regions (**Figures 3A, B, D**). Moreover, selexipag treatment significantly decreased the number of GFAP-positive cells in the hippocampal DG region but not in cortical layers III–V and the hippocampal CA1–CA4 regions compared with mice treated with vehicle (**Figures 3A, B, E**). Collectively, these results suggest that selexipag treatment diminishes LPS-induced astrocyte activation, hypertrophy, and migration in the brain in C57BL/6N mice.

### Selexipag treatment decreases proinflammatory mediator expression in LPS-treated C57BL/6N mice

**Figure 4:**
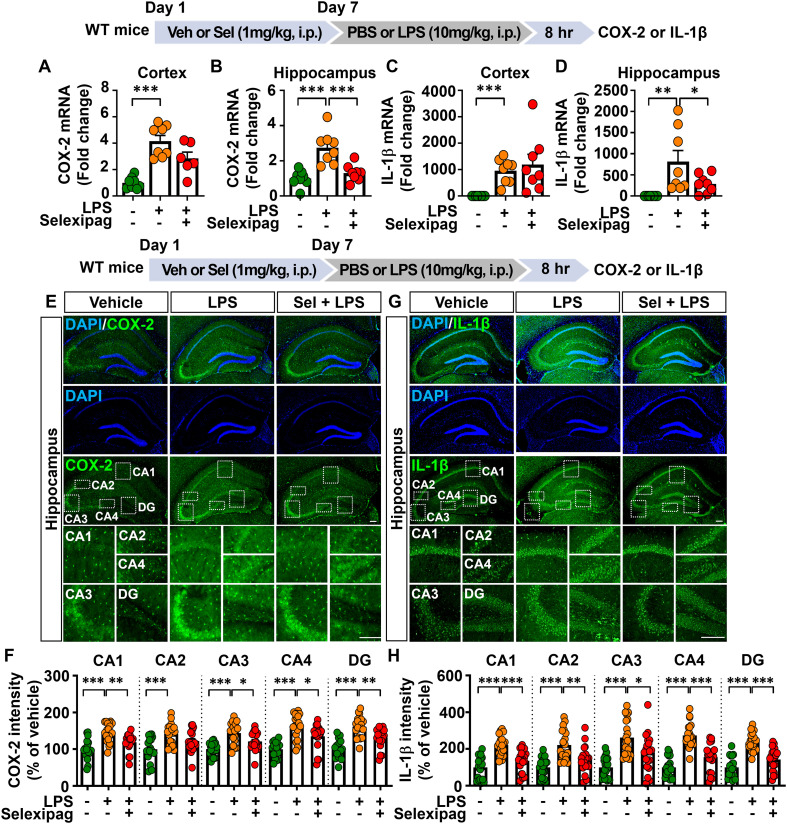
Selexipag administration alleviates LPS-evoked proinflammatory mediator COX-2 and IL-1β expression in C57BL/6N mice. **(A-D)** Real-time PCR of COX-2 and IL-1β mRNA expression in C57BL/6N mice injected (i.p.) with vehicle (1% DMSO) or 1 mg/kg selexipag daily for 7 days, followed by LPS (10 mg/kg) or PBS on day 7 (n = 6-8/group). **(E, G)** Representative images of immunofluorescence staining with anti-COX-2 and anti-IL-1β antibodies of brain sections from C57BL/6N mice treated as described above. **(F, H)** Quantification of data from **(E, G)**, respectively (n = 19–20 brain sections from 5 mice/group). *p < 0.05, **p < 0.01, ***p < 0.001. Scale bar = 200 µm.

Since selexipag treatment abolished LPS-induced microglial and astrocyte activation in C57BL/6N mice, we next investigated the effects of selexipag treatment on LPS-induced proinflammatory mediator levels *in vivo*. C57BL/6N mice were treated as described above, and the cortex and hippocampus were dissected for real-time PCR analysis. The results revealed that selexipag treatment significantly reduced LPS-induced COX-2 and IL-1β mRNA levels in the hippocampus but not in the cortex (**Figures 4A–D**).

To validate these findings, IF staining of brain sections with an anti-COX-2 antibody was performed. The results demonstrated that selexipag treatment significantly attenuated LPS-induced COX-2 fluorescence intensity in the hippocampal CA1, CA3, CA4 and DG regions but not in the hippocampal CA2 region (**Figures 4E, F**). Additionally, selexipag treatment significantly decreased the LPS-induced increase in IL-1β fluorescence intensity in the hippocampus (**Figures 4G, H**).

**Figure 5:**
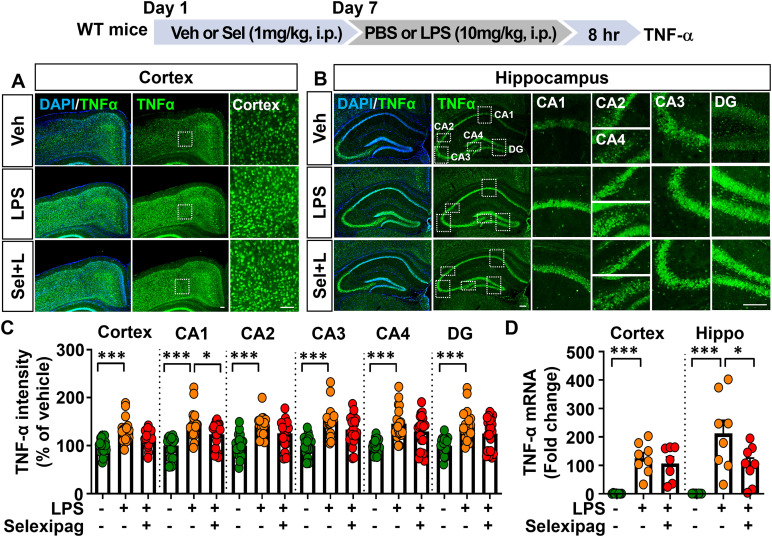
Selexipag treatment attenuates LPS-induced proinflammatory cytokine TNF-α expression in C57BL/6N mice. **(A, B)** Representative images of immunofluorescence staining with an anti-TNF-α antibody of brain sections from C57BL/6N mice injected (i.p.) with vehicle (1% DMSO) or 1 mg/kg selexipag daily for 7 days, followed by LPS (10 mg/kg) or PBS on day 7 (n = 7-8/group). **(C)** Quantification of data from **(A, B)** (n = 19–20 brain sections from 5 mice/group). **(D)** Real-time PCR quantification of TNF-α mRNA expression in C57BL/6N mice treated as described above (n = 7-8/group). *p < 0.05, ***p < 0.001. Scale bar = 200 µm.

We then examined whether selexipag treatment affects the levels of other proinflammatory cytokine in LPS-treated C57BL/6N mice by performing IF staining and real-time PCR. IF staining with an anti-TNF-α antibody showed that selexipag treatment significantly attenuated LPS-induced TNF-α levels only in the hippocampal CA1 region and not in the hippocampal CA2, CA3, CA4, and DG regions or cortex (**Figures 5A–C**). Furthermore, selexipag administration significantly suppressed LPS-induced TNF-α mRNA levels in the hippocampus but not the cortex (**Figure 5D**). These data indicate that selexipag treatment attenuates proinflammatory mediator COX-2, IL-1β and TNF-α mRNA and protein expression in LPS-treated C57BL/6N mice.

### Selexipag administration downregulates LPS-induced microglial and astroglial-associated neuroinflammatory dynamics in C57BL/6N mice

**Figure 6:**
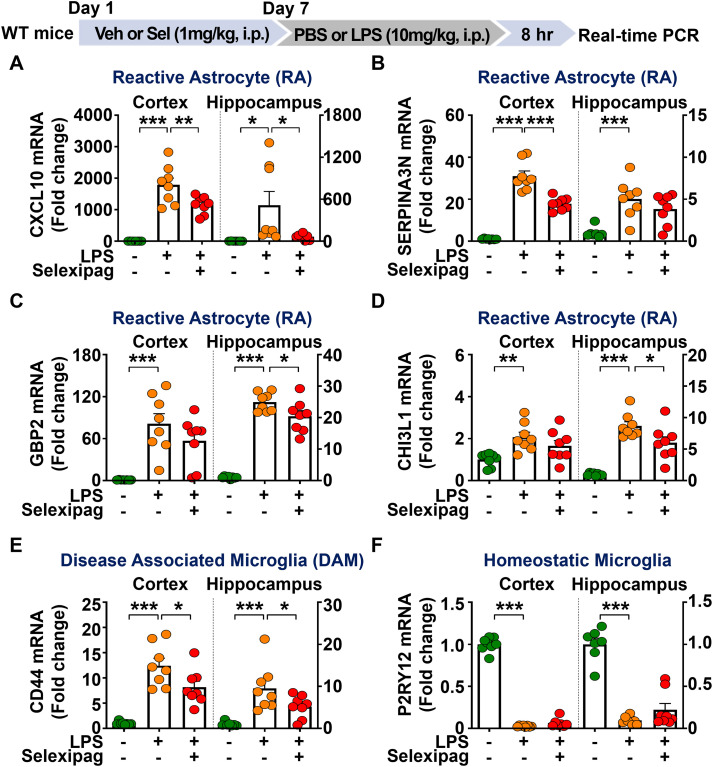
Selexipag administration reduces LPS-mediated microglia- and astroglia-associated neuroinflammatory dynamics in C57BL/6N mice. **(A-F)** Real-time PCR of the mRNA expression of microglia- and astroglia-associated neuroinflammatory dynamics markers in C57BL/6N mice injected (i.p.) with vehicle (1% DMSO) or 1 mg/kg selexipag daily for 7 days, followed by LPS (10 mg/kg) or PBS on day 7 (n = 7-8/group). *p < 0.05, **p < 0.01, ***p < 0.001.

Since selexipag treatment significantly reduced LPS-induced microglial/astroglial activation and proinflammatory mediator levels in C57BL/6N mice, we examined its effects on the expression of markers of microglia- and astroglia-associated neuroinflammatory dynamics *in vivo*. To address this, C57BL/6N mice were treated as described above, and the cortex and hippocampus were dissected for real-time PCR analysis. We found that selexipag treatment significantly decreased LPS-induced CXCL10 [a marker of reactive astrocytes (RAs)] mRNA levels in the cortex and hippocampus (**Figure 6A**). In addition, selexipag treatment significantly downregulated LPS-mediated SERPINA3N [a marker of reactive astrocytes (RAs)] mRNA levels in the cortex but not the hippocampus (**Figure 6B**). Moreover, selexipag treatment downregulated LPS-induced GBP2 and CHI3L1 (markers of RAs) mRNA levels in the hippocampus but not the cortex (**Figures 6C, D**).

Next, we assessed the effects of selexipag treatment on microglia-associated neuroinflammatory dynamics in LPS-treated C57BL/6N mice. The results of real-time PCR showed that selexipag treatment significantly reduced LPS-induced CD44 [a marker of disease-associated microglia (DAMs)] mRNA levels in the cortex and hippocampus (**Figure 6E**). However, selexipag treatment did not alter P2RY12 (a marker of homeostatic microglia) mRNA levels in the cortex and hippocampus (**Figure 6F**). These data suggest that selexipag treatment selectively reduces LPS-induced microglia- and astrocyte-associated neuroinflammatory dynamics-related mRNA levels in C57BL/6N mice.

### Selexipag administration attenuates LPS-mediated NLRP3 inflammasome activation *in vivo* and *in vitro*

**Figure 7:**
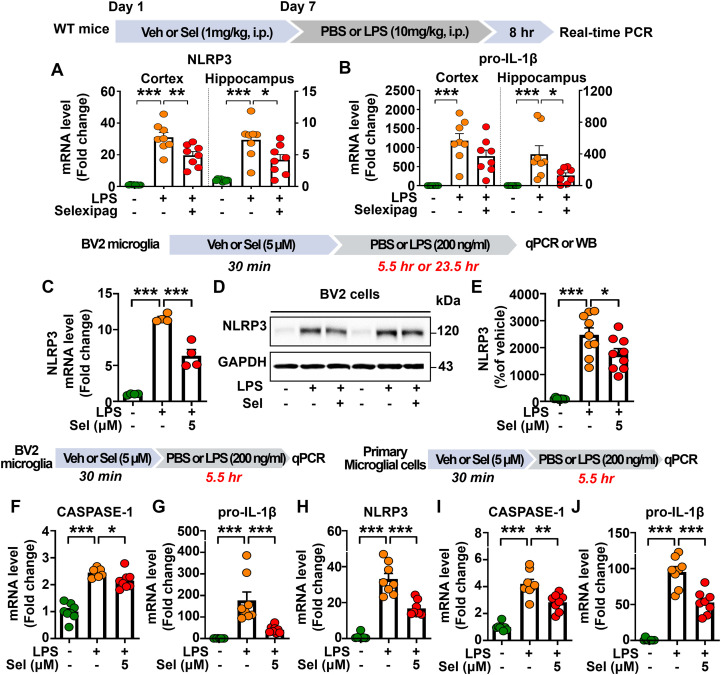
Selexipag treatment attenuates the LPS-evoked NLRP3 inflammasome activation *in vivo* and *in vitro*. **(A, B)** Real-time PCR of NLRP3 and pro-IL-1β mRNA expression in C57BL/6N mice injected (i.p.) with vehicle (1% DMSO) or 1 mg/kg selexipag daily for 7 days, followed by LPS (10 mg/kg) or PBS on day 7 (n = 8/group). **(C)** Real-time PCR of NLRP3 mRNA expression in BV2 microglial cells treated with vehicle (1% DMSO) or selexipag (5 µM) for 30 min and then treated with PBS or LPS (200 ng/ml) for 5.5 h (n = 4/group). **(D, E)** Western blot analysis with an anti-NLRP3 antibody of BV2 microglial cells treated with vehicle (1% DMSO) or selexipag (5 µM) for 30 min and then treated with PBS or LPS (200 ng/ml) for 23.5 h (n = 9/group). **(F, G)** Real-time PCR of CASPASE-1 and pro-IL-1β mRNA expression in BV2 microglial cells treated with vehicle (1% DMSO) or selexipag (5 µM) for 30 min and then treated with PBS or LPS (200 ng/ml) for 5.5 h (n = 7-8/group). **(H)** Real-time PCR of NLRP3 mRNA expression in primary microglial cells treated with vehicle (1% DMSO) or selexipag (5 µM) for 30 min and then treated with PBS or LPS (200 ng/ml) for 5.5 h (n = 8/group). **(I, J)** Real-time PCR of CASPASE-1 and pro-IL-1β mRNA expression in primary microglial cells treated as described above (n = 8/group). *p < 0.05, **p<0.01, ***p < 0.001.

To determine the mechanisms by which selexipag alters neuroinflammatory responses *in vivo*, C57BL/6N mice were treated as described above, and the cortex and hippocampus were dissected for real-time PCR analysis. We found that selexipag treatment significantly downregulated LPS-induced NLRP3 mRNA levels in the cortex and hippocampus (**Figure 7A**) and reduced LPS-induced pro-IL-1β mRNA levels only in the hippocampus (**Figure 7B**).

Next, we assessed whether selexipag affects LPS-mediated NLRP3 protein expression and NLRP3 inflammasome activation *in vitro*. BV2 microglial cells were treated with vehicle (1% DMSO) or selexipag (5 μM) for 30 min, followed by treatment with PBS or LPS (200 ng/ml) for 5.5 or 23.5 h. Analysis by western blotting and real-time PCR showed that selexipag treatment significantly downregulated LPS-induced NLRP3 mRNA and protein levels in BV2 microglial cells (**Figures 7C–E**). In addition, we found that selexipag-treated BV2 microglial cells significantly decreased LPS-mediated caspase-1 and pro-IL-1β mRNA levels (**Figures 7F, G**).

We then investigated the effects of selexipag treatment on NLRP3 inflammasome activation in primary microglial cells treated with vehicle (1% DMSO) or selexipag (5 μM) for 30 min followed by LPS (200 ng/ml) or PBS for 5.5 h. We found that selexipag treatment significantly reduced LPS-induced NLRP3, CASPASE-1 and pro-IL-1β mRNA levels in primary microglial cells (**Figures 7H–J**).

### Selexipag treatment diminishes LPS-evoked proinflammatory responses through NLRP3

In *C. albicans-*infected macrophages, the IP receptor antagonist CAY10441 decreases COX-2 protein levels, consistent with COX-2’s role as a principal enzyme in prostacyclin (PGI_2_) biosynthesis (24, 25). Since the IP receptor agonist selexipag significantly suppressed LPS-induced proinflammatory mediator levels *in vitro* and *in vivo*, we wanted to determine if these effects are modulated via the IP receptor (on-target of selexipag). To address this, we first investigated whether IP receptor antagonist treatment alters LPS-induced proinflammatory mediator levels in BV2 microglial cells. BV2 microglial cells were treated with vehicle (1% DMSO) or the selective IP receptor antagonist BAY 73-1449 (0.01 or 0.025 nM) for 30 min followed by PBS or LPS (200 ng/mL) for 5.5 h, and real-time PCR was conducted. We found that BAY 73–1449 and LPS (BAY 73-1449 + LPS) treatment significantly decreased LPS-induced COX-2 mRNA levels in BV2 microglial cells but not IL-1β mRNA levels (**Supplementary Figures 2A, B**).

Given that BAY 73–1449 treatment suppresses cAMP signaling, downstream of the IP receptor (26), we tested whether BAY 73–1449 administration modulates intracellular cAMP levels in LPS-treated BV2 microglial cells. To test this, BV2 microglial cells were first treated with vehicle (1% DMSO) or BAY 73-1449 (1 μM) for 30 min, then with vehicle (1% DMSO) or selexipag (5 μM) for 30 min, and finally with PBS or LPS (200 ng/mL) for 5.5 h. We found that IP receptor agonist selexipag and LPS (selexipag + LPS) treatment significantly increased cAMP levels compared to LPS-treated BV2 microglial cells. By contrast, BAY 73–1449 and LPS (BAY 73-1449 + LPS) treatment significantly reduced cAMP levels compared to LPS treatment in BV2 microglial cells, confirming successful blockade of IP receptor signaling by BAY 73–1449 treatment (**Supplementary Figure 2C**).

Next, to investigate whether selexipag modulates LPS-mediated IL-1β mRNA levels through the IP receptor, BV2 microglial cells were treated first with vehicle (1% DMSO) or BAY 73-1449 (0.01 nM) for 30 min, then with vehicle (1% DMSO) or selexipag (5 μM) for 30 min, and finally with PBS or LPS (200 ng/mL) for 5.5 h. We found that treatment with selexipag and LPS (Selexipag + LPS) significantly reduced LPS-evoked IL-1β mRNA levels in BV2 microglial cells compared with treatment with LPS alone (**Supplementary Figure 2D**). Importantly, BAY 73-1449, selexipag and LPS (BAY 73-1449 + selexipag + LPS) treatment did not further decrease IL-1β mRNA levels compared to treatment with BAY 73–1449 and LPS (BAY 73-1449 + LPS or treatment with Selexipag and LPS (Selexipag + LPS) in BV2 microglial cells (**Supplementary Figure 2D**). These findings suggest that selexipag downregulates LPS-induced IL-1β mRNA levels in an IP receptor (on target)-dependent manner.

**Figure 8:**
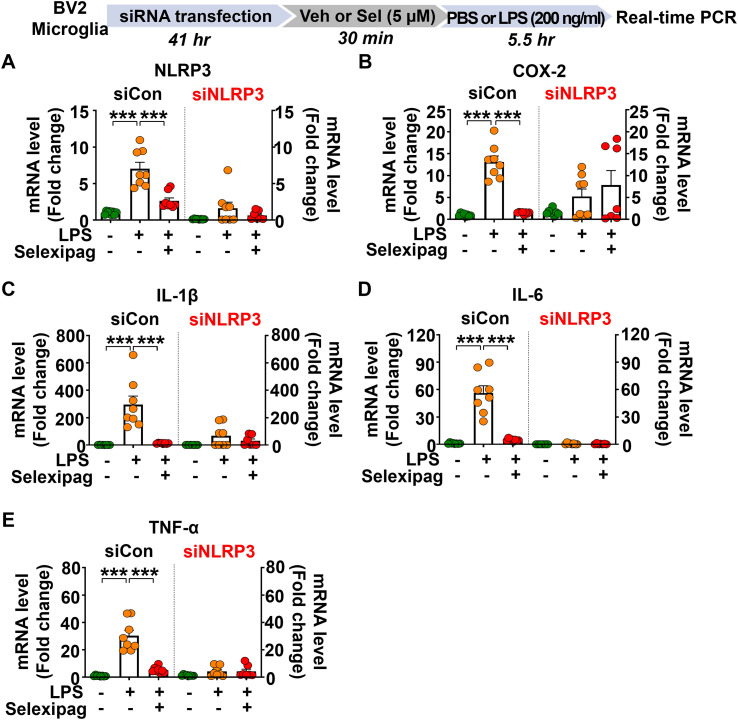
Selexipag administration reduces LPS-induced proinflammatory mediator levels via NLRP3 in BV2 microglial cells. **(A-E)** Real-time PCR of NLRP3, COX-2, IL-1β, IL-6 and TNF-α mRNA expression in BV2 microglial cells transfected with NLRP3 siRNA (40 nM) or scramble siRNA and subsequently treated with vehicle (1% DMSO) or selexipag (5 µM) for 30 min followed by PBS or LPS (200 ng/ml) for 5.5 h (n =7- 8/group). ***p < 0.001.

To determine whether the neuroinflammatory effects of selexipag are NLRP3 dependent, BV2 microglial cells were transfected with either NLRP3 siRNA or scramble siRNA complex for 41 h and then incubated in serum-free media for 1 h. Next, the cells were treated with 5 μM selexipag or vehicle (1% DMSO) for 30 min followed by LPS (200 ng/ml) or PBS for 5.5 h. Real-time PCR analysis showed that NLRP3 siRNA transfection reduced NLRP3 mRNA levels in BV2 microglial cells by 71.69% compared with scramble siRNA transfection (**Figure 8A**). We then examined whether NLRP3 silencing influences the effect of selexipag on LPS-induced proinflammatory responses in BV2 microglial cells. We found that selexipag treatment significantly reduced LPS-induced COX-2, IL-1β, IL-6 and TNF-α mRNA levels in scramble (control) siRNA-transfected BV2 microglial cells compared to LPS treatment (**Figures 8B–E**). By contrast, in NLRP3 siRNA-transfected BV2 microglial cells, selexipag treatment did not alter LPS-mediated COX-2, IL-1β, IL-6 and TNF-α mRNA levels compared to LPS treatment (**Figures 8B–E**). These data suggest that selexipag treatment downregulates LPS-evoked proinflammatory responses in BV2 microglial cells via an NLRP3-dependent mechanism.

### Selexipag administration significantly increases cAMP levels and inhibits LPS-mediated P38 signaling in C57BL/6N mice or BV2 microglial cells

**Figure 9:**
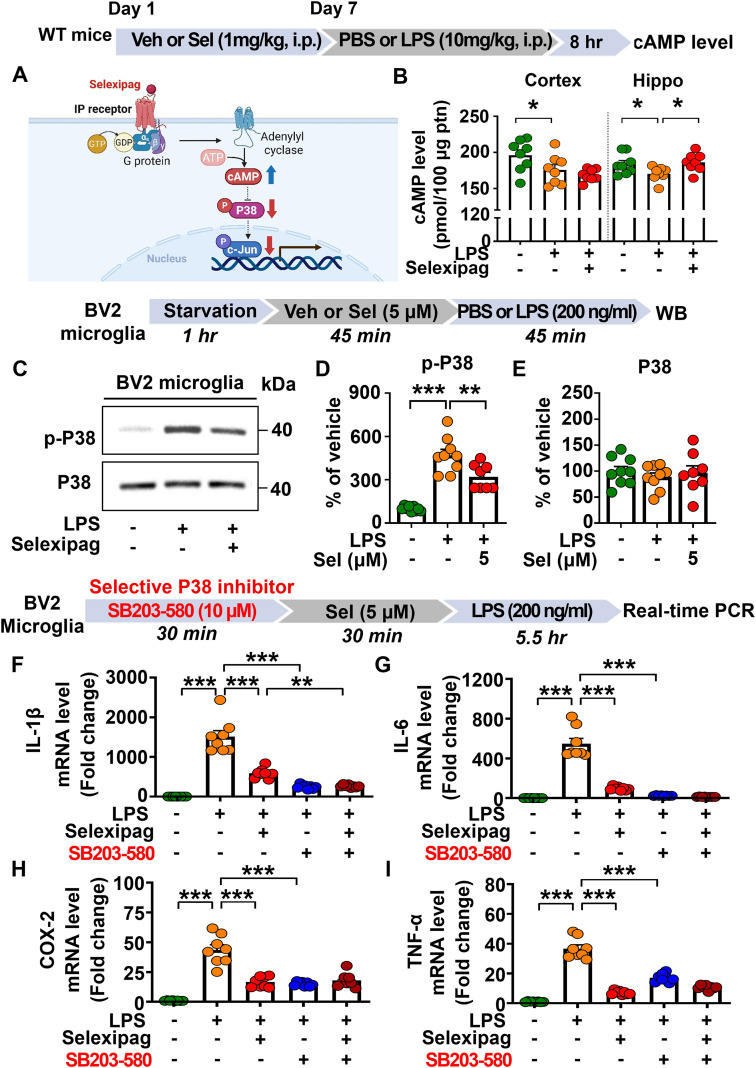
Selexipag treatment enhances cAMP levels and suppresses P38 phosphorylation in LPS-treated C57BL/6N mice and/or BV2 microglial cells. **(A)** Schematic illustration of the IP receptor and its downstream cAMP-P38 signaling pathway. **(B)** ELISA of cAMP levels in the cortex and hippocampus of C57BL/6N mice injected (i.p.) with vehicle (1% DMSO) or 1 mg/kg selexipag daily for 7 days, followed by 10 mg/kg LPS or PBS on day 7 (n = 8/group). **(C-E)** Western blot analysis with anti-p-P38 and anti-P38 antibodies of BV2 microglial cells treated with vehicle (1% DMSO) or selexipag (5 μM) for 45 min followed by PBS or LPS (200 ng/ml) for 45 min (n = 8-9/group). **(F-I)** Real-time PCR of proinflammatory mediator mRNA expression in BV2 microglial cells treated first with vehicle (1% DMSO) or SB203-580 (P38 inhibitor, 10 μM) for 30 min, then with vehicle (1% DMSO) or selexipag (5 μM) for 30 min, and finally with PBS or LPS (200 ng/ml) for 5.5 h (n = 8/group). *p < 0.05, **p < 0.01, ***p < 0.001.

To examine the underlying signaling pathway by which selexipag modulates LPS-induced proinflammatory responses, C57BL/6N mice were intraperitoneally (i.p.) administered selexipag (1 mg/kg) or vehicle (1% DMSO) and thereafter injected with PBS or LPS (10 mg/kg, i.p.) as described above. The hippocampus and cortex were subsequently dissected, and cAMP levels were measured by ELISA, which showed that selexipag treatment significantly increased cAMP levels in the hippocampus but not the cortex in LPS-treated C57B/L6 mice (**Figures 9A, B**).

We then investigated the effect of selexipag treatment on LPS-mediated downstream signaling. BV2 microglial cells were treated with vehicle (1% DMSO) or selexipag (5 μM) for 45 min followed by PBS or LPS (200 ng/ml) for 45 min. Western blotting with anti-p-P38 or anti-p-ERK antibodies revealed that selexipag treatment significantly reduced LPS-mediated P38 phosphorylation in BV2 microglial cells (**Figures 9C–E**) but not p-ERK levels (**Supplementary Figure 1**).

Next, we examined whether selexipag modulates LPS-induced proinflammatory responses via P38 signaling. BV2 microglial cells were treated with vehicle (1% DMSO) or the P38 inhibitor SB203-580 (10 μM) for 30 min, followed by vehicle (1% DMSO) or selexipag (5 μM) for 30 min. Finally, the cells were stimulated with PBS or LPS (200 ng/mL) for 5.5 h, and real-time PCR was performed. We found that selexipag or SB203–580 treatment significantly reduced LPS-induced IL-1β, IL-6, COX-2, and TNF-α mRNA levels in BV2 microglial cells, indicating effective inhibition of P38 activity (**Figures 9F–I**). Importantly, combined SB203-580, selexipag, and LPS (SB203-580 + selexipag + LPS) treatment did not further alter proinflammatory mediator mRNA levels in BV2 microglial cells compared to treatment with SB203–580 and LPS (SB203-580 + LPS) (**Figures 9F–I**). These findings indicate that the anti-inflammatory effects of selexipag against LPS stimulation are P38 dependent.

### Selexipag administration significantly reduced LPS-induced c-Jun phosphorylation in C57BL/6N mice and BV2 microglial cells

To further explore the downstream transcriptional signaling by which selexipag downregulates LPS-induced proinflammatory responses, C57BL/6N mice were administered selexipag (1 mg/kg, i.p.) or vehicle (1% DMSO) and thereafter injected with PBS or LPS (10 mg/kg, i.p.). Eight hours after the LPS/PBS injection, the hippocampus and cortex were subsequently dissected, and western blotting was performed with anti-p-c-Jun^s73^ and anti-c-Jun antibodies. We found that selexipag treatment significantly decreased LPS-induced p-c-Jun^S73^ expression in the hippocampus but not the cortex in C57BL/6N mice (**Supplementary Figure 3**). However, selexipag treatment did not affect LPS-induced p-STAT3^Y705^ and total STAT3 expression in the hippocampus and cortex (**Supplementary Figure 4**). These findings indicate that selexipag treatment downregulates c-Jun phosphorylation in LPS-treated C57BL/6N mice.

We further investigated whether selexipag downregulates LPS-induced proinflammatory responses via c-Jun by using a c-Jun antagonist *in vitro.* To test this, BV2 microglial cells were treated with vehicle (D.W.) or c-Jun peptide (a selective c-Jun antagonist, 100 μM) for 30 min followed by vehicle (1% DMSO) or selexipag (5 μM) for 30 min. Finally, the cells were stimulated with PBS or LPS (200 ng/mL) for 5.5 h, and Western blotting or real-time PCR was conducted. Because c-Jun peptide interferes with the JNK-c-Jun interaction, thereby reducing c-Jun phosphorylation (27), we first investigated the effect of c-Jun peptide treatment on inhibition of c-Jun phosphorylation by performing Western blotting with an anti-p-c-Jun^S73^ and anti-PCNA antibodies. We found that treatment with c-Jun peptide and LPS (c-Jun peptide + LPS) significantly reduced c-Jun phosphorylation compared with LPS treatment in BV2 microglial cells, indicating effective inhibition of c-Jun phosphorylation (**Supplementary Figures 5A, B**).

Next, we examined whether selexipag treatment downregulates LPS-mediated proinflammatory mediator levels via c-Jun signaling. For this experiment, BV2 microglial cells were treated as described above, and real-time PCR was conducted. We found that treatment with selexipag and LPS (selexipag + LPS) significantly reduced IL-1β, IL-6, COX-2, and TNF-α mRNA levels compared with LPS treatment (**Supplementary Figures 5C–F**). Surprisingly, c-Jun peptide + LPS significantly increased IL-1β and COX-2 mRNA levels but not IL-6 and TNF-α mRNA levels compared to LPS treatment in BV2 microglial cells (**Supplementary Figures 5C, E**). However, c-Jun peptide, selexipag and LPS (c-Jun peptide + selexipag + LPS) treatment further decreased IL-1β, IL-6, COX-2, and TNF-α mRNA levels compared to c-Jun peptide + LPS in BV2 microglial cells (**Supplementary Figures 5C–F**). These data indicate that selexipag treatment reduces LPS-induced proinflammatory mediator levels in a c-Jun-independent manner in BV2 microglial cells.

### Selexipag administration has a minor effect on pyroptosis in LPS-treated C57BL/6N mice

To determine whether selexipag treatment modulates pyroptosis *in vivo*, C57BL/6N mice were treated as described above, and the cortex and hippocampus were dissected for real-time PCR analysis. Selexipag treatment significantly suppressed LPS-induced GSDMD mRNA levels in the cortex and hippocampus in C57BL/6N mice (**Supplementary Figure 6A**). However, selexipag administration did not alter the mRNA levels of other pyroptosis-related genes, including NLRP6, CASPASE-1, ASC, IL-18, and HMGB1, in the cortex and hippocampus of LPS-treated C57BL/6N mice (**Supplementary Figures 6B–F**). These data suggest that selexipag selectively downregulates pyroptosis-associated GSDMD mRNA levels in LPS-treated C57BL/6N mice.

## Discussion

The present study is the first to demonstrate that selexipag, an IP receptor agonist, effectively attenuates LPS-mediated central neuroinflammation *in vitro* and *in vivo*. Specifically, selexipag treatment significantly reduced LPS-induced proinflammatory mediator mRNA and protein levels in BV2 microglial cells and/or primary microglial cells. In C57BL/6N mice, selexipag administration suppressed LPS-induced microglial/astroglial activation and subsequent proinflammatory mediator release in the brain. In addition, selexipag treatment markedly inhibited the LPS-induced upregulation of the mRNA levels of RA markers (CXCL10, SERPINA3N, GBP2, and CHI3L1) and a DAM marker (CD44) in the brain in C57BL/6N mice. Moreover, selexipag treatment suppressed LPS-induced NLRP3 inflammasome activation in BV2 microglial cells, primary microglial cells and/or C57BL/6N mice. We further validated that selexipag treatment alleviated LPS-mediated proinflammatory responses in an NLRP3-dependent manner in BV2 microglial cells. Finally, selexipag administration increased cAMP levels and suppressed P38 phosphorylation in LPS-treated C57BL/6N mice and BV2 microglial cells and exerted anti-inflammatory effects by suppressing P38 activity in BV2 microglial cells. Collectively, these findings provide compelling evidence that the IP receptor agonist selexipag may be a novel therapeutic agent for the prevention of neuroinflammation-associated diseases.

The IP receptor belongs to the Class A family of G-protein-coupled receptors (GPCRs) and mediates intracellular signaling by activating the Gs-adenylyl cyclase-cAMP pathway (11, 28). Increased cAMP levels suppress the production of proinflammatory mediators (TNF-α, IL-17, and IFN-γ) and promote the release of anti-inflammatory cytokine (IL-10) in dendritic cells, suggesting that targeting IP receptors might be a promising strategy to regulate inflammatory signaling in neurodegenerative diseases (13). However, this study is the first to examine whether the IP receptor agonist selexipag affects central neuroinflammation. We found that selexipag treatment significantly attenuated LPS-induced mRNA and protein levels of proinflammatory mediators (e.g., COX-2, IL-1β, IL-6, and TNF-α) in BV2 and primary microglial cells (**Figure 1**). Consistent with our observations, the IP receptor agonist ONO-1301 suppresses LPS-induced proinflammatory mediators (MCP-1, TNF-α, and iNOS) in macrophages isolated from the bone marrow of WT mice (29). Given that persistent microglial activation exacerbates proinflammatory mediator release, thereby contributing to neuronal damage and further pathoprogression of inflammation-associated diseases, pharmacological agonism of the IP receptor could be an effective strategy for suppressing microglial and astroglial proinflammation (21, 30).

The IP receptor is involved in both peripheral and central inflammatory responses; therefore, modulation of the IP receptor regulates inflammation-associated pathologies *in vivo*. For example, the IP receptor agonist beraprost exhibits antioxidative effects in a mouse model of ischemia, including reductions in microglial/macrophage infiltration, neutrophil accumulation, and lipid peroxidation (31, 32). Furthermore, The IP receptor agonist selexipag has also been reported to attenuate LPS-induced pulmonary inflammation in a mouse model of acute respiratory distress syndrome by protecting endothelial integrity and enhancing barrier function (17). However, it is unclear whether selexipag modulates glial cell activation. In the present study, we found that selexipag treatment significantly attenuated LPS-induced microglial and astroglial activation in the cortex and hippocampus in C57BL/6N mice (**Figures 2**, **3**). Specifically, selexipag administration reduced Iba-1-positive microglial activation at 8 h after LPS injection in C57BL/6N (**Figures 2A–D**). However, in BV2 microglial cells, short-term (3 h) selexipag treatment did not affect LPS-induced proinflammatory mediator expression, whereas prolonged treatment (6 h and 24 h) significantly suppressed neuroinflammatory responses (**Figures 1B–G**). These results indicate that the anti-inflammatory effects of selexipag are delayed *in vitro*.

The inability of selexipag to block the initial onset of inflammatory gene induction *in vitro* raises the question of how selexipag treatment leads to a marked reduction in microglial numbers within 8 h after LPS administration *in vivo*. It is possible that this discrepancy reflects differences in experimental design and microenvironment between the *in vitro* and *in vivo* studies. First, the *in vitro* (BV2 microglial cell) experiments were performed under single-treatment conditions [0.5, 1.0, or 5 μM selexipag or vehicle (1% DMSO), once], whereas the *in vivo* study involved repeated administration [1 mg/kg selexipag or vehicle (1% DMSO), i.p., daily for 7 days]. Importantly, a recent study demonstrated that repeated activation of the IP receptor, an on-target of selexipag, can elevate intracellular cAMP levels and induce sustained PKA/CREB signaling, thereby depriming microglia and attenuating rapid proinflammatory responses (17). For example, repeated administration of papaverine (cAMP activator, 30 mg/kg, i.p., daily for 4 days) significantly reduced LPS-induced Iba-1-positive microglial activation in C57BL/6J mice (33). These findings suggest that repeated pharmacological stimulation of IP receptors leads to sustained cAMP signaling, which might be associated with early attenuation (within 8 h) of LPS-induced microglial activation *in vivo*. Second, the *in vitro* experiments were conducted in a BV2 microglial monoculture system, whereas microglia in *in vivo* experiments are embedded within a dynamic neuro-glial-vascular microenvironment. Under these conditions, continuous crosstalk with neurons, astrocytes, and the vasculature could influence microglial functional state, morphology, migration, and density. Thus, selexipag treatment may affect both intrinsic microglial responsiveness and extrinsic signals from neurons and astrocytes, leading to decreased microglial activation in the brain. Our results suggest that selexipag treatment attenuates early microglial activation *in vivo* by regulating cAMP signaling at the network (including neuro-glial-vascular) level, despite the delayed anti-inflammatory effects observed in isolated BV2 microglial cells.

The role of IP receptors in regulating pro- and anti-inflammatory mediator release in peripheral tissues is well established. For example, genetic depletion of the IP receptor in WT mice exacerbates LPS-induced increases in proinflammatory mediators, such as IL-6 and TNF-α, in lung homogenates (34). Moreover, IP receptor-deficient mice exhibit aggravated lung inflammation following allergen challenge, characterized by increased eosinophil infiltration, vascular leakage, and elevated levels of Th2 mediators, including IL-4 and IL-5 (35, 36). Importantly, administration of the IP receptor agonist selexipag suppresses the LPS-induced increases in IL-1β, IL-6, and TNF-α levels in the lung tissue of WT mice (17). However, the effects of selexipag on central proinflammatory responses *in vivo* have not been elucidated. The present examination of the effects of selexipag on neuroinflammation demonstrated that selexipag administration ameliorated LPS-induced increases in COX-2, IL-1β, and TNF-α levels in the brain in C57BL/6N mice (**Figures 4**, **5**). Although selexipag treatment attenuated LPS-evoked glial activation in both the cortex and hippocampus, inhibitory effects of selexipag on LPS-induced proinflammatory mediator upregulation were limited to the hippocampus. IP receptors are predominantly expressed in the brain stem and moderately expressed in the hippocampus, cerebral cortex, and striatum, raising the possibility that the region-specific anti-inflammatory effects of selexipag reflect a higher proportion of IP receptor-expressing cells in the hippocampus compared with the cortex (37). Future studies will evaluate the differential distribution and density of IP receptor-expressing cells in the cortex, hippocampus and other brain regions that regulate neuroinflammation in mice treated with selexipag.

IP receptors regulate pathogen-induced peripheral inflammation by modulating immune cell accumulation *in vivo*. Specifically, antigen-stimulated accumulation of eosinophils, neutrophils, and lymphocytes is significantly enhanced in IP receptor knockout mice compared to WT mice (36). In the CNS, microglia and astrocytes serve as resident immune effector cells and undergo phenotypic transformation into reactive states in response to pathogen-associated molecular patterns (e.g., LPS) or neurotoxic protein aggregates (38). However, the effects of IP receptor agonism on microglia- and astroglia-associated neuroinflammatory dynamics have not been investigated. This study is the first to demonstrate that the IP receptor agonist selexipag attenuates the LPS-induced expression of markers of RAs (CXCL10, SERPINA3N, GBP2, and CHI3L1) and DAMs (CD44) in C57BL/6N mice (**Figure 6**). However, selexipag treatment did not affect the expression of a marker of homeostatic microglia (P2RY12), suggesting a partial but selective modulatory effect on CNS immune reactivity (**Figure 6**). Together with prior evidence, our findings indicate that the IP receptor is involved in regulating peripheral and central immune cell activation upon pathogen stimulation. Therefore, pharmacological agonism of IP receptors represents a promising strategy for mitigating immune reactivity in inflammation-related diseases.

Pathogenic stimuli (e.g., LPS) activate the NLRP3 inflammasome, leading to the maturation of proinflammatory mediators, including IL-1β and IL-18, and contributing to the progression of various neurological disorders (39). Additionally, NLRP3 inflammasome activation increases ROS, which further exacerbate neuronal stress, synaptic dysfunction, and BBB disruption (40). Importantly, post-symptomatic inhibition of NLRP3 in AD models has been shown to reverse cognitive decline, reduce Aβ and tau pathologies, diminish glial activation, and decrease neuroinflammatory cytokine levels, highlighting NLRP3 as a promising therapeutic target (41). Interestingly, cAMP, a key downstream effector of IP receptor signaling, inhibits NLRP3 inflammasome activation in bone marrow-derived macrophages by directly binding to the nucleotide-binding domain of NLRP3 and promoting the activation of protein kinase A (42).However, the effects of the IP receptor agonist selexipag on NLRP3 inflammasome activation have not been elucidated. We found that selexipag treatment inhibited the LPS-induced expression of NLRP3 and its inflammasome components (CASPASE-1 and pro-IL-1β) in BV2 microglial cells, primary microglial cells and/or C57BL/6N mice (**Figures 7**, **8**). These findings suggest that enhanced cAMP signaling due to selexipag treatment might suppress NLRP3 inflammasome activation and thereby dampens excessive inflammatory responses. Based on the literature and our findings, a future study will investigate whether selexipag administration suppresses LPS-induced NLRP3 inflammasome activation and proinflammatory responses via a cAMP-dependent pathway.

IP receptors are expressed in neuronal cells but not in presynaptic or glial cells in the striatum of the brain (43), although glial expression of IP receptors in the dorsal root ganglia of rats has been reported (44). These contrasting observations raise the possibility that selexipag may exert its anti-inflammatory effects either directly via glial IP receptors or indirectly by modulating neuron–glia interactions. When we investigated whether selexipag downregulates LPS-mediated proinflammatory responses through the IP receptor, we found that IP receptor antagonist BAY 73–1449 and LPS (BAY 73-1449 + LPS) treatment reduced LPS-induced COX-2 mRNA levels compared with LPS treatment alone without affecting IL-1β mRNA levels, whereas BAY 73-1449, selexipag, and LPS (BAY 73-1449 + selexipag + LPS) treatment did not alter LPS-induced IL-1β expression compared with treatment with BAY 73–1449 and LPS (BAY 73-1449 + LPS) or treatment with selexipag and LPS (selexipag + LPS) (**Supplementary Figure 2**). These findings indicate that selexipag downregulates LPS-induced proinflammatory cytokine IL-1β mRNA levels via the IP receptor in BV2 microglial cells. Consistent with our findings, a recent study used the IP receptor antagonist RO-1138452 to demonstrate that the IP receptor agonist cicaprost treatment inhibits LPS- or TNF-α-induced proinflammatory mediator mRNA levels in an IP receptor-dependent manner using with IP receptor antagonist RO-1138452 in human monocyte-derived macrophages and dendritic cells (45). However, in contrast to these and our findings, a study using the IP receptor antagonist CAY10441 found that the IP receptor agonist iloprost significantly increases protein levels of the proinflammatory mediator COX-2 through the IP receptor in human aortic smooth muscle cells (46). These results suggest that IP receptor agonists (i.e., selexipag, cicaprost, and iloprost differentially modulate proinflammatory responses in a stimulator-specific, inflammatory mediator-selective, or cell type-dependent manner. A limitation of our findings is that we used a single pharmacological antagonist (BAY 73-1449) to evaluate the effects of IP receptor inhibition on proinflammatory mediator expression. Thus, future studies will investigate whether IP receptor blockade consistently and selectively modulates LPS-induced proinflammatory mediator expression using structurally distinct IP receptor antagonists (e.g., RO-1138452 and CAY10441). We will also evaluate whether IP receptor knockdown differentially modulates LPS-mediated neuroinflammatory responses to establish the IP receptor dependence of selexipag without potential off-target effects.

Activation of the IP receptor increases intracellular cAMP, a key second messenger that differentially regulates inflammatory signaling pathways, including P38 MAPK, depending on cell type and stimulus. Specifically, treatment with the cAMP analog 8-bromo-cAMP suppresses LPS-induced P38 phosphorylation and reduces pro-inflammatory IL-12p40 expression in murine macrophages (47), whereas induction of the cAMP-PKA signaling pathway by norepinephrine increases P38 MAPK phosphorylation in S49 T-cell lineage cells (48). Thus, cAMP can bidirectionally regulate P38 MAPK signaling. In the present study, selexipag treatment significantly increased cAMP levels and reduced P38/c-Jun activation in LPS-treated BV2 microglial cells and/or C57BL/6N mice (**Figure 9**; **Supplementary Figures 3**, **5**). Interestingly, we found that the anti-inflammatory effects of selexipag against LPS treatment were P38-dependent but c-Jun-independent in BV2 microglial cells (**Figure 9**; **Supplementary Figure 5**). These findings suggest that the selexipag-mediated reduction in p-c-Jun expression may reflect a downstream consequence of P38 inhibition rather than a direct mechanism by which selexipag suppresses LPS-induced neuroinflammatory responses. Another possibility is that the reduction of c-Jun signaling by selexipag treatment affects biological functions (e.g., learning and memory, AD pathology) other than neuroinflammatory responses *in vitro* and *in vivo*. Thus, we will determine the molecular mechanisms by which selexipag suppresses c-Jun signaling in future studies.

A limitation of the present study is that we did not investigate whether selexipag treatment modulates LPS-mediated cognitive or affective alterations in C57BL/6N mice. We and others previously reported that systemic LPS administration elicits memory deficits and depressive-like behaviors in WT mice (49, 50). Furthermore, LPS exposure has been implicated in exacerbating AD-related pathophysiology, including neurotoxicity and memory impairments, inflammation, Aβ expression, and tau pathology (51). Therefore, mouse models of LPS-induced neuroinflammation can provide pivotal translational insights into the pathogenesis of neuropsychiatric and neurodegenerative disorders, including depressive disorders, intellectual disability, and AD. Given the anti-inflammatory effects of selexipag on LPS-induced neuroinflammation observed in both *in vitro* and *in vivo* models in this study, future investigations should explore whether selexipag also attenuates LPS-induced behavioral impairments. Specifically, evaluating the impact of selexipag on learning/memory and affective behaviors may provide important insights into its therapeutic potential for mitigating neuroinflammation-associated cognitive and emotional deficits.

## Conclusion

This study is the first to investigate the effects of selexipag, an IP receptor agonist, on LPS-induced central neuroinflammation *in vitro* and *in vivo* and the underlying mechanisms of its effects. First, we demonstrated that selexipag treatment diminishes LPS-induced proinflammatory mediator levels in BV2 and/or primary microglial cells. Second, we revealed that selexipag treatment mitigates LPS-induced microglia/astrocyte activation/hypertrophy, proinflammatory mediator release, and glial neuroinflammatory dynamics in C57BL/6N mice. Third, we found that selexipag administration inhibits neuroinflammatory responses in an NLRP3-dependent manner in BV2 microglial cells, primary microglial cells, and/or C57BL/6N mice. Finally, we demonstrated that selexipag treatment increases cAMP levels and has anti-inflammatory effects against LPS through P38 activity *in vitro* and *in vivo*. Taken together, these findings suggest that the IP receptor agonist selexipag is a plausible therapeutic agent for targeting P38/NLRP3 to prevent the neuropathological progression of neuroinflammation-related diseases, including depressive disorder, intellectual disability, and AD.

## Data Availability

The original contributions presented in the study are included in the article/**Supplementary Material**. Further inquiries can be directed to the corresponding authors.
