# Supplementary material for: A selective agonist of the prostacyclin receptor alleviates microglial and astroglial neuroinflammatory responses through P38 and NLRP3
Source: Front Immunol. 2026 Mar 25;17:1664119. doi: 10.3389/fimmu.2026.1664119 (PMC13056861; doi:10.3389/fimmu.2026.1664119)
Supplement: Supplementary file 1 [file DataSheet1.docx]

**A selective agonist of the prostacyclin (IP) receptor alleviates microglial and astroglial neuroinflammatory responses through P38 and NLRP3**

**Hyun-ju Lee^a,b,e^ , Sora Kang^a,e^ , Yoo Joo Jeong^a,b,c,e^, Tae-Mi Jung^b^,** **Jeong-Woo Hwang^a, b^, Ji-Yeong Jang^a,b,c^, Chan-Hu Gu^b^, Seokjun Oh^b^, Jeong-Heon Song^b^, Minho Moon^d,*^, Hyang-Sook Hoe^a,b,c,*^**

^a^Department of Neural Development and Disease, Korea Brain Research Institute (KBRI), 61, Cheomdan-ro, Dong-gu, Daegu, Republic of Korea, 41068; ^b^AI-based Neurodevelopmental Diseases Digital Therapeutics Group, Korea Brain Research Institute (KBRI), 61, Cheomdan-ro, Dong-gu, Daegu, Republic of Korea, 41068; ^c^Department of Brain Sciences, Daegu Gyeongbuk Institute of Science & Technology, Daegu, Republic of Korea 42988. ^d^Research Institute for Dementia Science, Konyang University, Daejeon, the Republic of Korea, 35365.^e^These authors contributed equally to this work.

***Corresponding authors**

**Hyang-Sook Hoe**, Ph.D.: Department of Neural Development and Disease, Korea Brain Research Institute (KBRI), 61 Cheomdan-ro, Dong-gu, Daegu, Korea, 41068; AI-based Neurodevelopmental Diseases Digital Therapeutics Group, Korea Brain Research Institute (KBRI), 61, Cheomdan-ro, Dong-gu, Daegu, Republic of Korea, 4106; E-mail: [sookhoe72@kbri.re.kr](mailto:sookhoe72@kbri.re.kr)

**Minho Moon, Ph.D**.: Department of Biochemistry, College of Medicine, Konyang University, 158, Gwanjeodong-ro, Seo-gu, Daejeon 35365, Republic of Korea: Tel.: +82-42-600-8691 E-mail: hominmoon@konyang.ac.kr

**
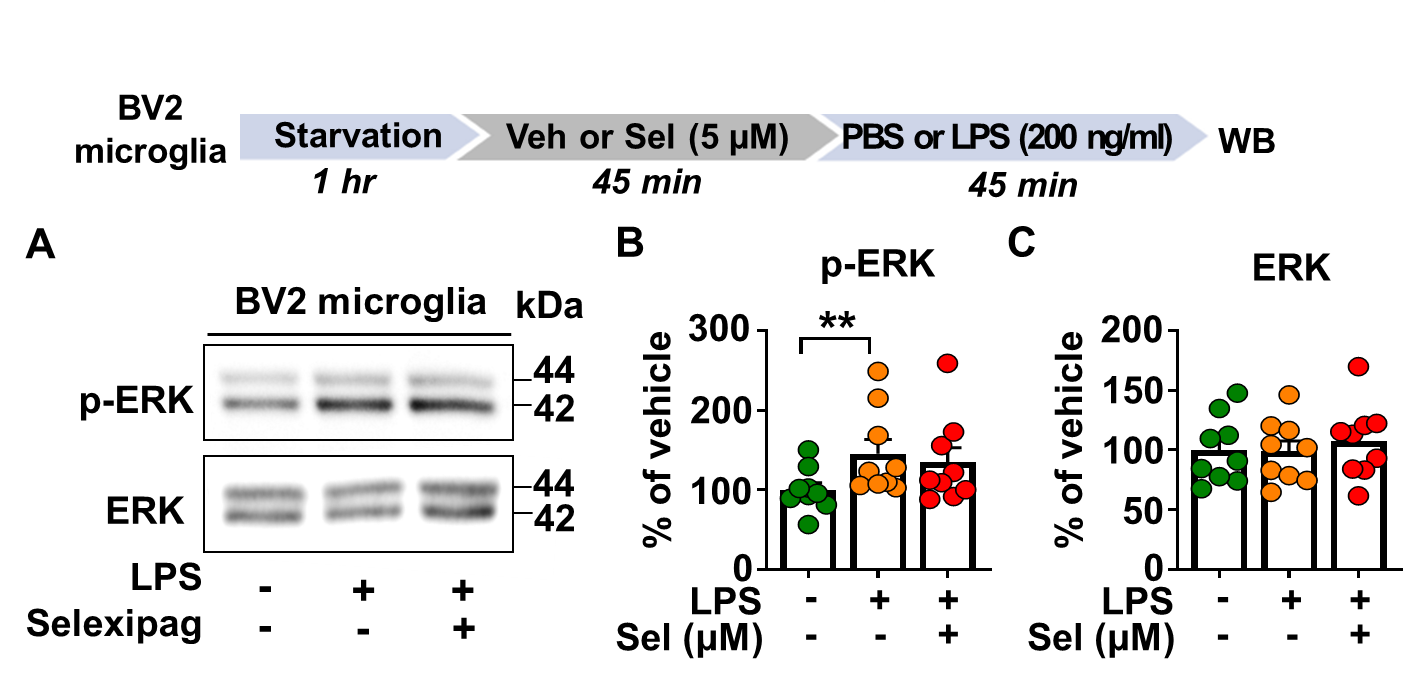
Supplementary Fig 1. Selexipag treatment does not alter LPS-induced p-ERK levels in BV2 microglial cells**. (**A**) BV2 microglial cells were treated with vehicle (1% DMSO) or selexipag (5 μM) for 45 min followed by PBS or LPS (200 ng/ml) for 45 min, and western blotting was conducted with anti-p-ERK or anti-ERK antibodies. (B-C) Quantification of data from A (n = 9/group). **p<0.01.


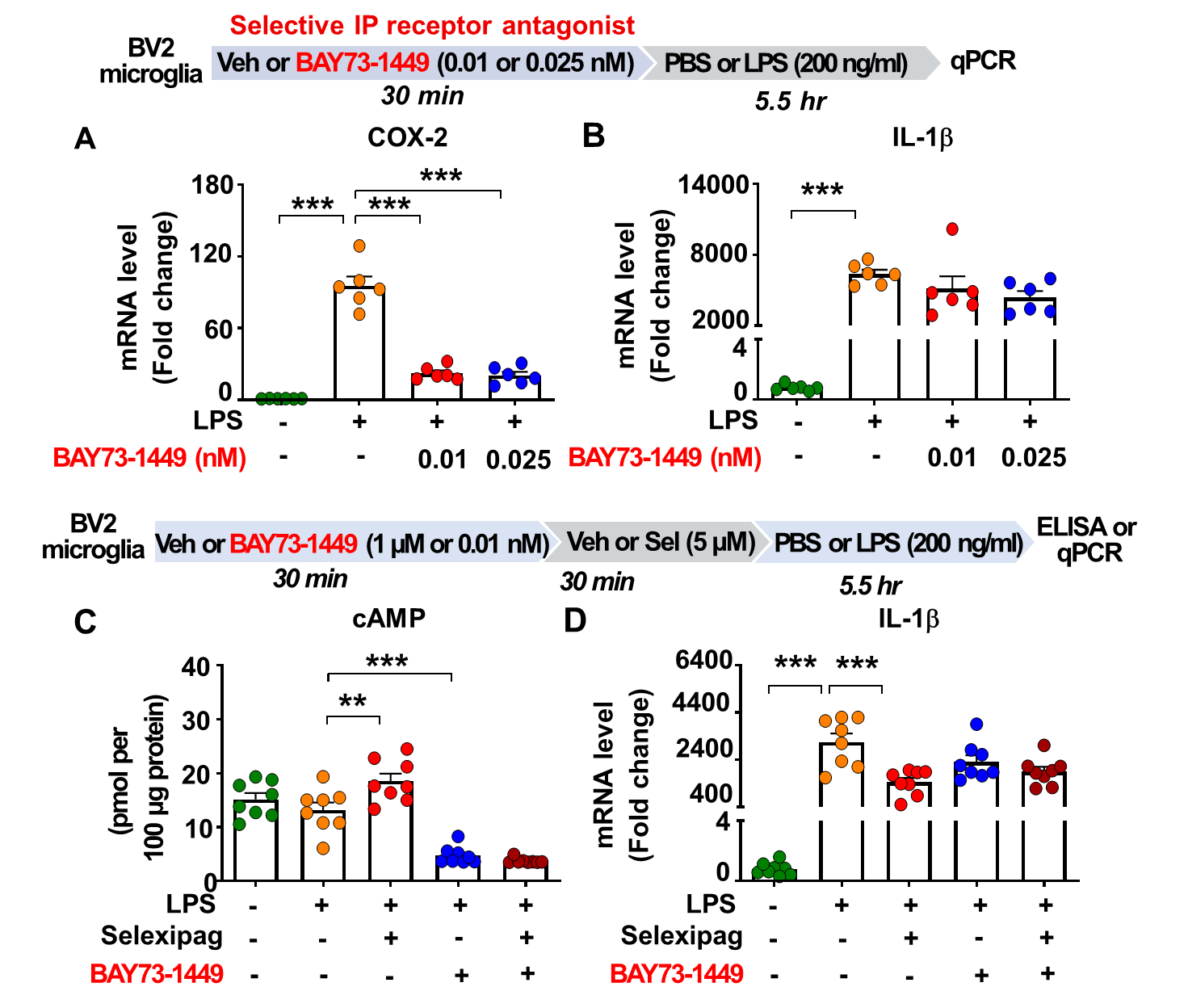
**Supplementary Fig 2. Selexipag administration downregulates LPS-induced IL-1β mRNA levels in an IP receptor-dependent manner in BV2 microglial cells.** (**A-B**) BV2 microglial cells were treated with vehicle (1% DMSO) or the IP receptor antagonist BAY73-1449 (0.01 or 0.025 nM) for 30 min followed by PBS or LPS (200 ng/ml) for 5.5 h, and real-time PCR was performed (n = 6/group). (**C**) BV2 microglial cells were treated with vehicle (1% DMSO) or BAY73-1449 (1 μM) for 30 min followed by vehicle (1% DMSO) or selexipag (5 μM) for 30 min. Next, the cells were stimulated with PBS or LPS (200 ng/ml) for 5.5 h, and intracellular cAMP levels were assessed by ELISA (n = 8/group). (**D**) Real-time PCR of IL-1β mRNA levels in BV2 microglial cells treated as described above (n = 8/group). **p<0.01, ***p < 0.001.

**
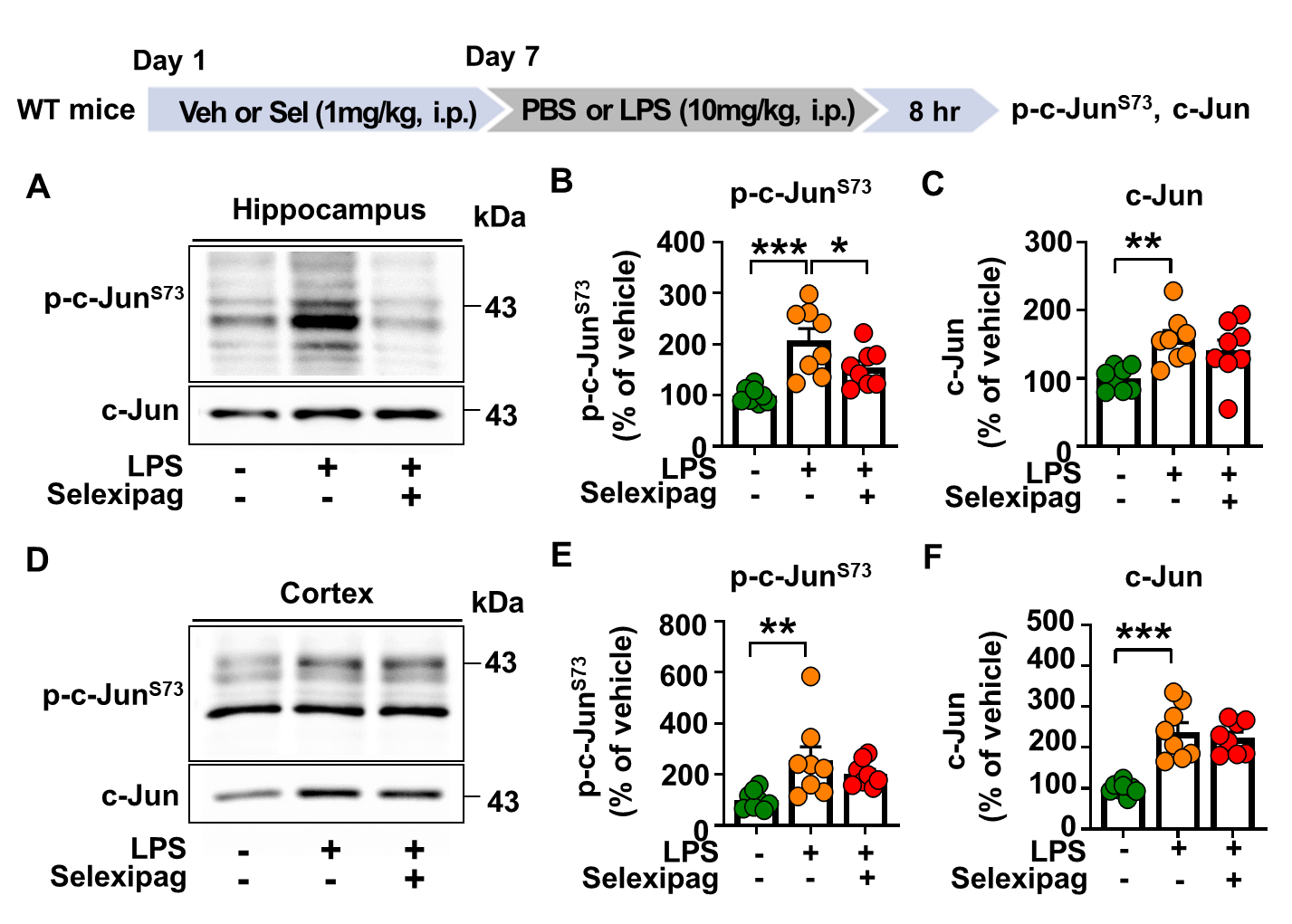
Supplementary Fig 3. Selexipag administration decreases c-Jun phosphorylation in LPS-injected C57BL/6N mice**. (**A-F)** Western blot analysis with anti-p-c-Jun^S73^ and anti-c-Jun antibodies in the hippocampus (A-C) and cortex (D-F) of C57BL/6N mice injected (i.p.) with vehicle (1% DMSO) or 1 mg/kg selexipag daily for 7 days, followed by 10 mg/kg LPS or PBS on day 7 (n= 8/group). *p < 0.05, **p<0.01, ***p < 0.001.

**
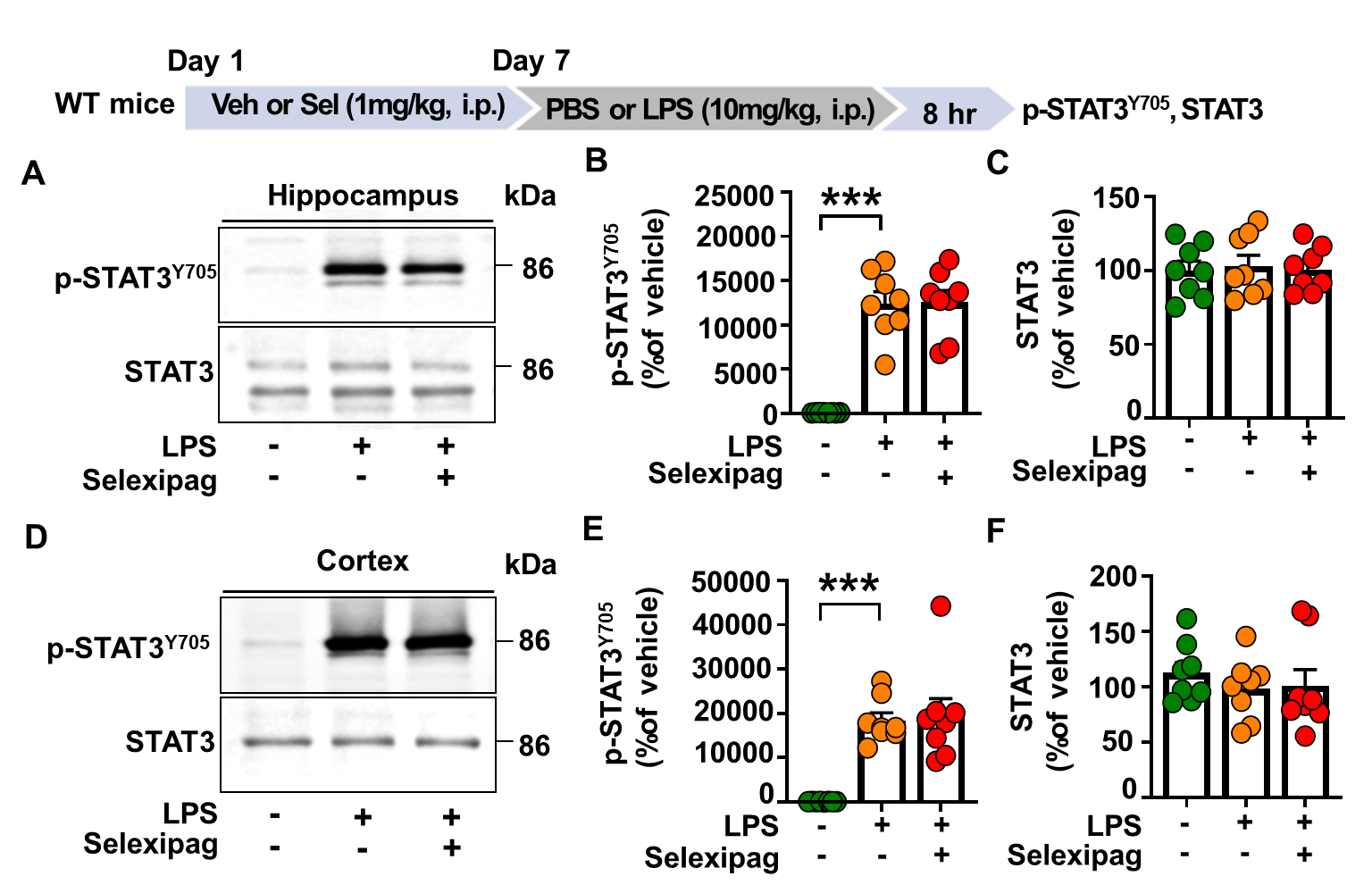
Supplementary Fig 4**. **Selexipag treatment does not alter LPS-mediated STAT3 phosphorylation in C57BL/6N mice.** (**A-F)** Western blot analysis with anti-p-STAT3^Y705^ and anti-STAT3 antibodies in the hippocampus (A-C) and cortex (D-F) of C57BL/6N mice injected (i.p.) with vehicle (1% DMSO) or 1 mg/kg selexipag daily for 7 days, followed by 10 mg/kg LPS or PBS on day 7 (n= 8/group). ***p < 0.001.

**
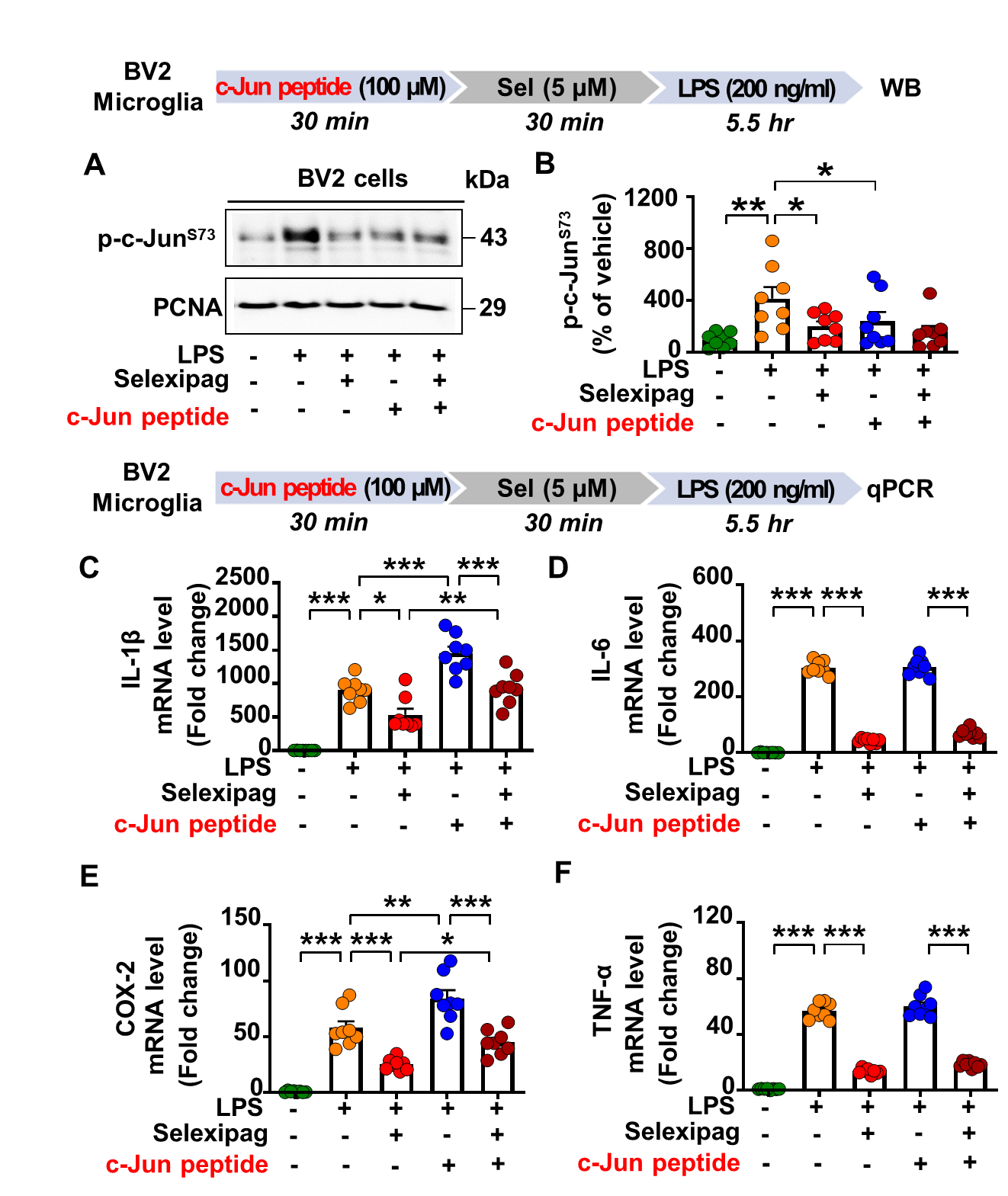
Supplementary Fig 5. Selexipag treatment downregulates LPS-mediated proinflammatory mediator levels in c-Jun-independent manner of BV2 microglial cells**. (**A-B**) Western blot analysis with anti-p-c-Jun^S73^ and anti-c-Jun antibodies in BV2 microglial cells treated first with vehicle (D.W.) or c-Jun peptide (c-Jun antagonist, 100 μM) for 30 min, then treated with vehicle (1% DMSO) or selexipag (5 μM) for 30 min, and finally treated with PBS or LPS (200 ng/ml) for 5.5 h (n = 8/group). (**C-F**) Real-time PCR of proinflammatory mediator mRNA expression in BV2 microglial cells treated as described above (n = 8/group). *p < 0.05, **p<0.01, ***p < 0.001.

**
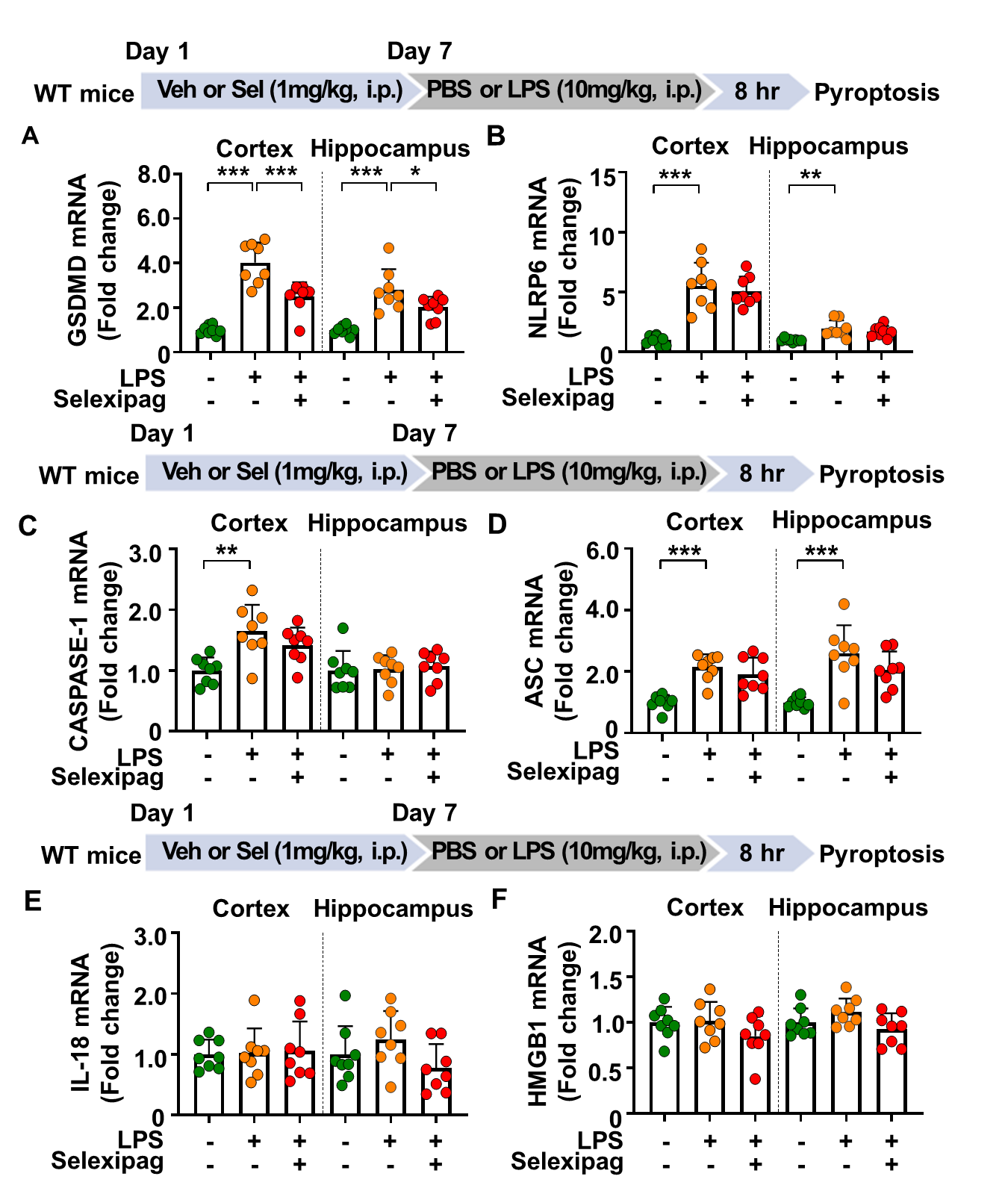
Supplementary Fig 6. Selexipag administration significantly suppresses pyroptosis-associated GSDMD mRNA levels in LPS-treated C57BL/6N mice.** (**A-F)** Real-time PCR of pyroptosis-related genes GSDMD, NLRP6, CASPASE-1, ASC, IL-18 and HMGB1 mRNA levels in the cortex and hippocampus of C57BL/6N mice injected (i.p.) with vehicle (1% DMSO) or 1 mg/kg selexipag daily for 7 days, followed by 10 mg/kg LPS or PBS on day 7 (n = 8/group). *p < 0.05, **p<0.01, ***p < 0.001.

**Supplementary Table 1. Antibodies used for IF in the present study**

| Primary antibodies | | | | | |
| --- | --- | --- | --- | --- | --- |
| **Antigen** | **Host species** | **Dilution** | **Manufacturer** | **Catalog no.** | **Application** |
| Iba-1 | Rabbit | 1:500 | Wako | 019-19741 | IF |
| GFAP | Chicken | 1:500 | Millipore | AB5541 | IF |
| COX-2 | Rabbit | 1:200 | Abcam | AB15191 | IF |
| IL-1β | Rabbit | 1:200 | Abcam | AB9722 | IF |
| TNF-α | Rabbit | 1:200 | Novus | NBP1-19532 | IF |
| **Secondary antibodies** | | | | | |
| **Antibody** | | **Dilution** | **Manufacturer** | **Catalog no.** | **Application** |
| Goat anti-rabbit IgG, Alexa Fluor 555 | | 1:200 | Invitrogen | A28180 | IF |
| Goat anti-chicken IgG, Alexa Fluor 488 | | 1:500 | Invitrogen | A11001 | IF |

**Supplementary Table 2. Primers sequences used for real-time qPCR in this study**

| Gene |  | Sequence |
| --- | --- | --- |
| COX-2 | Forward | 5’-CCA CTT CAA GGG AGT CTG GA-3’ |
|  | Reverse | 5’-AGT CAT CTG CTA CGG GAG GA-3’ |
| IL-1β | Forward | 5’-TTG ACG GAC CCC AAA AGA TG-3’ |
|  | Reverse | 5’-AGG ACA GCC CAG GTC AAA G-3’ |
| IL-6 | Forward | 5’-CCT GTC TTG GCC GAG GAC TA-3’ |
|  | Reverse | 5’-CCA CTT CAC AAG TCG GAG GC-3’ |
| TNF-α | Forward | 5’- TCC AGG CGG TGC CTA TGT-3’ |
|  | Reverse | 5’- GCC CCT GCC ACA AGC A-3’ |
| CXCL10 | Forward | 5’-GCC GTC ATT TTC TGC CTC A-3′ |
|  | Reverse | 5’-GCT TCC CTA TGG CCC TCA TT-3′ |
| SERPINA3N | Forward | 5’-CCC TGA GGA AGT GGA AGA AT-3′ |
|  | Reverse | 5’-CCT GAT GCC CAG CTT TGA AA-3′ |
| GBP2 | Forward | 5’-GGG GTC ACT GTC TGA CCA CT-3’ |
|  | Reverse | 5’-GGG AAA CCT GGG ATG AGA TT-3’ |
| CHI3L1 | Forward | 5’- CAA GGA ACT GAA TGC GGA AT-3’ |
|  | Reverse | 5’- CTG TGA TGG CCT GTG ATT TG-3’ |
| CD44 | Forward | 5’-ACT AGA TCC CTC CGT TTC ATC C-3′ |
|  | Reverse | 5’-GGT TAC ATT CAA ATC GAT CTG CTG-3′ |
| P2RY12 | Forward | 5’-GCT GCC TTG CTG AAG TCT CT-3′ |
|  | Reverse | 5’-AGG TGG TAT TGG CTG AGG TG-3′ |
| NLRP3 | Forward | 5’-TCC ACA ATT CTG ACC CAC AA-3′ |
|  | Reverse | 5’-ACC TCA CAG AGG GTC ACC AC-3′ |
| CASPASE-1 | Forward | 5’-GAT GGC ACA TTT CCA GGA CT-3’ |
|  | Reverse | 5’-CCC TCG GAG AAA GAT GTT GA-3’ |
| pro-IL-1β | Forward | 5’-TCT TTG AAG TTG ACG GAC CC-3’ |
|  | Reverse | 5’-TGA GTG ATA CTG CCT GCC TG-3’ |
| NLRP6 | Forward | 5’-CAG AAG GGC AAG CAA AAG AC-3’ |
|  | Reverse | 5’-ACA TTC AGC AAC ACG CTC AG-3’ |
| ASC | Forward | 5’-TGC AGA TGG ACG CCA TAG AT-3’ |
|  | Reverse | 5’- TGT GCT GGT CCA CAA AGT GT-3’ |
| GSDMD | Forward | 5’-GTT CCA GTG CCT CCA TGA AT-3’ |
|  | Reverse | 5’-ATA AAG CTC CAG GCA GCG TA-3’ |
| IL-18 | Forward | 5’-ACA ACT TTG GCC GAC TTC AC-3’ |
|  | Reverse | 5’-CAC AGC CAG TCC TCT TAC TTC A-3’ |
| HMGB1 | Forward | 5’-TCG GCC TTC TTC TTG TTC TG-3’ |
|  | Reverse | 5’-CAG CTT GGC AGC TTT CTT CT-3’ |
| GAPDH | Forward | 5’- TGT GTC CGT CGT GGA TCT GA-3’ |
|  | Reverse | 5’-CCT GCTTCA CCA CCT TCT TGA -3’ |

**Supplementary Table 3. Antibodies used for western blotting in this study**

| Primary antibodies | | | | | |
| --- | --- | --- | --- | --- | --- |
| **Antigen** | **Host species** | **Dilution** | **Manufacturer** | **Catalog no.** | **Application** |
| NLRP3 | Rabbit | 1:1000 | Cell Signaling | #1501 | WB |
| p-P38 | Rabbit | 1:1000 | Cell Signaling | #9211 | WB |
| P38 | Rabbit | 1:1000 | Cell Signaling | #9212 | WB |
| p-ERK | Rabbit | 1:1000 | Cell Signaling | #9101 | WB |
| ERK | Rabbit | 1:1000 | Cell Signaling | #9102 | WB |
| p-c-Jun^Ser73^ | Rabbit | 1:1000 | Cell Signaling | #9164 | WB |
| c-Jun | Rabbit | 1:1000 | Cell Signaling | #9165 | WB |
| p-STAT3^S705^ | Rabbit | 1:1000 | Cell Signaling | #9145 | WB |
| STAT3 | Mouse | 1:1000 | Santa Cruz | SC-8019 | WB |
| **Secondary antibodies** | | | | | |
| **Antibody** | | **Dilution** | **Manufacturer** | **Catalog no.** | **Application** |
| Goat anti-rabbit IgG, HRP conjugate | | 1:1000 | Enzo | ADI-SAB-300-J | WB |
| Goat anti-mouse IgG, HRP conjugate | | 1:1000 | Enzo | ADI-SAB-100-J | WB |

**Supplementary Table 4. Statistical analysis results** **for the *in vitro* or *in vivo* experiments in this study**

| **Figure 1A. BV2, MTT assay** |
| --- |
| \| Number of families \| 1 \|  \|  \|  \|  \|  \| \| --- \| --- \| --- \| --- \| --- \| --- \| --- \| \| Number of comparisons per family \| 45 \|  \|  \|  \|  \|  \| \| Alpha \| 0.05 \|  \|  \|  \|  \|  \| \|  \|  \|  \|  \|  \|  \|  \| \| Tukey's multiple comparisons test \| Mean Diff. \| 95.00% CI of diff. \| Below threshold? \| Summary \| Adjusted P Value \|  \| \| Column A vs. Column B \| -8.742 \| -19.26 to 1.779 \| No \| ns \| 0.1934 \| A-B \| \| Column A vs. Column C \| 8.333e-010 \| -10.52 to 10.52 \| No \| ns \| >0.9999 \| A-C \| \| Column A vs. Column D \| -6.640 \| -17.16 to 3.881 \| No \| ns \| 0.5742 \| A-D \| \| Column A vs. Column E \| 0.000 \| -10.52 to 10.52 \| No \| ns \| >0.9999 \| A-E \| \| Column A vs. Column F \| 1.966 \| -8.791 to 12.72 \| No \| ns \| 0.9999 \| A-F \| \| Column A vs. Column G \| 9.167e-009 \| -10.52 to 10.52 \| No \| ns \| >0.9999 \| A-G \| \| Column A vs. Column H \| 5.878 \| -4.643 to 16.40 \| No \| ns \| 0.7308 \| A-H \| \| Column A vs. Column I \| -4.167e-009 \| -10.52 to 10.52 \| No \| ns \| >0.9999 \| A-I \| \| Column A vs. Column J \| -5.795 \| -16.32 to 4.726 \| No \| ns \| 0.7466 \| A-J \| \| Column B vs. Column C \| 8.742 \| -1.779 to 19.26 \| No \| ns \| 0.1934 \| B-C \| \| Column B vs. Column D \| 2.102 \| -8.419 to 12.62 \| No \| ns \| 0.9997 \| B-D \| \| Column B vs. Column E \| 8.742 \| -1.779 to 19.26 \| No \| ns \| 0.1934 \| B-E \| \| Column B vs. Column F \| 10.71 \| -0.04958 to 21.46 \| No \| ns \| 0.0521 \| B-F \| \| Column B vs. Column G \| 8.742 \| -1.779 to 19.26 \| No \| ns \| 0.1934 \| B-G \| \| Column B vs. Column H \| 14.62 \| 4.099 to 25.14 \| Yes \| *** \| 0.0007 \| B-H \| \| Column B vs. Column I \| 8.742 \| -1.779 to 19.26 \| No \| ns \| 0.1934 \| B-I \| \| Column B vs. Column J \| 2.947 \| -7.574 to 13.47 \| No \| ns \| 0.9960 \| B-J \| \| Column C vs. Column D \| -6.640 \| -17.16 to 3.881 \| No \| ns \| 0.5742 \| C-D \| \| Column C vs. Column E \| -8.333e-010 \| -10.52 to 10.52 \| No \| ns \| >0.9999 \| C-E \| \| Column C vs. Column F \| 1.966 \| -8.791 to 12.72 \| No \| ns \| 0.9999 \| C-F \| \| Column C vs. Column G \| 8.333e-009 \| -10.52 to 10.52 \| No \| ns \| >0.9999 \| C-G \| \| Column C vs. Column H \| 5.878 \| -4.643 to 16.40 \| No \| ns \| 0.7308 \| C-H \| \| Column C vs. Column I \| -5.000e-009 \| -10.52 to 10.52 \| No \| ns \| >0.9999 \| C-I \| \| Column C vs. Column J \| -5.795 \| -16.32 to 4.726 \| No \| ns \| 0.7466 \| C-J \| \| Column D vs. Column E \| 6.640 \| -3.881 to 17.16 \| No \| ns \| 0.5742 \| D-E \| \| Column D vs. Column F \| 8.605 \| -2.152 to 19.36 \| No \| ns \| 0.2379 \| D-F \| \| Column D vs. Column G \| 6.640 \| -3.881 to 17.16 \| No \| ns \| 0.5742 \| D-G \| \| Column D vs. Column H \| 12.52 \| 1.997 to 23.04 \| Yes \| ** \| 0.0075 \| D-H \| \| Column D vs. Column I \| 6.640 \| -3.881 to 17.16 \| No \| ns \| 0.5742 \| D-I \| \| Column D vs. Column J \| 0.8447 \| -9.676 to 11.37 \| No \| ns \| >0.9999 \| D-J \| \| Column E vs. Column F \| 1.966 \| -8.791 to 12.72 \| No \| ns \| 0.9999 \| E-F \| \| Column E vs. Column G \| 9.167e-009 \| -10.52 to 10.52 \| No \| ns \| >0.9999 \| E-G \| \| Column E vs. Column H \| 5.878 \| -4.643 to 16.40 \| No \| ns \| 0.7308 \| E-H \| \| Column E vs. Column I \| -4.167e-009 \| -10.52 to 10.52 \| No \| ns \| >0.9999 \| E-I \| \| Column E vs. Column J \| -5.795 \| -16.32 to 4.726 \| No \| ns \| 0.7466 \| E-J \| \| Column F vs. Column G \| -1.966 \| -12.72 to 8.791 \| No \| ns \| 0.9999 \| F-G \| \| Column F vs. Column H \| 3.912 \| -6.845 to 14.67 \| No \| ns \| 0.9747 \| F-H \| \| Column F vs. Column I \| -1.966 \| -12.72 to 8.791 \| No \| ns \| 0.9999 \| F-I \| \| Column F vs. Column J \| -7.761 \| -18.52 to 2.996 \| No \| ns \| 0.3784 \| F-J \| \| Column G vs. Column H \| 5.878 \| -4.643 to 16.40 \| No \| ns \| 0.7308 \| G-H \| \| Column G vs. Column I \| -1.333e-008 \| -10.52 to 10.52 \| No \| ns \| >0.9999 \| G-I \| \| Column G vs. Column J \| -5.795 \| -16.32 to 4.726 \| No \| ns \| 0.7466 \| G-J \| \| Column H vs. Column I \| -5.878 \| -16.40 to 4.643 \| No \| ns \| 0.7308 \| H-I \| \| Column H vs. Column J \| -11.67 \| -22.19 to -1.152 \| Yes \| * \| 0.0175 \| H-J \| \| Column I vs. Column J \| -5.795 \| -16.32 to 4.726 \| No \| ns \| 0.7466 \| I-J \| |
| **Figure 1B. BV2 (0.5 μM Selexipag, 6 hr), IL-1β mRNA level** |
| \| Number of families \| 1 \|  \|  \|  \|  \|  \| \| --- \| --- \| --- \| --- \| --- \| --- \| --- \| \| Number of comparisons per family \| 3 \|  \|  \|  \|  \|  \| \| Alpha \| 0.05 \|  \|  \|  \|  \|  \| \|  \|  \|  \|  \|  \|  \|  \| \| Tukey's multiple comparisons test \| Mean Diff. \| 95.00% CI of diff. \| Below threshold? \| Summary \| Adjusted P Value \|  \| \| - - vs. + - \| -944.8 \| -1053 to -836.8 \| Yes \| **** \| <0.0001 \| A-B \| \| - - vs. + + \| -547.5 \| -655.5 to -439.6 \| Yes \| **** \| <0.0001 \| A-C \| \| + - vs. + + \| 397.2 \| 289.3 to 505.2 \| Yes \| **** \| <0.0001 \| B-C \| |
| **Figure 1B. BV2 (0.5 μM Selexipag, 6 hr), IL-6 mRNA level** |
| \| Number of families \| 1 \|  \|  \|  \|  \|  \| \| --- \| --- \| --- \| --- \| --- \| --- \| --- \| \| Number of comparisons per family \| 3 \|  \|  \|  \|  \|  \| \| Alpha \| 0.05 \|  \|  \|  \|  \|  \| \|  \|  \|  \|  \|  \|  \|  \| \| Tukey's multiple comparisons test \| Mean Diff. \| 95.00% CI of diff. \| Below threshold? \| Summary \| Adjusted P Value \|  \| \| - - vs. + - \| -18.65 \| -27.97 to -9.342 \| Yes \| *** \| 0.0002 \| D-E \| \| - - vs. + + \| -8.457 \| -17.77 to 0.8550 \| No \| ns \| 0.0795 \| D-F \| \| + - vs. + + \| 10.20 \| 0.8851 to 19.51 \| Yes \| * \| 0.0302 \| E-F \| |
| **Figure 1B. BV2 (0.5 μM Selexipag, 6 hr), COX-2 mRNA level** |
| \| Number of families \| 1 \|  \|  \|  \|  \|  \| \| --- \| --- \| --- \| --- \| --- \| --- \| --- \| \| Number of comparisons per family \| 3 \|  \|  \|  \|  \|  \| \| Alpha \| 0.05 \|  \|  \|  \|  \|  \| \|  \|  \|  \|  \|  \|  \|  \| \| Tukey's multiple comparisons test \| Mean Diff. \| 95.00% CI of diff. \| Below threshold? \| Summary \| Adjusted P Value \|  \| \| - - vs. + - \| -92.12 \| -160.6 to -23.65 \| Yes \| ** \| 0.0076 \| G-H \| \| - - vs. + + \| -14.99 \| -85.87 to 55.89 \| No \| ns \| 0.8551 \| G-I \| \| + - vs. + + \| 77.13 \| 6.253 to 148.0 \| Yes \| * \| 0.0315 \| H-I \| |
| **Figure 1B. BV2 (0.5 μM Selexipag, 6 hr), TNF-α mRNA level** |
| \| Number of families \| 1 \|  \|  \|  \|  \|  \| \| --- \| --- \| --- \| --- \| --- \| --- \| --- \| \| Number of comparisons per family \| 3 \|  \|  \|  \|  \|  \| \| Alpha \| 0.05 \|  \|  \|  \|  \|  \| \|  \|  \|  \|  \|  \|  \|  \| \| Tukey's multiple comparisons test \| Mean Diff. \| 95.00% CI of diff. \| Below threshold? \| Summary \| Adjusted P Value \|  \| \| - - vs. + - \| -35.47 \| -51.19 to -19.76 \| Yes \| **** \| <0.0001 \| J-K \| \| - - vs. + + \| -13.19 \| -28.90 to 2.529 \| No \| ns \| 0.1109 \| J-L \| \| + - vs. + + \| 22.29 \| 6.569 to 38.00 \| Yes \| ** \| 0.0049 \| K-L \| |
| **Figure 1C. BV2 (1.0 μM Selexipag, 6 hr), IL-1β mRNA level** |
| \| Number of families \| 1 \|  \|  \|  \|  \|  \| \| --- \| --- \| --- \| --- \| --- \| --- \| --- \| \| Number of comparisons per family \| 3 \|  \|  \|  \|  \|  \| \| Alpha \| 0.05 \|  \|  \|  \|  \|  \| \|  \|  \|  \|  \|  \|  \|  \| \| Tukey's multiple comparisons test \| Mean Diff. \| 95.00% CI of diff. \| Below threshold? \| Summary \| Adjusted P Value \|  \| \| - - vs. + - \| -387.9 \| -456.3 to -319.5 \| Yes \| **** \| <0.0001 \| A-B \| \| - - vs. + + \| -109.6 \| -178.0 to -41.22 \| Yes \| ** \| 0.0040 \| A-C \| \| + - vs. + + \| 278.3 \| 209.9 to 346.7 \| Yes \| **** \| <0.0001 \| B-C \| |
| **Figure 1C. BV2 (1.0 μM Selexipag, 6 hr), IL-6 mRNA level** |
| \| Number of families \| 1 \|  \|  \|  \|  \|  \| \| --- \| --- \| --- \| --- \| --- \| --- \| --- \| \| Number of comparisons per family \| 3 \|  \|  \|  \|  \|  \| \| Alpha \| 0.05 \|  \|  \|  \|  \|  \| \|  \|  \|  \|  \|  \|  \|  \| \| Tukey's multiple comparisons test \| Mean Diff. \| 95.00% CI of diff. \| Below threshold? \| Summary \| Adjusted P Value \|  \| \| - - vs. + - \| -218.9 \| -272.8 to -165.1 \| Yes \| **** \| <0.0001 \| D-E \| \| - - vs. + + \| -66.73 \| -120.6 to -12.85 \| Yes \| * \| 0.0178 \| D-F \| \| + - vs. + + \| 152.2 \| 98.33 to 206.1 \| Yes \| **** \| <0.0001 \| E-F \| |
| **Figure 1C. BV2 (1.0 μM Selexipag, 6 hr), COX-2 mRNA level** |
| \| Number of families \| 1 \|  \|  \|  \|  \|  \| \| --- \| --- \| --- \| --- \| --- \| --- \| --- \| \| Number of comparisons per family \| 3 \|  \|  \|  \|  \|  \| \| Alpha \| 0.05 \|  \|  \|  \|  \|  \| \|  \|  \|  \|  \|  \|  \|  \| \| Tukey's multiple comparisons test \| Mean Diff. \| 95.00% CI of diff. \| Below threshold? \| Summary \| Adjusted P Value \|  \| \| - - vs. + - \| -80.77 \| -109.0 to -52.55 \| Yes \| **** \| <0.0001 \| G-H \| \| - - vs. + + \| -37.70 \| -65.92 to -9.471 \| Yes \| * \| 0.0118 \| G-I \| \| + - vs. + + \| 43.08 \| 14.85 to 71.30 \| Yes \| ** \| 0.0054 \| H-I \| |
| **Figure 1C. BV2 (1.0 μM Selexipag, 6 hr), TNF-α mRNA level** |
| \| Number of families \| 1 \|  \|  \|  \|  \|  \| \| --- \| --- \| --- \| --- \| --- \| --- \| --- \| \| Number of comparisons per family \| 3 \|  \|  \|  \|  \|  \| \| Alpha \| 0.05 \|  \|  \|  \|  \|  \| \|  \|  \|  \|  \|  \|  \|  \| \| Tukey's multiple comparisons test \| Mean Diff. \| 95.00% CI of diff. \| Below threshold? \| Summary \| Adjusted P Value \|  \| \| - - vs. + - \| -101.2 \| -119.9 to -82.61 \| Yes \| **** \| <0.0001 \| J-K \| \| - - vs. + + \| -27.45 \| -46.07 to -8.829 \| Yes \| ** \| 0.0066 \| J-L \| \| + - vs. + + \| 73.78 \| 55.17 to 92.40 \| Yes \| **** \| <0.0001 \| K-L \| |
| **Figure 1D. BV2 (5 μM Selexipag, 6 hr), IL-1β mRNA level** |
| \| Number of families \| 1 \|  \|  \|  \|  \|  \| \| --- \| --- \| --- \| --- \| --- \| --- \| --- \| \| Number of comparisons per family \| 3 \|  \|  \|  \|  \|  \| \| Alpha \| 0.05 \|  \|  \|  \|  \|  \| \|  \|  \|  \|  \|  \|  \|  \| \| Tukey's multiple comparisons test \| Mean Diff. \| 95.00% CI of diff. \| Below threshold? \| Summary \| Adjusted P Value \|  \| \| - - vs. + - \| -395.5 \| -494.2 to -296.8 \| Yes \| **** \| <0.0001 \| A-B \| \| - - vs. + + \| -99.55 \| -198.2 to -0.8748 \| Yes \| * \| 0.0481 \| A-C \| \| + - vs. + + \| 295.9 \| 197.3 to 394.6 \| Yes \| **** \| <0.0001 \| B-C \| |
| **Figure 1D. BV2 (5 μM Selexipag, 6 hr), IL-6 mRNA level** |
| \| Number of families \| 1 \|  \|  \|  \|  \|  \| \| --- \| --- \| --- \| --- \| --- \| --- \| --- \| \| Number of comparisons per family \| 3 \|  \|  \|  \|  \|  \| \| Alpha \| 0.05 \|  \|  \|  \|  \|  \| \|  \|  \|  \|  \|  \|  \|  \| \| Tukey's multiple comparisons test \| Mean Diff. \| 95.00% CI of diff. \| Below threshold? \| Summary \| Adjusted P Value \|  \| \| - - vs. + - \| -616.1 \| -854.8 to -377.4 \| Yes \| *** \| 0.0001 \| D-E \| \| - - vs. + + \| -81.80 \| -320.5 to 156.9 \| No \| ns \| 0.6203 \| D-F \| \| + - vs. + + \| 534.3 \| 295.6 to 773.0 \| Yes \| *** \| 0.0004 \| E-F \| |
| **Figure 1D. BV2 (5 μM Selexipag, 6 hr), COX-2 mRNA level** |
| \| Number of families \| 1 \|  \|  \|  \|  \|  \| \| --- \| --- \| --- \| --- \| --- \| --- \| --- \| \| Number of comparisons per family \| 3 \|  \|  \|  \|  \|  \| \| Alpha \| 0.05 \|  \|  \|  \|  \|  \| \|  \|  \|  \|  \|  \|  \|  \| \| Tukey's multiple comparisons test \| Mean Diff. \| 95.00% CI of diff. \| Below threshold? \| Summary \| Adjusted P Value \|  \| \| - - vs. + - \| -66.16 \| -92.99 to -39.34 \| Yes \| *** \| 0.0002 \| G-H \| \| - - vs. + + \| -30.22 \| -57.05 to -3.396 \| Yes \| * \| 0.0288 \| G-I \| \| + - vs. + + \| 35.94 \| 9.117 to 62.77 \| Yes \| * \| 0.0116 \| H-I \| |
| **Figure 1D. BV2 (5 μM Selexipag, 6 hr), TNF-α mRNA level** |
| \| Number of families \| 1 \|  \|  \|  \|  \|  \| \| --- \| --- \| --- \| --- \| --- \| --- \| --- \| \| Number of comparisons per family \| 3 \|  \|  \|  \|  \|  \| \| Alpha \| 0.05 \|  \|  \|  \|  \|  \| \|  \|  \|  \|  \|  \|  \|  \| \| Tukey's multiple comparisons test \| Mean Diff. \| 95.00% CI of diff. \| Below threshold? \| Summary \| Adjusted P Value \|  \| \| - - vs. + - \| -105.0 \| -131.2 to -78.90 \| Yes \| **** \| <0.0001 \| J-K \| \| - - vs. + + \| -26.78 \| -52.92 to -0.6477 \| Yes \| * \| 0.0449 \| J-L \| \| + - vs. + + \| 78.25 \| 52.11 to 104.4 \| Yes \| **** \| <0.0001 \| K-L \| |
| **Figure 1E. BV2 (0.5 μM Selexipag, 6 hr), IL-1β protein level** |
| \| Number of families \| 1 \|  \|  \|  \|  \|  \| \| --- \| --- \| --- \| --- \| --- \| --- \| --- \| \| Number of comparisons per family \| 3 \|  \|  \|  \|  \|  \| \| Alpha \| 0.05 \|  \|  \|  \|  \|  \| \|  \|  \|  \|  \|  \|  \|  \| \| Tukey's multiple comparisons test \| Mean Diff. \| 95.00% CI of diff. \| Below threshold? \| Summary \| Adjusted P Value \|  \| \| - - vs. + - \| -917.8 \| -1176 to -659.4 \| Yes \| **** \| <0.0001 \| A-B \| \| - - vs. + + \| -565.8 \| -824.2 to -307.4 \| Yes \| **** \| <0.0001 \| A-C \| \| + - vs. + + \| 352.0 \| 93.58 to 610.4 \| Yes \| ** \| 0.0068 \| B-C \| |
| **Figure 1E. BV2 (0.5 μM Selexipag, 6 hr), IL-6 protein level** |
| \| Number of families \| 1 \|  \|  \|  \|  \|  \| \| --- \| --- \| --- \| --- \| --- \| --- \| --- \| \| Number of comparisons per family \| 3 \|  \|  \|  \|  \|  \| \| Alpha \| 0.05 \|  \|  \|  \|  \|  \| \|  \|  \|  \|  \|  \|  \|  \| \| Tukey's multiple comparisons test \| Mean Diff. \| 95.00% CI of diff. \| Below threshold? \| Summary \| Adjusted P Value \|  \| \| - - vs. + - \| -623.6 \| -786.3 to -460.8 \| Yes \| **** \| <0.0001 \| D-E \| \| - - vs. + + \| -125.4 \| -288.1 to 37.37 \| No \| ns \| 0.1519 \| D-F \| \| + - vs. + + \| 498.2 \| 335.5 to 660.9 \| Yes \| **** \| <0.0001 \| E-F \| |
| **Figure 1E. BV2 (0.5 μM Selexipag, 6 hr), COX-2 protein level** |
| \| Number of families \| 1 \|  \|  \|  \|  \|  \| \| --- \| --- \| --- \| --- \| --- \| --- \| --- \| \| Number of comparisons per family \| 3 \|  \|  \|  \|  \|  \| \| Alpha \| 0.05 \|  \|  \|  \|  \|  \| \|  \|  \|  \|  \|  \|  \|  \| \| Tukey's multiple comparisons test \| Mean Diff. \| 95.00% CI of diff. \| Below threshold? \| Summary \| Adjusted P Value \|  \| \| - - vs. + - \| -142.8 \| -164.8 to -120.7 \| Yes \| **** \| <0.0001 \| G-H \| \| - - vs. + + \| 15.43 \| -6.624 to 37.49 \| No \| ns \| 0.2061 \| G-I \| \| + - vs. + + \| 158.2 \| 136.2 to 180.3 \| Yes \| **** \| <0.0001 \| H-I \| |
| **Figure 1E. BV2 (0.5 μM Selexipag, 6 hr), TNF-α protein level** |
| \| Number of families \| 1 \|  \|  \|  \|  \|  \| \| --- \| --- \| --- \| --- \| --- \| --- \| --- \| \| Number of comparisons per family \| 3 \|  \|  \|  \|  \|  \| \| Alpha \| 0.05 \|  \|  \|  \|  \|  \| \|  \|  \|  \|  \|  \|  \|  \| \| Tukey's multiple comparisons test \| Mean Diff. \| 95.00% CI of diff. \| Below threshold? \| Summary \| Adjusted P Value \|  \| \| - - vs. + - \| -645.3 \| -732.4 to -558.1 \| Yes \| **** \| <0.0001 \| J-K \| \| - - vs. + + \| -485.9 \| -573.0 to -398.7 \| Yes \| **** \| <0.0001 \| J-L \| \| + - vs. + + \| 159.4 \| 72.23 to 246.5 \| Yes \| *** \| 0.0004 \| K-L \| |
| **Figure 1F. BV2 (0.5 μM Selexipag, 3 hr), IL-1β mRNA level** |
| \| Number of families \| 1 \|  \|  \|  \|  \|  \| \| --- \| --- \| --- \| --- \| --- \| --- \| --- \| \| Number of comparisons per family \| 3 \|  \|  \|  \|  \|  \| \| Alpha \| 0.05 \|  \|  \|  \|  \|  \| \|  \|  \|  \|  \|  \|  \|  \| \| Tukey's multiple comparisons test \| Mean Diff. \| 95.00% CI of diff. \| Below threshold? \| Summary \| Adjusted P Value \|  \| \| - - vs. + - \| -1112 \| -1476 to -748.0 \| Yes \| **** \| <0.0001 \| A-B \| \| - - vs. + + \| -960.2 \| -1324 to -596.3 \| Yes \| **** \| <0.0001 \| A-C \| \| + - vs. + + \| 151.8 \| -212.1 to 515.7 \| No \| ns \| 0.5538 \| B-C \| |
| **Figure 1F. BV2 (0.5 μM Selexipag, 3 hr), IL-6 mRNA level** |
| \| Number of families \| 1 \|  \|  \|  \|  \|  \| \| --- \| --- \| --- \| --- \| --- \| --- \| --- \| \| Number of comparisons per family \| 3 \|  \|  \|  \|  \|  \| \| Alpha \| 0.05 \|  \|  \|  \|  \|  \| \|  \|  \|  \|  \|  \|  \|  \| \| Tukey's multiple comparisons test \| Mean Diff. \| 95.00% CI of diff. \| Below threshold? \| Summary \| Adjusted P Value \|  \| \| - - vs. + - \| -213.3 \| -279.5 to -147.2 \| Yes \| **** \| <0.0001 \| D-E \| \| - - vs. + + \| -170.9 \| -237.0 to -104.7 \| Yes \| **** \| <0.0001 \| D-F \| \| + - vs. + + \| 42.49 \| -23.66 to 108.6 \| No \| ns \| 0.2599 \| E-F \| |
| **Figure 1F. BV2 (0.5 μM Selexipag, 3 hr), COX-2 mRNA level** |
| \| Number of families \| 1 \|  \|  \|  \|  \|  \| \| --- \| --- \| --- \| --- \| --- \| --- \| --- \| \| Number of comparisons per family \| 3 \|  \|  \|  \|  \|  \| \| Alpha \| 0.05 \|  \|  \|  \|  \|  \| \|  \|  \|  \|  \|  \|  \|  \| \| Tukey's multiple comparisons test \| Mean Diff. \| 95.00% CI of diff. \| Below threshold? \| Summary \| Adjusted P Value \|  \| \| - - vs. + - \| -168.6 \| -210.4 to -126.7 \| Yes \| **** \| <0.0001 \| G-H \| \| - - vs. + + \| -169.8 \| -211.7 to -127.9 \| Yes \| **** \| <0.0001 \| G-I \| \| + - vs. + + \| -1.251 \| -43.12 to 40.62 \| No \| ns \| 0.9969 \| H-I \| |
| **Figure 1F. BV2 (0.5 μM Selexipag, 3 hr), TNF-α mRNA level** |
| \| Number of families \| 1 \|  \|  \|  \|  \|  \| \| --- \| --- \| --- \| --- \| --- \| --- \| --- \| \| Number of comparisons per family \| 3 \|  \|  \|  \|  \|  \| \| Alpha \| 0.05 \|  \|  \|  \|  \|  \| \|  \|  \|  \|  \|  \|  \|  \| \| Tukey's multiple comparisons test \| Mean Diff. \| 95.00% CI of diff. \| Below threshold? \| Summary \| Adjusted P Value \|  \| \| - - vs. + - \| -104.6 \| -117.9 to -91.42 \| Yes \| **** \| <0.0001 \| J-K \| \| - - vs. + + \| -99.25 \| -112.5 to -86.03 \| Yes \| **** \| <0.0001 \| J-L \| \| + - vs. + + \| 5.385 \| -7.832 to 18.60 \| No \| ns \| 0.5686 \| K-L \| |
| **Figure 1G. BV2 (0.5 μM Selexipag, 24 hr), IL-1β mRNA level** |
| \| Number of families \| 1 \|  \|  \|  \|  \|  \| \| --- \| --- \| --- \| --- \| --- \| --- \| --- \| \| Number of comparisons per family \| 3 \|  \|  \|  \|  \|  \| \| Alpha \| 0.05 \|  \|  \|  \|  \|  \| \|  \|  \|  \|  \|  \|  \|  \| \| Tukey's multiple comparisons test \| Mean Diff. \| 95.00% CI of diff. \| Below threshold? \| Summary \| Adjusted P Value \|  \| \| - - vs. + - \| -120.3 \| -160.8 to -79.75 \| Yes \| **** \| <0.0001 \| A-B \| \| - - vs. + + \| -26.33 \| -66.87 to 14.22 \| No \| ns \| 0.2528 \| A-C \| \| + - vs. + + \| 93.97 \| 53.43 to 134.5 \| Yes \| **** \| <0.0001 \| B-C \| |
| **Figure 1G. BV2 (0.5 μM Selexipag, 24 hr), IL-6 mRNA level** |
| \| Number of families \| 1 \|  \|  \|  \|  \|  \| \| --- \| --- \| --- \| --- \| --- \| --- \| --- \| \| Number of comparisons per family \| 3 \|  \|  \|  \|  \|  \| \| Alpha \| 0.05 \|  \|  \|  \|  \|  \| \|  \|  \|  \|  \|  \|  \|  \| \| Tukey's multiple comparisons test \| Mean Diff. \| 95.00% CI of diff. \| Below threshold? \| Summary \| Adjusted P Value \|  \| \| - - vs. + - \| -18.04 \| -27.60 to -8.482 \| Yes \| *** \| 0.0003 \| D-E \| \| - - vs. + + \| -5.857 \| -15.42 to 3.703 \| No \| ns \| 0.2913 \| D-F \| \| + - vs. + + \| 12.18 \| 2.625 to 21.74 \| Yes \| * \| 0.0112 \| E-F \| |
| **Figure 1G. BV2 (0.5 μM Selexipag, 24 hr), COX-2 mRNA level** |
| \| Number of families \| 1 \|  \|  \|  \|  \|  \| \| --- \| --- \| --- \| --- \| --- \| --- \| --- \| \| Number of comparisons per family \| 3 \|  \|  \|  \|  \|  \| \| Alpha \| 0.05 \|  \|  \|  \|  \|  \| \|  \|  \|  \|  \|  \|  \|  \| \| Tukey's multiple comparisons test \| Mean Diff. \| 95.00% CI of diff. \| Below threshold? \| Summary \| Adjusted P Value \|  \| \| - - vs. + - \| -26.98 \| -38.77 to -15.19 \| Yes \| *** \| <0.0001 \| G-H \| \| - - vs. + + \| -6.264 \| -18.05 to 5.527 \| No \| ns \| 0.3901 \| G-I \| \| + - vs. + + \| 20.72 \| 8.926 to 32.51 \| Yes \| *** \| 0.0007 \| H-I \| |
| **Figure 1G. BV2 (0.5 μM Selexipag, 24 hr), TNF-α mRNA level** |
| \| Number of families \| 1 \|  \|  \|  \|  \|  \| \| --- \| --- \| --- \| --- \| --- \| --- \| --- \| \| Number of comparisons per family \| 3 \|  \|  \|  \|  \|  \| \| Alpha \| 0.05 \|  \|  \|  \|  \|  \| \|  \|  \|  \|  \|  \|  \|  \| \| Tukey's multiple comparisons test \| Mean Diff. \| 95.00% CI of diff. \| Below threshold? \| Summary \| Adjusted P Value \|  \| \| - - vs. + - \| -10.50 \| -16.45 to -4.551 \| Yes \| *** \| 0.0007 \| J-K \| \| - - vs. + + \| -3.541 \| -9.701 to 2.619 \| No \| ns \| 0.3333 \| J-L \| \| + - vs. + + \| 6.960 \| 0.8007 to 13.12 \| Yes \| * \| 0.0252 \| K-L \| |
| **Figure 1H. PMC (5 μM Selexipag, 6 hr), IL-1β mRNA level** |
| \| Number of families \| 1 \|  \|  \|  \|  \|  \| \| --- \| --- \| --- \| --- \| --- \| --- \| --- \| \| Number of comparisons per family \| 3 \|  \|  \|  \|  \|  \| \| Alpha \| 0.05 \|  \|  \|  \|  \|  \| \|  \|  \|  \|  \|  \|  \|  \| \| Tukey's multiple comparisons test \| Mean diff. \| 95.00% CI of diff. \| Below threshold? \| Summary \| Adjusted P Value \|  \| \| - - vs. + - \| -88.05 \| -104.8 to -71.31 \| Yes \| **** \| <0.0001 \| A-B \| \| - - vs. + + \| -59.38 \| -76.12 to -42.64 \| Yes \| **** \| <0.0001 \| A-C \| \| + - vs. + + \| 28.67 \| 11.93 to 45.41 \| Yes \| *** \| 0.0009 \| B-C \| |
| **Figure 1I. PMC (5 μM Selexipag, 6 hr), IL-6 mRNA level** |
| \| Number of families \| 1 \|  \|  \|  \|  \|  \| \| --- \| --- \| --- \| --- \| --- \| --- \| --- \| \| Number of comparisons per family \| 3 \|  \|  \|  \|  \|  \| \| Alpha \| 0.05 \|  \|  \|  \|  \|  \| \|  \|  \|  \|  \|  \|  \|  \| \| Tukey's multiple comparisons test \| Mean diff. \| 95.00% CI of diff. \| Below threshold? \| Summary \| Adjusted P Value \|  \| \| - - vs. + - \| -297.1 \| -332.4 to -261.9 \| Yes \| **** \| <0.0001 \| A-B \| \| - - vs. + + \| -90.74 \| -126.0 to -55.48 \| Yes \| **** \| <0.0001 \| A-C \| \| + - vs. + + \| 206.4 \| 171.1 to 241.6 \| Yes \| **** \| <0.0001 \| B-C \| |
| **Figure 1J. PMC (5 μM Selexipag, 6 hr), COX-2 mRNA level** |
| \| Number of families \| 1 \|  \|  \|  \|  \|  \| \| --- \| --- \| --- \| --- \| --- \| --- \| --- \| \| Number of comparisons per family \| 3 \|  \|  \|  \|  \|  \| \| Alpha \| 0.05 \|  \|  \|  \|  \|  \| \|  \|  \|  \|  \|  \|  \|  \| \| Tukey's multiple comparisons test \| Mean diff. \| 95.00% CI of diff. \| Below threshold? \| Summary \| Adjusted P Value \|  \| \| - - vs. + - \| -72.75 \| -79.66 to -65.84 \| Yes \| **** \| <0.0001 \| A-B \| \| - - vs. + + \| -35.37 \| -42.28 to -28.46 \| Yes \| **** \| <0.0001 \| A-C \| \| + - vs. + + \| 37.38 \| 30.47 to 44.29 \| Yes \| **** \| <0.0001 \| B-C \| |
| **Figure 1K. PMC (5 μM Selexipag, 6 hr), TNF-α mRNA level** |
| \| Number of families \| 1 \|  \|  \|  \|  \|  \| \| --- \| --- \| --- \| --- \| --- \| --- \| --- \| \| Number of comparisons per family \| 3 \|  \|  \|  \|  \|  \| \| Alpha \| 0.05 \|  \|  \|  \|  \|  \| \|  \|  \|  \|  \|  \|  \|  \| \| Tukey's multiple comparisons test \| Mean diff. \| 95.00% CI of diff. \| Below threshold? \| Summary \| Adjusted P Value \|  \| \| - - vs. + - \| -195.3 \| -236.3 to -154.2 \| Yes \| **** \| <0.0001 \| A-B \| \| - - vs. + + \| -82.81 \| -123.8 to -41.78 \| Yes \| *** \| 0.0001 \| A-C \| \| + - vs. + + \| 112.5 \| 71.43 to 153.5 \| Yes \| **** \| <0.0001 \| B-C \| |
| **Figure 2A, C. Iba-1 fluorescence intensity in the Cortex of C57BL/6N mice** |
| \| Number of families \| 1 \|  \|  \|  \|  \|  \| \| --- \| --- \| --- \| --- \| --- \| --- \| --- \| \| Number of comparisons per family \| 3 \|  \|  \|  \|  \|  \| \| Alpha \| 0.05 \|  \|  \|  \|  \|  \| \|  \|  \|  \|  \|  \|  \|  \| \| Tukey's multiple comparisons test \| Mean Diff. \| 95.00% CI of diff. \| Below threshold? \| Summary \| Adjusted P Value \|  \| \| - - vs. + - \| -77.91 \| -108.5 to -47.35 \| Yes \| **** \| <0.0001 \| A-B \| \| - - vs. + + \| -43.14 \| -73.70 to -12.58 \| Yes \| ** \| 0.0035 \| A-C \| \| + - vs. + + \| 34.77 \| 4.210 to 65.33 \| Yes \| * \| 0.0221 \| B-C \| |
| **Figure 2B, C. Iba-1 fluorescence intensity in the Hippocampal CA1 of C57BL/6N mice** |
| \| Number of families \| 1 \|  \|  \|  \|  \|  \| \| --- \| --- \| --- \| --- \| --- \| --- \| --- \| \| Number of comparisons per family \| 3 \|  \|  \|  \|  \|  \| \| Alpha \| 0.05 \|  \|  \|  \|  \|  \| \|  \|  \|  \|  \|  \|  \|  \| \| Tukey's multiple comparisons test \| Mean Diff. \| 95.00% CI of diff. \| Below threshold? \| Summary \| Adjusted P Value \|  \| \| - - vs. + - \| -114.2 \| -147.4 to -80.89 \| Yes \| **** \| <0.0001 \| D-E \| \| - - vs. + + \| -64.98 \| -98.26 to -31.70 \| Yes \| **** \| <0.0001 \| D-F \| \| + - vs. + + \| 49.18 \| 15.91 to 82.46 \| Yes \| ** \| 0.0022 \| E-F \| |
| **Figure 2B, C. Iba-1 fluorescence intensity in the Hippocampal CA2 of C57BL/6N mice** |
| \| Number of families \| 1 \|  \|  \|  \|  \|  \| \| --- \| --- \| --- \| --- \| --- \| --- \| --- \| \| Number of comparisons per family \| 3 \|  \|  \|  \|  \|  \| \| Alpha \| 0.05 \|  \|  \|  \|  \|  \| \|  \|  \|  \|  \|  \|  \|  \| \| Tukey's multiple comparisons test \| Mean Diff. \| 95.00% CI of diff. \| Below threshold? \| Summary \| Adjusted P Value \|  \| \| - - vs. + - \| -103.4 \| -150.6 to -56.12 \| Yes \| **** \| <0.0001 \| G-H \| \| - - vs. + + \| -63.95 \| -111.8 to -16.09 \| Yes \| ** \| 0.0060 \| G-I \| \| + - vs. + + \| 39.41 \| -8.453 to 87.27 \| No \| ns \| 0.1260 \| H-I \| |
| **Figure 2B, C. Iba-1 fluorescence intensity in the Hippocampal CA3 of C57BL/6N mice** |
| \| Number of families \| 1 \|  \|  \|  \|  \|  \| \| --- \| --- \| --- \| --- \| --- \| --- \| --- \| \| Number of comparisons per family \| 3 \|  \|  \|  \|  \|  \| \| Alpha \| 0.05 \|  \|  \|  \|  \|  \| \|  \|  \|  \|  \|  \|  \|  \| \| Tukey's multiple comparisons test \| Mean Diff. \| 95.00% CI of diff. \| Below threshold? \| Summary \| Adjusted P Value \|  \| \| - - vs. + - \| -132.6 \| -177.2 to -87.98 \| Yes \| **** \| <0.0001 \| J-K \| \| - - vs. + + \| -97.55 \| -142.7 to -52.35 \| Yes \| **** \| <0.0001 \| J-L \| \| + - vs. + + \| 35.05 \| -10.15 to 80.25 \| No \| ns \| 0.1579 \| K-L \| |
| **Figure 3B, C. Iba-1 fluorescence intensity in the Hippocampal CA4 of C57BL/6N mice** |
| \| Number of families \| 1 \|  \|  \|  \|  \|  \| \| --- \| --- \| --- \| --- \| --- \| --- \| --- \| \| Number of comparisons per family \| 3 \|  \|  \|  \|  \|  \| \| Alpha \| 0.05 \|  \|  \|  \|  \|  \| \|  \|  \|  \|  \|  \|  \|  \| \| Tukey's multiple comparisons test \| Mean Diff. \| 95.00% CI of diff. \| Below threshold? \| Summary \| Adjusted P Value \|  \| \| - - vs. + - \| -141.4 \| -181.5 to -101.4 \| Yes \| **** \| <0.0001 \| M-N \| \| - - vs. + + \| -102.0 \| -142.5 to -61.45 \| Yes \| **** \| <0.0001 \| M-O \| \| + - vs. + + \| 39.44 \| -1.098 to 79.99 \| No \| ns \| 0.0581 \| N-O \| |
| **Figure 2B, C. Iba-1 fluorescence intensity in the Hippocampal DG of C57BL/6N mice** |
| \| Number of families \| 1 \|  \|  \|  \|  \|  \| \| --- \| --- \| --- \| --- \| --- \| --- \| --- \| \| Number of comparisons per family \| 3 \|  \|  \|  \|  \|  \| \| Alpha \| 0.05 \|  \|  \|  \|  \|  \| \|  \|  \|  \|  \|  \|  \|  \| \| Tukey's multiple comparisons test \| Mean Diff. \| 95.00% CI of diff. \| Below threshold? \| Summary \| Adjusted P Value \|  \| \| - - vs. + - \| -94.43 \| -120.1 to -68.71 \| Yes \| **** \| <0.0001 \| P-Q \| \| - - vs. + + \| -58.13 \| -83.84 to -32.41 \| Yes \| **** \| <0.0001 \| P-R \| \| + - vs. + + \| 36.30 \| 10.92 to 61.68 \| Yes \| ** \| 0.0031 \| Q-R \| |
| **Figure 2A, D. Iba-1 labeled area in the Cortex of C57BL/6N mice** |
| \| Number of families \| 1 \|  \|  \|  \|  \|  \| \| --- \| --- \| --- \| --- \| --- \| --- \| --- \| \| Number of comparisons per family \| 3 \|  \|  \|  \|  \|  \| \| Alpha \| 0.05 \|  \|  \|  \|  \|  \| \|  \|  \|  \|  \|  \|  \|  \| \| Tukey's multiple comparisons test \| Mean Diff. \| 95.00% CI of diff. \| Below threshold? \| Summary \| Adjusted P Value \|  \| \| - - vs. + - \| -3.187 \| -4.130 to -2.243 \| Yes \| **** \| <0.0001 \| A-B \| \| - - vs. + + \| -1.952 \| -2.895 to -1.009 \| Yes \| **** \| <0.0001 \| A-C \| \| + - vs. + + \| 1.235 \| 0.2914 to 2.178 \| Yes \| ** \| 0.0072 \| B-C \| |
| **Figure 2B, D. Iba-1 labeled area in the Hippocampal CA1 of C57BL/6N mice** |
| \| Number of families \| 1 \|  \|  \|  \|  \|  \| \| --- \| --- \| --- \| --- \| --- \| --- \| --- \| \| Number of comparisons per family \| 3 \|  \|  \|  \|  \|  \| \| Alpha \| 0.05 \|  \|  \|  \|  \|  \| \|  \|  \|  \|  \|  \|  \|  \| \| Tukey's multiple comparisons test \| Mean diff. \| 95.00% CI of diff. \| Below threshold? \| Summary \| Adjusted P Value \|  \| \| - - vs. + - \| -3.065 \| -3.960 to -2.169 \| Yes \| **** \| <0.0001 \| D-E \| \| - - vs. + + \| -1.729 \| -2.624 to -0.8334 \| Yes \| **** \| <0.0001 \| D-F \| \| + - vs. + + \| 1.336 \| 0.4401 to 2.231 \| Yes \| ** \| 0.0020 \| E-F \| |
| **Figure 2B, D. Iba-1 labeled area in the Hippocampal CA2 of C57BL/6N mice** |
| \| Number of families \| 1 \|  \|  \|  \|  \|  \| \| --- \| --- \| --- \| --- \| --- \| --- \| --- \| \| Number of comparisons per family \| 3 \|  \|  \|  \|  \|  \| \| Alpha \| 0.05 \|  \|  \|  \|  \|  \| \|  \|  \|  \|  \|  \|  \|  \| \| Tukey's multiple comparisons test \| Mean diff. \| 95.00% CI of diff. \| Below threshold? \| Summary \| Adjusted P Value \|  \| \| - - vs. + - \| -2.584 \| -3.764 to -1.403 \| Yes \| **** \| <0.0001 \| G-H \| \| - - vs. + + \| -1.585 \| -2.780 to -0.3890 \| Yes \| ** \| 0.0065 \| G-I \| \| + - vs. + + \| 0.9989 \| -0.1968 to 2.195 \| No \| ns \| 0.1189 \| H-I \| |
| **Figure 2B, D. Iba-1 labeled area in the Hippocampal CA3 of C57BL/6N mice** |
| \| Number of families \| 1 \|  \|  \|  \|  \|  \| \| --- \| --- \| --- \| --- \| --- \| --- \| --- \| \| Number of comparisons per family \| 3 \|  \|  \|  \|  \|  \| \| Alpha \| 0.05 \|  \|  \|  \|  \|  \| \|  \|  \|  \|  \|  \|  \|  \| \| Tukey's multiple comparisons test \| Mean diff. \| 95.00% CI of diff. \| Below threshold? \| Summary \| Adjusted P Value \|  \| \| - - vs. + - \| -2.928 \| -3.911 to -1.945 \| Yes \| **** \| <0.0001 \| J-K \| \| - - vs. + + \| -2.144 \| -3.140 to -1.148 \| Yes \| **** \| <0.0001 \| J-L \| \| + - vs. + + \| 0.7841 \| -0.2120 to 1.780 \| No \| ns \| 0.1495 \| K-L \| |
| **Figure 2B, D. Iba-1 labeled area in the Hippocampal CA4 of C57BL/6N mice** |
| \| Number of families \| 1 \|  \|  \|  \|  \|  \| \| --- \| --- \| --- \| --- \| --- \| --- \| --- \| \| Number of comparisons per family \| 3 \|  \|  \|  \|  \|  \| \| Alpha \| 0.05 \|  \|  \|  \|  \|  \| \|  \|  \|  \|  \|  \|  \|  \| \| Tukey's multiple comparisons test \| Mean diff. \| 95.00% CI of diff. \| Below threshold? \| Summary \| Adjusted P Value \|  \| \| - - vs. + - \| -5.157 \| -6.615 to -3.699 \| Yes \| **** \| <0.0001 \| M-N \| \| - - vs. + + \| -3.735 \| -5.212 to -2.258 \| Yes \| **** \| <0.0001 \| M-O \| \| + - vs. + + \| 1.422 \| -0.05526 to 2.899 \| No \| ns \| 0.0615 \| N-O \| |
| **Figure 2B, D. Iba-1 labeled area in the Hippocampal DG of C57BL/6N mice** |
| \| Number of families \| 1 \|  \|  \|  \|  \|  \| \| --- \| --- \| --- \| --- \| --- \| --- \| --- \| \| Number of comparisons per family \| 3 \|  \|  \|  \|  \|  \| \| Alpha \| 0.05 \|  \|  \|  \|  \|  \| \|  \|  \|  \|  \|  \|  \|  \| \| Tukey's multiple comparisons test \| Mean diff. \| 95.00% CI of diff. \| Below threshold? \| Summary \| Adjusted P Value \|  \| \| - - vs. + - \| -3.236 \| -4.127 to -2.344 \| Yes \| **** \| <0.0001 \| P-Q \| \| - - vs. + + \| -2.406 \| -3.298 to -1.514 \| Yes \| **** \| <0.0001 \| P-R \| \| + - vs. + + \| 0.8297 \| -0.06214 to 1.721 \| No \| ns \| 0.0733 \| Q-R \| |
| **Figure 2A, E. Iba-1 positive cells in the Cortex of C57BL/6N mice** |
| \| Number of families \| 1 \|  \|  \|  \|  \|  \| \| --- \| --- \| --- \| --- \| --- \| --- \| --- \| \| Number of comparisons per family \| 3 \|  \|  \|  \|  \|  \| \| Alpha \| 0.05 \|  \|  \|  \|  \|  \| \|  \|  \|  \|  \|  \|  \|  \| \| Tukey's multiple comparisons test \| Mean Diff. \| 95.00% CI of diff. \| Below threshold? \| Summary \| Adjusted P Value \|  \| \| - - vs. + - \| -434.8 \| -601.9 to -267.7 \| Yes \| **** \| <0.0001 \| A-B \| \| - - vs. + + \| -344.1 \| -511.2 to -177.0 \| Yes \| **** \| <0.0001 \| A-C \| \| + - vs. + + \| 90.76 \| -76.34 to 257.9 \| No \| ns \| 0.3972 \| B-C \| |
| **Figure 2B, E. Iba-1 positive cells in the Hippocampal CA1 of C57BL/6N mice** |
| \| Number of families \| 1 \|  \|  \|  \|  \|  \| \| --- \| --- \| --- \| --- \| --- \| --- \| --- \| \| Number of comparisons per family \| 3 \|  \|  \|  \|  \|  \| \| Alpha \| 0.05 \|  \|  \|  \|  \|  \| \|  \|  \|  \|  \|  \|  \|  \| \| Tukey's multiple comparisons test \| Mean Diff. \| 95.00% CI of diff. \| Below threshold? \| Summary \| Adjusted P Value \|  \| \| - - vs. + - \| -461.9 \| -632.1 to -291.6 \| Yes \| **** \| <0.0001 \| D-E \| \| - - vs. + + \| -377.6 \| -547.8 to -207.3 \| Yes \| **** \| <0.0001 \| D-F \| \| + - vs. + + \| 84.29 \| -85.99 to 254.6 \| No \| ns \| 0.4633 \| E-F \| |
| **Figure 2B, E. Iba-1 positive cells in the Hippocampal CA2 of C57BL/6N mice** |
| \| Number of families \| 1 \|  \|  \|  \|  \|  \| \| --- \| --- \| --- \| --- \| --- \| --- \| --- \| \| Number of comparisons per family \| 3 \|  \|  \|  \|  \|  \| \| Alpha \| 0.05 \|  \|  \|  \|  \|  \| \|  \|  \|  \|  \|  \|  \|  \| \| Tukey's multiple comparisons test \| Mean Diff. \| 95.00% CI of diff. \| Below threshold? \| Summary \| Adjusted P Value \|  \| \| - - vs. + - \| -452.1 \| -707.5 to -196.7 \| Yes \| *** \| 0.0002 \| G-H \| \| - - vs. + + \| -425.1 \| -680.4 to -169.7 \| Yes \| *** \| 0.0005 \| G-I \| \| + - vs. + + \| 27.08 \| -228.3 to 282.5 \| No \| ns \| 0.9648 \| H-I \| |
| **Figure 2B, E. Iba-1 positive cells in the Hippocampal CA3 of C57BL/6N mice** |
| \| Number of families \| 1 \|  \|  \|  \|  \|  \| \| --- \| --- \| --- \| --- \| --- \| --- \| --- \| \| Number of comparisons per family \| 3 \|  \|  \|  \|  \|  \| \| Alpha \| 0.05 \|  \|  \|  \|  \|  \| \|  \|  \|  \|  \|  \|  \|  \| \| Tukey's multiple comparisons test \| Mean Diff. \| 95.00% CI of diff. \| Below threshold? \| Summary \| Adjusted P Value \|  \| \| - - vs. + - \| -509.7 \| -724.8 to -294.5 \| Yes \| **** \| <0.0001 \| J-K \| \| - - vs. + + \| -509.1 \| -724.2 to -293.9 \| Yes \| **** \| <0.0001 \| J-L \| \| + - vs. + + \| 0.5944 \| -214.6 to 215.7 \| No \| ns \| >0.9999 \| K-L \| |
| **Figure 2B, E. Iba-1 positive cells in the Hippocampal CA4 of C57BL/6N mice** |
| \| Number of families \| 1 \|  \|  \|  \|  \|  \| \| --- \| --- \| --- \| --- \| --- \| --- \| --- \| \| Number of comparisons per family \| 3 \|  \|  \|  \|  \|  \| \| Alpha \| 0.05 \|  \|  \|  \|  \|  \| \|  \|  \|  \|  \|  \|  \|  \| \| Tukey's multiple comparisons test \| Mean Diff. \| 95.00% CI of diff. \| Below threshold? \| Summary \| Adjusted P Value \|  \| \| - - vs. + - \| -948.7 \| -1283 to -614.0 \| Yes \| **** \| <0.0001 \| M-N \| \| - - vs. + + \| -1011 \| -1346 to -676.7 \| Yes \| **** \| <0.0001 \| M-O \| \| + - vs. + + \| -62.76 \| -393.2 to 267.6 \| No \| ns \| 0.8914 \| N-O \| |
| **Figure 2B, E. Iba-1 positive cells in the Hippocampal DG of C57BL/6N mice** |
| \| Number of families \| 1 \|  \|  \|  \|  \|  \| \| --- \| --- \| --- \| --- \| --- \| --- \| --- \| \| Number of comparisons per family \| 3 \|  \|  \|  \|  \|  \| \| Alpha \| 0.05 \|  \|  \|  \|  \|  \| \|  \|  \|  \|  \|  \|  \|  \| \| Tukey's multiple comparisons test \| Mean Diff. \| 95.00% CI of diff. \| Below threshold? \| Summary \| Adjusted P Value \|  \| \| - - vs. + - \| -441.0 \| -588.8 to -293.2 \| Yes \| **** \| <0.0001 \| P-Q \| \| - - vs. + + \| -423.5 \| -571.2 to -275.7 \| Yes \| **** \| <0.0001 \| P-R \| \| + - vs. + + \| 17.50 \| -130.3 to 165.3 \| No \| ns \| 0.9562 \| Q-R \| |
| **Figure 3A, C. GFAP fluorescence intensity in the Cortex of C57BL/6N mice** |
| \| Number of families \| 1 \|  \|  \|  \|  \| \| --- \| --- \| --- \| --- \| --- \| --- \| \| Number of comparisons per family \| 3 \|  \|  \|  \|  \| \| Alpha \| 0.05 \|  \|  \|  \|  \| \|  \|  \|  \|  \|  \|  \| \| Newman-Keuls multiple comparisons test \| Mean diff. \| Below threshold? \| Summary \|  \|  \| \| - - vs. + - \| -111.5 \| Yes \| **** \|  \| A-B \| \| - - vs. + + \| -58.33 \| Yes \| * \|  \| A-C \| \| + - vs. + + \| 53.13 \| Yes \| * \|  \| B-C \| |
| **Figure 3B, C. GFAP fluorescence intensity in the Hippocampal CA1 of C57BL/6N mice** |
| \| Number of families \| 1 \|  \|  \|  \|  \|  \| \| --- \| --- \| --- \| --- \| --- \| --- \| --- \| \| Number of comparisons per family \| 3 \|  \|  \|  \|  \|  \| \| Alpha \| 0.05 \|  \|  \|  \|  \|  \| \|  \|  \|  \|  \|  \|  \|  \| \| Tukey's multiple comparisons test \| Mean Diff. \| 95.00% CI of diff. \| Below threshold? \| Summary \| Adjusted P Value \|  \| \| - - vs. + - \| -83.17 \| -109.6 to -56.78 \| Yes \| **** \| <0.0001 \| D-E \| \| - - vs. + + \| -39.32 \| -65.70 to -12.93 \| Yes \| ** \| 0.0020 \| D-F \| \| + - vs. + + \| 43.85 \| 17.46 to 70.24 \| Yes \| *** \| 0.0005 \| E-F \| |
| **Figure 3B, C. GFAP fluorescence intensity in the Hippocampal CA2 of C57BL/6N mice** |
| \| Number of families \| 1 \|  \|  \|  \|  \|  \| \| --- \| --- \| --- \| --- \| --- \| --- \| --- \| \| Number of comparisons per family \| 3 \|  \|  \|  \|  \|  \| \| Alpha \| 0.05 \|  \|  \|  \|  \|  \| \|  \|  \|  \|  \|  \|  \|  \| \| Tukey's multiple comparisons test \| Mean Diff. \| 95.00% CI of diff. \| Below threshold? \| Summary \| Adjusted P Value \|  \| \| - - vs. + - \| -101.6 \| -130.1 to -73.12 \| Yes \| **** \| <0.0001 \| G-H \| \| - - vs. + + \| -53.70 \| -82.21 to -25.19 \| Yes \| **** \| <0.0001 \| G-I \| \| + - vs. + + \| 47.93 \| 19.42 to 76.43 \| Yes \| *** \| 0.0005 \| H-I \| |
| **Figure 3B, C. GFAP fluorescence intensity in the Hippocampal CA3 of C57BL/6N mice** |
| \| Number of families \| 1 \|  \|  \|  \|  \|  \| \| --- \| --- \| --- \| --- \| --- \| --- \| --- \| \| Number of comparisons per family \| 3 \|  \|  \|  \|  \|  \| \| Alpha \| 0.05 \|  \|  \|  \|  \|  \| \|  \|  \|  \|  \|  \|  \|  \| \| Tukey's multiple comparisons test \| Mean Diff. \| 95.00% CI of diff. \| Below threshold? \| Summary \| Adjusted P Value \|  \| \| - - vs. + - \| -135.2 \| -192.2 to -78.13 \| Yes \| **** \| <0.0001 \| J-K \| \| - - vs. + + \| -109.5 \| -166.6 to -52.52 \| Yes \| **** \| <0.0001 \| J-L \| \| + - vs. + + \| 25.61 \| -31.42 to 82.63 \| No \| ns \| 0.5300 \| K-L \| |
| **Figure 3B, C. GFAP fluorescence intensity in the Hippocampal CA4 of C57BL/6N mice** |
| \| Number of families \| 1 \|  \|  \|  \|  \|  \| \| --- \| --- \| --- \| --- \| --- \| --- \| --- \| \| Number of comparisons per family \| 3 \|  \|  \|  \|  \|  \| \| Alpha \| 0.05 \|  \|  \|  \|  \|  \| \|  \|  \|  \|  \|  \|  \|  \| \| Tukey's multiple comparisons test \| Mean Diff. \| 95.00% CI of diff. \| Below threshold? \| Summary \| Adjusted P Value \|  \| \| - - vs. + - \| -131.9 \| -173.4 to -90.40 \| Yes \| **** \| <0.0001 \| M-N \| \| - - vs. + + \| -91.55 \| -132.5 to -50.57 \| Yes \| **** \| <0.0001 \| M-O \| \| + - vs. + + \| 40.34 \| -0.6341 to 81.32 \| No \| ns \| 0.0545 \| N-O \| |
| **Figure 3B, C. GFAP fluorescence intensity in the Hippocampal DG of C57BL/6N mice** |
| \| Number of families \| 1 \|  \|  \|  \|  \|  \| \| --- \| --- \| --- \| --- \| --- \| --- \| --- \| \| Number of comparisons per family \| 3 \|  \|  \|  \|  \|  \| \| Alpha \| 0.05 \|  \|  \|  \|  \|  \| \|  \|  \|  \|  \|  \|  \|  \| \| Tukey's multiple comparisons test \| Mean Diff. \| 95.00% CI of diff. \| Below threshold? \| Summary \| Adjusted P Value \|  \| \| - - vs. + - \| -90.69 \| -116.9 to -64.49 \| Yes \| **** \| <0.0001 \| P-Q \| \| - - vs. + + \| -54.39 \| -80.59 to -28.19 \| Yes \| **** \| <0.0001 \| P-R \| \| + - vs. + + \| 36.30 \| 10.10 to 62.50 \| Yes \| ** \| 0.0042 \| Q-R \| |
| **Figure 3A, D. GFAP labeled area in the Cortex of C57BL/6N mice** |
| \| Number of families \| 1 \|  \|  \|  \|  \|  \| \| --- \| --- \| --- \| --- \| --- \| --- \| --- \| \| Number of comparisons per family \| 3 \|  \|  \|  \|  \|  \| \| Alpha \| 0.05 \|  \|  \|  \|  \|  \| \|  \|  \|  \|  \|  \|  \|  \| \| Tukey's multiple comparisons test \| Mean Diff. \| 95.00% CI of diff. \| Below threshold? \| Summary \| Adjusted P Value \|  \| \| - - vs. + - \| -0.4706 \| -0.7330 to -0.2081 \| Yes \| *** \| 0.0002 \| P-Q \| \| - - vs. + + \| -0.1599 \| -0.4224 to 0.1026 \| No \| ns \| 0.3148 \| P-R \| \| + - vs. + + \| 0.3107 \| 0.04817 to 0.5731 \| Yes \| * \| 0.0166 \| Q-R \| |
| **Figure 3B, D. GFAP labeled area in the Hippocampal CA1 of C57BL/6N mice** |
| \| Number of families \| 1 \|  \|  \|  \|  \|  \| \| --- \| --- \| --- \| --- \| --- \| --- \| --- \| \| Number of comparisons per family \| 3 \|  \|  \|  \|  \|  \| \| Alpha \| 0.05 \|  \|  \|  \|  \|  \| \|  \|  \|  \|  \|  \|  \|  \| \| Tukey's multiple comparisons test \| Mean Diff. \| 95.00% CI of diff. \| Below threshold? \| Summary \| Adjusted P Value \|  \| \| - - vs. + - \| -10.23 \| -13.12 to -7.335 \| Yes \| **** \| <0.0001 \| D-E \| \| - - vs. + + \| -4.899 \| -7.793 to -2.005 \| Yes \| *** \| 0.0004 \| D-F \| \| + - vs. + + \| 5.330 \| 2.436 to 8.224 \| Yes \| *** \| 0.0001 \| E-F \| |
| **Figure 3B, D. GFAP labeled area in the Hippocampal CA2 of C57BL/6N mice** |
| \| Number of families \| 1 \|  \|  \|  \|  \|  \| \| --- \| --- \| --- \| --- \| --- \| --- \| --- \| \| Number of comparisons per family \| 3 \|  \|  \|  \|  \|  \| \| Alpha \| 0.05 \|  \|  \|  \|  \|  \| \|  \|  \|  \|  \|  \|  \|  \| \| Tukey's multiple comparisons test \| Mean Diff. \| 95.00% CI of diff. \| Below threshold? \| Summary \| Adjusted P Value \|  \| \| - - vs. + - \| -14.38 \| -18.11 to -10.66 \| Yes \| **** \| <0.0001 \| G-H \| \| - - vs. + + \| -7.058 \| -10.79 to -3.330 \| Yes \| **** \| <0.0001 \| G-I \| \| + - vs. + + \| 7.326 \| 3.598 to 11.05 \| Yes \| **** \| <0.0001 \| H-I \| |
| **Figure 3B, D. GFAP labeled area in the Hippocampal CA3 of C57BL/6N mice** |
| \| Number of families \| 1 \|  \|  \|  \|  \|  \| \| --- \| --- \| --- \| --- \| --- \| --- \| --- \| \| Number of comparisons per family \| 3 \|  \|  \|  \|  \|  \| \| Alpha \| 0.05 \|  \|  \|  \|  \|  \| \|  \|  \|  \|  \|  \|  \|  \| \| Tukey's multiple comparisons test \| Mean Diff. \| 95.00% CI of diff. \| Below threshold? \| Summary \| Adjusted P Value \|  \| \| - - vs. + - \| -9.257 \| -13.10 to -5.414 \| Yes \| **** \| <0.0001 \| J-K \| \| - - vs. + + \| -6.193 \| -10.04 to -2.350 \| Yes \| *** \| 0.0008 \| J-L \| \| + - vs. + + \| 3.064 \| -0.7785 to 6.907 \| No \| ns \| 0.1426 \| K-L \| |
| **Figure 3B, D. GFAP labeled area in the Hippocampal CA4** |
| \| Number of families \| 1 \|  \|  \|  \|  \|  \| \| --- \| --- \| --- \| --- \| --- \| --- \| --- \| \| Number of comparisons per family \| 3 \|  \|  \|  \|  \|  \| \| Alpha \| 0.05 \|  \|  \|  \|  \|  \| \|  \|  \|  \|  \|  \|  \|  \| \| Tukey's multiple comparisons test \| Mean Diff. \| 95.00% CI of diff. \| Below threshold? \| Summary \| Adjusted P Value \|  \| \| - - vs. + - \| -13.71 \| -22.29 to -5.124 \| Yes \| *** \| 0.0009 \| M-N \| \| - - vs. + + \| -5.484 \| -14.07 to 3.099 \| No \| ns \| 0.2809 \| M-O \| \| + - vs. + + \| 8.223 \| -0.4689 to 16.91 \| No \| ns \| 0.0673 \| N-O \| |
| **Figure 3B, D. GFAP labeled area in the Hippocampal DG of C57BL/6N mice** |
| \| Number of families \| 1 \|  \|  \|  \|  \|  \| \| --- \| --- \| --- \| --- \| --- \| --- \| --- \| \| Number of comparisons per family \| 3 \|  \|  \|  \|  \|  \| \| Alpha \| 0.05 \|  \|  \|  \|  \|  \| \|  \|  \|  \|  \|  \|  \|  \| \| Tukey's multiple comparisons test \| Mean Diff. \| 95.00% CI of diff. \| Below threshold? \| Summary \| Adjusted P Value \|  \| \| - - vs. + - \| -3.236 \| -4.127 to -2.344 \| Yes \| **** \| <0.0001 \| P-Q \| \| - - vs. + + \| -2.406 \| -3.298 to -1.514 \| Yes \| **** \| <0.0001 \| P-R \| \| + - vs. + + \| 0.8297 \| -0.06214 to 1.721 \| No \| ns \| 0.0733 \| Q-R \| |
| **Figure 3A, E. GFAP positive cells in the Cortex of C57BL/6N mice** |
| \| Number of families \| 1 \|  \|  \|  \|  \|  \| \| --- \| --- \| --- \| --- \| --- \| --- \| --- \| \| Number of comparisons per family \| 3 \|  \|  \|  \|  \|  \| \| Alpha \| 0.05 \|  \|  \|  \|  \|  \| \|  \|  \|  \|  \|  \|  \|  \| \| Tukey's multiple comparisons test \| Mean Diff. \| 95.00% CI of diff. \| Below threshold? \| Summary \| Adjusted P Value \|  \| \| - - vs. + - \| -24.83 \| -45.17 to -4.493 \| Yes \| * \| 0.0130 \| A-B \| \| - - vs. + + \| -12.39 \| -32.73 to 7.947 \| No \| ns \| 0.3147 \| A-C \| \| + - vs. + + \| 12.44 \| -7.899 to 32.78 \| No \| ns \| 0.3119 \| B-C \| |
| **Figure 3B, E. GFAP positive cells in the Hippocampal CA1 of C57BL/6N mice** |
| \| Number of families \| 1 \|  \|  \|  \|  \|  \| \| --- \| --- \| --- \| --- \| --- \| --- \| --- \| \| Number of comparisons per family \| 3 \|  \|  \|  \|  \|  \| \| Alpha \| 0.05 \|  \|  \|  \|  \|  \| \|  \|  \|  \|  \|  \|  \|  \| \| Tukey's multiple comparisons test \| Mean Diff. \| 95.00% CI of diff. \| Below threshold? \| Summary \| Adjusted P Value \|  \| \| - - vs. + - \| -34.78 \| -96.10 to 26.54 \| No \| ns \| 0.3659 \| D-E \| \| - - vs. + + \| 6.241 \| -55.08 to 67.56 \| No \| ns \| 0.9675 \| D-F \| \| + - vs. + + \| 41.02 \| -20.30 to 102.3 \| No \| ns \| 0.2498 \| E-F \| |
| **Figure 3B, E. GFAP positive cells in the Hippocampal CA2 of C57BL/6N mice** |
| \| Number of families \| 1 \|  \|  \|  \|  \|  \| \| --- \| --- \| --- \| --- \| --- \| --- \| --- \| \| Number of comparisons per family \| 3 \|  \|  \|  \|  \|  \| \| Alpha \| 0.05 \|  \|  \|  \|  \|  \| \|  \|  \|  \|  \|  \|  \|  \| \| Tukey's multiple comparisons test \| Mean Diff. \| 95.00% CI of diff. \| Below threshold? \| Summary \| Adjusted P Value \|  \| \| - - vs. + - \| -72.23 \| -161.3 to 16.79 \| No \| ns \| 0.1335 \| G-H \| \| - - vs. + + \| -29.78 \| -118.8 to 59.24 \| No \| ns \| 0.7014 \| G-I \| \| + - vs. + + \| 42.45 \| -46.57 to 131.5 \| No \| ns \| 0.4893 \| H-I \| |
| **Figure 3B, E. GFAP positive cells in the Hippocampal CA3 of C57BL/6N mice** |
| \| Number of families \| 1 \|  \|  \|  \|  \|  \| \| --- \| --- \| --- \| --- \| --- \| --- \| --- \| \| Number of comparisons per family \| 3 \|  \|  \|  \|  \|  \| \| Alpha \| 0.05 \|  \|  \|  \|  \|  \| \|  \|  \|  \|  \|  \|  \|  \| \| Tukey's multiple comparisons test \| Mean Diff. \| 95.00% CI of diff. \| Below threshold? \| Summary \| Adjusted P Value \|  \| \| - - vs. + - \| -234.3 \| -353.6 to -115.1 \| Yes \| **** \| <0.0001 \| J-K \| \| - - vs. + + \| -214.6 \| -333.8 to -95.31 \| Yes \| *** \| 0.0002 \| J-L \| \| + - vs. + + \| 19.78 \| -99.47 to 139.0 \| No \| ns \| 0.9160 \| K-L \| |
| **Figure 3B, E. GFAP positive cells in the Hippocampal CA4 of C57BL/6N mice** |
| \| Number of families \| 1 \|  \|  \|  \|  \|  \| \| --- \| --- \| --- \| --- \| --- \| --- \| --- \| \| Number of comparisons per family \| 3 \|  \|  \|  \|  \|  \| \| Alpha \| 0.05 \|  \|  \|  \|  \|  \| \|  \|  \|  \|  \|  \|  \|  \| \| Tukey's multiple comparisons test \| Mean Diff. \| 95.00% CI of diff. \| Below threshold? \| Summary \| Adjusted P Value \|  \| \| - - vs. + - \| 131.4 \| -269.1 to 531.9 \| No \| ns \| 0.7110 \| M-N \| \| - - vs. + + \| 2.774 \| -397.7 to 403.2 \| No \| ns \| 0.9998 \| M-O \| \| + - vs. + + \| -128.6 \| -529.1 to 271.9 \| No \| ns \| 0.7211 \| N-O \| |
| **Figure 3B, E. GFAP positive cells in the Hippocampal DG of C57BL/6N mice** |
| \| Number of families \| 1 \|  \|  \|  \|  \|  \| \| --- \| --- \| --- \| --- \| --- \| --- \| --- \| \| Number of comparisons per family \| 3 \|  \|  \|  \|  \|  \| \| Alpha \| 0.05 \|  \|  \|  \|  \|  \| \|  \|  \|  \|  \|  \|  \|  \| \| Tukey's multiple comparisons test \| Mean Diff. \| 95.00% CI of diff. \| Below threshold? \| Summary \| Adjusted P Value \|  \| \| - - vs. + - \| -162.5 \| -257.9 to -67.01 \| Yes \| *** \| 0.0004 \| P-Q \| \| - - vs. + + \| -44.49 \| -139.9 to 50.95 \| No \| ns \| 0.5048 \| P-R \| \| + - vs. + + \| 118.0 \| 22.52 to 213.4 \| Yes \| * \| 0.0118 \| Q-R \| |
| **Figure 4A. COX-2 mRNA level in the Cortex of C57BL/6N mice** |
| \| Number of families \| 1 \|  \|  \|  \|  \|  \| \| --- \| --- \| --- \| --- \| --- \| --- \| --- \| \| Number of comparisons per family \| 3 \|  \|  \|  \|  \|  \| \| Alpha \| 0.05 \|  \|  \|  \|  \|  \| \|  \|  \|  \|  \|  \|  \|  \| \| Tukey's multiple comparisons test \| Mean Diff. \| 95.00% CI of diff. \| Below threshold? \| Summary \| Adjusted P Value \|  \| \| - - vs. + - \| -3.164 \| -4.406 to -1.923 \| Yes \| **** \| <0.0001 \| A-B \| \| - - vs. + + \| -1.844 \| -3.185 to -0.5031 \| Yes \| ** \| 0.0066 \| A-C \| \| + - vs. + + \| 1.320 \| -0.02064 to 2.661 \| No \| ns \| 0.0541 \| B-C \| |
| **Figure 4B. COX-2 mRNA level in the Hippocampus of C57BL/6N mice** |
| \| Number of families \| 1 \|  \|  \|  \|  \|  \| \| --- \| --- \| --- \| --- \| --- \| --- \| --- \| \| Number of comparisons per family \| 3 \|  \|  \|  \|  \|  \| \| Alpha \| 0.05 \|  \|  \|  \|  \|  \| \|  \|  \|  \|  \|  \|  \|  \| \| Tukey's multiple comparisons test \| Mean Diff. \| 95.00% CI of diff. \| Below threshold? \| Summary \| Adjusted P Value \|  \| \| - - vs. + - \| -1.742 \| -2.558 to -0.9274 \| Yes \| **** \| <0.0001 \| A-B \| \| - - vs. + + \| -0.3100 \| -1.125 to 0.5051 \| No \| ns \| 0.6102 \| A-C \| \| + - vs. + + \| 1.432 \| 0.6174 to 2.248 \| Yes \| *** \| 0.0007 \| B-C \| |
| **Figure 4C. IL-1β mRNA level in the Cortex of C57BL/6N mice** |
| \| \| Number of families \| 1 \|  \|  \|  \|  \| \| --- \| --- \| --- \| --- \| --- \| --- \| \| Number of comparisons per family \| 3 \|  \|  \|  \|  \| \| Alpha \| 0.05 \|  \|  \|  \|  \| \|  \|  \|  \|  \|  \|  \| \| Newman-Keuls multiple comparisons test \| Mean Diff. \| Below threshold? \| Summary \|  \|  \| \| Vehicle vs. LPS \| -955.0 \| Yes \| *** \|  \| A-B \| \| Vehicle vs. L+sel \| -1193 \| Yes \| * \|  \| A-C \| \| LPS vs. L+sel \| -238.4 \| No \| ns \|  \| B-C \| \|  \|  \|  \|  \|  \|  \| \| --- \| --- \| --- \| --- \| --- \| --- \| --- \| --- \| --- \| --- \| --- \| --- \| --- \| --- \| --- \| --- \| --- \| --- \| --- \| --- \| --- \| --- \| --- \| --- \| --- \| --- \| --- \| --- \| --- \| --- \| --- \| --- \| --- \| --- \| --- \| --- \| --- \| --- \| --- \| --- \| --- \| --- \| --- \| --- \| --- \| --- \| --- \| --- \| --- \| --- \| --- \| --- \| --- \| --- \| --- \| |
| **Figure 4D. IL-1β mRNA level in the Hippocampus of C57BL/6N mice** |
| \| Number of families \| 1 \|  \|  \|  \|  \| \| --- \| --- \| --- \| --- \| --- \| --- \| \| Number of comparisons per family \| 3 \|  \|  \|  \|  \| \| Alpha \| 0.05 \|  \|  \|  \|  \| \|  \|  \|  \|  \|  \|  \| \| Newman-Keuls multiple comparisons test \| Mean Diff. \| Below threshold? \| Summary \|  \|  \| \| - - vs. + - \| -812.0 \| Yes \| ** \|  \| A-B \| \| - - vs. + + \| -285.9 \| No \| ns \|  \| A-C \| \| + - vs. + + \| 526.0 \| Yes \| * \|  \| B-C \| |
| **Figure 4E, F. COX-2 fluorescence intensity in the Hippocampal CA1 of C57BL/6N mice** |
| \| Number of families \| 1 \|  \|  \|  \|  \|  \| \| --- \| --- \| --- \| --- \| --- \| --- \| --- \| \| Number of comparisons per family \| 3 \|  \|  \|  \|  \|  \| \| Alpha \| 0.05 \|  \|  \|  \|  \|  \| \|  \|  \|  \|  \|  \|  \|  \| \| Tukey's multiple comparisons test \| Mean Diff. \| 95.00% CI of diff. \| Below threshold? \| Summary \| Adjusted P Value \|  \| \| - - vs. + - \| -45.24 \| -64.42 to -26.06 \| Yes \| **** \| <0.0001 \| A-B \| \| - - vs. + + \| -18.91 \| -38.09 to 0.2697 \| No \| ns \| 0.0541 \| A-C \| \| + - vs. + + \| 26.33 \| 7.400 to 45.26 \| Yes \| ** \| 0.0041 \| B-C \| |
| **Figure 4E, F. COX-2 fluorescence intensity in the Hippocampal CA2 of C57BL/6N mice** |
| \| Number of families \| 1 \|  \|  \|  \|  \|  \| \| --- \| --- \| --- \| --- \| --- \| --- \| --- \| \| Number of comparisons per family \| 3 \|  \|  \|  \|  \|  \| \| Alpha \| 0.05 \|  \|  \|  \|  \|  \| \|  \|  \|  \|  \|  \|  \|  \| \| Tukey's multiple comparisons test \| Mean Diff. \| 95.00% CI of diff. \| Below threshold? \| Summary \| Adjusted P Value \|  \| \| - - vs. + - \| -41.70 \| -64.34 to -19.07 \| Yes \| *** \| 0.0001 \| D-E \| \| - - vs. + + \| -22.33 \| -44.96 to 0.3052 \| No \| ns \| 0.0539 \| D-F \| \| + - vs. + + \| 19.38 \| -2.963 to 41.72 \| No \| ns \| 0.1014 \| E-F \| |
| **Figure 4E, F. COX-2 fluorescence intensity in the Hippocampal CA3 of C57BL/6N mice** |
| \| Number of families \| 1 \|  \|  \|  \|  \|  \| \| --- \| --- \| --- \| --- \| --- \| --- \| --- \| \| Number of comparisons per family \| 3 \|  \|  \|  \|  \|  \| \| Alpha \| 0.05 \|  \|  \|  \|  \|  \| \|  \|  \|  \|  \|  \|  \|  \| \| Tukey's multiple comparisons test \| Mean Diff. \| 95.00% CI of diff. \| Below threshold? \| Summary \| Adjusted P Value \|  \| \| - - vs. + - \| -43.51 \| -62.28 to -24.74 \| Yes \| **** \| <0.0001 \| G-H \| \| - - vs. + + \| -20.99 \| -39.76 to -2.219 \| Yes \| * \| 0.0249 \| G-I \| \| + - vs. + + \| 22.52 \| 3.989 to 41.05 \| Yes \| * \| 0.0135 \| H-I \| |
| **Figure 4E, F. COX-2 fluorescence intensity in the Hippocampal CA4 of C57BL/6N mice** |
| \| Number of families \| 1 \|  \|  \|  \|  \|  \| \| --- \| --- \| --- \| --- \| --- \| --- \| --- \| \| Number of comparisons per family \| 3 \|  \|  \|  \|  \|  \| \| Alpha \| 0.05 \|  \|  \|  \|  \|  \| \|  \|  \|  \|  \|  \|  \|  \| \| Tukey's multiple comparisons test \| Mean Diff. \| 95.00% CI of diff. \| Below threshold? \| Summary \| Adjusted P Value \|  \| \| - - vs. + - \| -63.42 \| -86.08 to -40.76 \| Yes \| **** \| <0.0001 \| J-K \| \| - - vs. + + \| -35.34 \| -58.00 to -12.68 \| Yes \| ** \| 0.0012 \| J-L \| \| + - vs. + + \| 28.08 \| 5.716 to 50.45 \| Yes \| * \| 0.0104 \| K-L \| |
| **Figure 4E, F. COX-2 fluorescence intensity in the Hippocampal DG of C57BL/6N mice** |
| \| Number of families \| 1 \|  \|  \|  \|  \|  \| \| --- \| --- \| --- \| --- \| --- \| --- \| --- \| \| Number of comparisons per family \| 3 \|  \|  \|  \|  \|  \| \| Alpha \| 0.05 \|  \|  \|  \|  \|  \| \|  \|  \|  \|  \|  \|  \|  \| \| Tukey's multiple comparisons test \| Mean Diff. \| 95.00% CI of diff. \| Below threshold? \| Summary \| Adjusted P Value \|  \| \| - - vs. + - \| -61.22 \| -81.39 to -41.06 \| Yes \| **** \| <0.0001 \| M-N \| \| - - vs. + + \| -31.52 \| -51.68 to -11.36 \| Yes \| ** \| 0.0012 \| M-O \| \| + - vs. + + \| 29.70 \| 9.800 to 49.60 \| Yes \| ** \| 0.0020 \| N-O \| |
| **Figure 4G, H. IL-1β fluorescence intensity in the Hippocampal CA1 of C57BL/6N mice** |
| \| Number of families \| 1 \|  \|  \|  \|  \|  \| \| --- \| --- \| --- \| --- \| --- \| --- \| --- \| \| Number of comparisons per family \| 3 \|  \|  \|  \|  \|  \| \| Alpha \| 0.05 \|  \|  \|  \|  \|  \| \|  \|  \|  \|  \|  \|  \|  \| \| Tukey's multiple comparisons test \| Mean Diff. \| 95.00% CI of diff. \| Below threshold? \| Summary \| Adjusted P Value \|  \| \| - - vs. + - \| -121.3 \| -165.3 to -77.23 \| Yes \| **** \| <0.0001 \| A-B \| \| - - vs. + + \| -36.12 \| -80.16 to 7.923 \| No \| ns \| 0.1281 \| A-C \| \| + - vs. + + \| 85.16 \| 41.12 to 129.2 \| Yes \| **** \| <0.0001 \| B-C \| |
| **Figure 4G, H. IL-1β fluorescence intensity in the Hippocampal CA2 of C57BL/6N mice** |
| \| Number of families \| 1 \|  \|  \|  \|  \|  \| \| --- \| --- \| --- \| --- \| --- \| --- \| --- \| \| Number of comparisons per family \| 3 \|  \|  \|  \|  \|  \| \| Alpha \| 0.05 \|  \|  \|  \|  \|  \| \|  \|  \|  \|  \|  \|  \|  \| \| Tukey's multiple comparisons test \| Mean Diff. \| 95.00% CI of diff. \| Below threshold? \| Summary \| Adjusted P Value \|  \| \| - - vs. + - \| -121.7 \| -173.7 to -69.71 \| Yes \| **** \| <0.0001 \| D-E \| \| - - vs. + + \| -42.48 \| -94.48 to 9.528 \| No \| ns \| 0.1301 \| D-F \| \| + - vs. + + \| 79.24 \| 27.24 to 131.2 \| Yes \| ** \| 0.0015 \| E-F \| |
| **Figure 4G, H. IL-1β fluorescence intensity in the Hippocampal CA3 of C57BL/6N mice** |
| \| Number of families \| 1 \|  \|  \|  \|  \|  \| \| --- \| --- \| --- \| --- \| --- \| --- \| --- \| \| Number of comparisons per family \| 3 \|  \|  \|  \|  \|  \| \| Alpha \| 0.05 \|  \|  \|  \|  \|  \| \|  \|  \|  \|  \|  \|  \|  \| \| Tukey's multiple comparisons test \| Mean Diff. \| 95.00% CI of diff. \| Below threshold? \| Summary \| Adjusted P Value \|  \| \| - - vs. + - \| -162.6 \| -228.5 to -96.63 \| Yes \| **** \| <0.0001 \| G-H \| \| - - vs. + + \| -80.50 \| -146.4 to -14.58 \| Yes \| * \| 0.0130 \| G-I \| \| + - vs. + + \| 82.05 \| 16.12 to 148.0 \| Yes \| * \| 0.0111 \| H-I \| |
| **Figure 4G, H. IL-1β fluorescence intensity in the Hippocampal CA4 of C57BL/6N mice** |
| \| Number of families \| 1 \|  \|  \|  \|  \|  \| \| --- \| --- \| --- \| --- \| --- \| --- \| --- \| \| Number of comparisons per family \| 3 \|  \|  \|  \|  \|  \| \| Alpha \| 0.05 \|  \|  \|  \|  \|  \| \|  \|  \|  \|  \|  \|  \|  \| \| Tukey's multiple comparisons test \| Mean Diff. \| 95.00% CI of diff. \| Below threshold? \| Summary \| Adjusted P Value \|  \| \| - - vs. + - \| -173.6 \| -223.0 to -124.3 \| Yes \| **** \| <0.0001 \| J-K \| \| - - vs. + + \| -55.08 \| -104.4 to -5.739 \| Yes \| * \| 0.0252 \| J-L \| \| + - vs. + + \| 118.6 \| 69.23 to 167.9 \| Yes \| **** \| <0.0001 \| K-L \| |
| **Figure 4G, H. IL-1β fluorescence intensity in the Hippocampal DG of C57BL/6N mice** |
| \| Number of families \| 1 \|  \|  \|  \|  \|  \| \| --- \| --- \| --- \| --- \| --- \| --- \| --- \| \| Number of comparisons per family \| 3 \|  \|  \|  \|  \|  \| \| Alpha \| 0.05 \|  \|  \|  \|  \|  \| \|  \|  \|  \|  \|  \|  \|  \| \| Tukey's multiple comparisons test \| Mean Diff. \| 95.00% CI of diff. \| Below threshold? \| Summary \| Adjusted P Value \|  \| \| - - vs. + - \| -137.2 \| -179.1 to -95.35 \| Yes \| **** \| <0.0001 \| M-N \| \| - - vs. + + \| -42.43 \| -84.29 to -0.5699 \| Yes \| * \| 0.0463 \| M-O \| \| + - vs. + + \| 94.78 \| 52.92 to 136.6 \| Yes \| **** \| <0.0001 \| N-O \| |
| **Figure 5A, C. TNF-α fluorescence intensity in the Cortex of C57BL/6N mice** |
| \| Number of families \| 1 \|  \|  \|  \|  \|  \| \| --- \| --- \| --- \| --- \| --- \| --- \| --- \| \| Number of comparisons per family \| 3 \|  \|  \|  \|  \|  \| \| Alpha \| 0.05 \|  \|  \|  \|  \|  \| \|  \|  \|  \|  \|  \|  \|  \| \| Tukey's multiple comparisons test \| Mean Diff. \| 95.00% CI of diff. \| Significant? \| Summary \| Adjusted P Value \|  \| \| - - vs. + - \| -31.01 \| -45.50 to -16.53 \| Yes \| **** \| <0.0001 \| A-B \| \| - - vs. + + \| -17.68 \| -32.16 to -3.190 \| Yes \| * \| 0.0131 \| A-C \| \| + - vs. + + \| 13.34 \| -1.151 to 27.82 \| No \| ns \| 0.0772 \| B-C \| |
| **Figure 5B, C. TNF-α fluorescence intensity in the Hippocampal CA1 of C57BL/6N mice** |
| \| Number of families \| 1 \|  \|  \|  \|  \|  \| \| --- \| --- \| --- \| --- \| --- \| --- \| --- \| \| Number of comparisons per family \| 3 \|  \|  \|  \|  \|  \| \| Alpha \| 0.05 \|  \|  \|  \|  \|  \| \|  \|  \|  \|  \|  \|  \|  \| \| Tukey's multiple comparisons test \| Mean Diff. \| 95.00% CI of diff. \| Below threshold? \| Summary \| Adjusted P Value \|  \| \| - - vs. + - \| -43.68 \| -63.18 to -24.18 \| Yes \| **** \| <0.0001 \| D-E \| \| - - vs. + + \| -23.72 \| -43.22 to -4.219 \| Yes \| * \| 0.0134 \| D-F \| \| + - vs. + + \| 19.96 \| 0.4635 to 39.46 \| Yes \| * \| 0.0437 \| E-F \| |
| **Figure 5B, C. TNF-α fluorescence intensity in the Hippocampal CA2 of C57BL/6N mice** |
| \| Number of families \| 1 \|  \|  \|  \|  \|  \| \| --- \| --- \| --- \| --- \| --- \| --- \| --- \| \| Number of comparisons per family \| 3 \|  \|  \|  \|  \|  \| \| Alpha \| 0.05 \|  \|  \|  \|  \|  \| \|  \|  \|  \|  \|  \|  \|  \| \| Tukey's multiple comparisons test \| Mean Diff. \| 95.00% CI of diff. \| Below threshold? \| Summary \| Adjusted P Value \|  \| \| - - vs. + - \| -41.78 \| -60.41 to -23.16 \| Yes \| **** \| <0.0001 \| G-H \| \| - - vs. + + \| -25.69 \| -44.08 to -7.312 \| Yes \| ** \| 0.0039 \| G-I \| \| + - vs. + + \| 16.09 \| -2.531 to 34.71 \| No \| ns \| 0.1032 \| H-I \| |
| **Figure 5B, C. TNF-α fluorescence intensity in the Hippocampal CA3 of C57BL/6N mice** |
| \| Number of families \| 1 \|  \|  \|  \|  \|  \| \| --- \| --- \| --- \| --- \| --- \| --- \| --- \| \| Number of comparisons per family \| 3 \|  \|  \|  \|  \|  \| \| Alpha \| 0.05 \|  \|  \|  \|  \|  \| \|  \|  \|  \|  \|  \|  \|  \| \| Tukey's multiple comparisons test \| Mean Diff. \| 95.00% CI of diff. \| Below threshold? \| Summary \| Adjusted P Value \|  \| \| - - vs. + - \| -52.25 \| -72.74 to -31.77 \| Yes \| **** \| <0.0001 \| J-K \| \| - - vs. + + \| -33.46 \| -53.94 to -12.97 \| Yes \| *** \| 0.0007 \| J-L \| \| + - vs. + + \| 18.79 \| -1.692 to 39.28 \| No \| ns \| 0.0785 \| K-L \| |
| **Figure 5B, C. TNF-α fluorescence intensity in the Hippocampal CA4 of C57BL/6N mice** |
| \| Number of families \| 1 \|  \|  \|  \|  \|  \| \| --- \| --- \| --- \| --- \| --- \| --- \| --- \| \| Number of comparisons per family \| 3 \|  \|  \|  \|  \|  \| \| Alpha \| 0.05 \|  \|  \|  \|  \|  \| \|  \|  \|  \|  \|  \|  \|  \| \| Tukey's multiple comparisons test \| Mean Diff. \| 95.00% CI of diff. \| Below threshold? \| Summary \| Adjusted P Value \|  \| \| - - vs. + - \| -45.80 \| -67.79 to -23.80 \| Yes \| **** \| <0.0001 \| M-N \| \| - - vs. + + \| -29.40 \| -51.40 to -7.400 \| Yes \| ** \| 0.0060 \| M-O \| \| + - vs. + + \| 16.40 \| -5.602 to 38.40 \| No \| ns \| 0.1808 \| N-O \| |
| **Figure 5B, C. TNF-α fluorescence intensity in the Hippocampal DG of C57BL/6N mice** |
| \| Number of families \| 1 \|  \|  \|  \|  \|  \| \| --- \| --- \| --- \| --- \| --- \| --- \| --- \| \| Number of comparisons per family \| 3 \|  \|  \|  \|  \|  \| \| Alpha \| 0.05 \|  \|  \|  \|  \|  \| \|  \|  \|  \|  \|  \|  \|  \| \| Tukey's multiple comparisons test \| Mean Diff. \| 95.00% CI of diff. \| Below threshold? \| Summary \| Adjusted P Value \|  \| \| - - vs. + - \| -44.21 \| -65.54 to -22.87 \| Yes \| **** \| <0.0001 \| P-Q \| \| - - vs. + + \| -25.32 \| -46.65 to -3.987 \| Yes \| * \| 0.0162 \| P-R \| \| + - vs. + + \| 18.89 \| -2.446 to 40.22 \| No \| ns \| 0.0927 \| Q-R \| |
| **Figure 5D. TNF-α mRNA level in the Cortex of C57BL/6N mice** |
| \| Number of families \| 1 \|  \|  \|  \|  \|  \| \| --- \| --- \| --- \| --- \| --- \| --- \| --- \| \| Number of comparisons per family \| 3 \|  \|  \|  \|  \|  \| \| Alpha \| 0.05 \|  \|  \|  \|  \|  \| \|  \|  \|  \|  \|  \|  \|  \| \| Tukey's multiple comparisons test \| Mean Diff. \| 95.00% CI of diff. \| Below threshold? \| Summary \| Adjusted P Value \|  \| \| - - vs. + - \| -124.2 \| -182.7 to -65.73 \| Yes \| **** \| <0.0001 \| A-B \| \| - - vs. + + \| -105.8 \| -166.4 to -45.28 \| Yes \| *** \| 0.0007 \| A-C \| \| + - vs. + + \| 18.40 \| -42.14 to 78.94 \| No \| ns \| 0.7259 \| B-C \| |
| **Figure 5D. TNF-α mRNA level in the Hippocampus of C57BL/6N mice** |
| \| Number of families \| 1 \|  \|  \|  \|  \|  \| \| --- \| --- \| --- \| --- \| --- \| --- \| --- \| \| Number of comparisons per family \| 3 \|  \|  \|  \|  \|  \| \| Alpha \| 0.05 \|  \|  \|  \|  \|  \| \|  \|  \|  \|  \|  \|  \|  \| \| Tukey's multiple comparisons test \| Mean Diff. \| 95.00% CI of diff. \| Below threshold? \| Summary \| Adjusted P Value \|  \| \| - - vs. + - \| -211.5 \| -318.4 to -104.6 \| Yes \| *** \| 0.0002 \| D-E \| \| - - vs. + + \| -100.8 \| -207.7 to 6.092 \| No \| ns \| 0.0669 \| D-F \| \| + - vs. + + \| 110.7 \| 3.832 to 217.6 \| Yes \| * \| 0.0415 \| E-F \| |
| **Figure 6A. CXCL10 mRNA level in the Cortex of C57BL/6N mice** |
| \| Number of families \| 1 \|  \|  \|  \|  \|  \| \| --- \| --- \| --- \| --- \| --- \| --- \| --- \| \| Number of comparisons per family \| 3 \|  \|  \|  \|  \|  \| \| Alpha \| 0.05 \|  \|  \|  \|  \|  \| \|  \|  \|  \|  \|  \|  \|  \| \| Tukey's multiple comparisons test \| Mean Diff. \| 95.00% CI of diff. \| Below threshold? \| Summary \| Adjusted P Value \|  \| \| - - vs. + - \| -1789 \| -2276 to -1303 \| Yes \| **** \| <0.0001 \| A-B \| \| - - vs. + + \| -1151 \| -1637 to -664.0 \| Yes \| **** \| <0.0001 \| A-C \| \| + - vs. + + \| 638.7 \| 152.2 to 1125 \| Yes \| ** \| 0.0090 \| B-C \| |
| **Figure 6A. CXCL10 mRNA level in the Hippocampus of C57BL/6N mice** |
| \| Number of families \| 1 \|  \|  \|  \|  \|  \| \| --- \| --- \| --- \| --- \| --- \| --- \| --- \| \| Number of comparisons per family \| 3 \|  \|  \|  \|  \|  \| \| Alpha \| 0.05 \|  \|  \|  \|  \|  \| \|  \|  \|  \|  \|  \|  \|  \| \| Tukey's multiple comparisons test \| Mean Diff. \| 95.00% CI of diff. \| Below threshold? \| Summary \| Adjusted P Value \|  \| \| - - vs. + - \| -513.1 \| -946.9 to -79.31 \| Yes \| * \| 0.0189 \| D-E \| \| - - vs. + + \| -61.71 \| -495.5 to 372.1 \| No \| ns \| 0.9313 \| D-F \| \| + - vs. + + \| 451.4 \| 32.31 to 870.5 \| Yes \| * \| 0.0334 \| E-F \| |
| **Figure 6B. SERPINA3N mRNA level in the Cortex of C57BL/6N mice** |
| \| Number of families \| 1 \|  \|  \|  \|  \|  \| \| --- \| --- \| --- \| --- \| --- \| --- \| --- \| \| Number of comparisons per family \| 3 \|  \|  \|  \|  \|  \| \| Alpha \| 0.05 \|  \|  \|  \|  \|  \| \|  \|  \|  \|  \|  \|  \|  \| \| Tukey's multiple comparisons test \| Mean Diff. \| 95.00% CI of diff. \| Below threshold? \| Summary \| Adjusted P Value \|  \| \| - - vs. + - \| -30.01 \| -35.48 to -24.54 \| Yes \| **** \| <0.0001 \| A-B \| \| - - vs. + + \| -16.85 \| -22.32 to -11.38 \| Yes \| **** \| <0.0001 \| A-C \| \| + - vs. + + \| 13.16 \| 7.693 to 18.63 \| Yes \| **** \| <0.0001 \| B-C \| |
| **Figure 6B. SERPINA3N mRNA level in the Hippocampus of C57BL/6N mice** |
| \| Number of families \| 1 \|  \|  \|  \|  \|  \| \| --- \| --- \| --- \| --- \| --- \| --- \| --- \| \| Number of comparisons per family \| 3 \|  \|  \|  \|  \|  \| \| Alpha \| 0.05 \|  \|  \|  \|  \|  \| \|  \|  \|  \|  \|  \|  \|  \| \| Tukey's multiple comparisons test \| Mean Diff. \| 95.00% CI of diff. \| Below threshold? \| Summary \| Adjusted P Value \|  \| \| - - vs. + - \| -4.032 \| -6.292 to -1.772 \| Yes \| *** \| 0.0006 \| D-E \| \| - - vs. + + \| -2.823 \| -5.083 to -0.5631 \| Yes \| * \| 0.0131 \| D-F \| \| + - vs. + + \| 1.209 \| -0.9745 to 3.392 \| No \| ns \| 0.3595 \| E-F \| |
| **Figure 6C. GBP2 mRNA level in the Cortex of C57BL/6N mice** |
| \| Number of families \| 1 \|  \|  \|  \|  \|  \| \| --- \| --- \| --- \| --- \| --- \| --- \| --- \| \| Number of comparisons per family \| 3 \|  \|  \|  \|  \|  \| \| Alpha \| 0.05 \|  \|  \|  \|  \|  \| \|  \|  \|  \|  \|  \|  \|  \| \| Tukey's multiple comparisons test \| Mean Diff. \| 95.00% CI of diff. \| Below threshold? \| Summary \| Adjusted P Value \|  \| \| - - vs. + - \| -80.20 \| -119.2 to -41.20 \| Yes \| *** \| 0.0001 \| A-B \| \| - - vs. + + \| -55.93 \| -94.94 to -16.93 \| Yes \| ** \| 0.0044 \| A-C \| \| + - vs. + + \| 24.27 \| -14.74 to 63.27 \| No \| ns \| 0.2810 \| B-C \| |
| **Figure 6C. GBP2 mRNA level in the Hippocampus of C57BL/6N mice** |
| \| Number of families \| 1 \|  \|  \|  \|  \|  \| \| --- \| --- \| --- \| --- \| --- \| --- \| --- \| \| Number of comparisons per family \| 3 \|  \|  \|  \|  \|  \| \| Alpha \| 0.05 \|  \|  \|  \|  \|  \| \|  \|  \|  \|  \|  \|  \|  \| \| Tukey's multiple comparisons test \| Mean Diff. \| 95.00% CI of diff. \| Below threshold? \| Summary \| Adjusted P Value \|  \| \| - - vs. + - \| -23.92 \| -28.20 to -19.64 \| Yes \| **** \| <0.0001 \| D-E \| \| - - vs. + + \| -19.45 \| -23.73 to -15.17 \| Yes \| **** \| <0.0001 \| D-F \| \| + - vs. + + \| 4.471 \| 0.1941 to 8.747 \| Yes \| * \| 0.0394 \| E-F \| |
| **Figure 6D. CHI3L1 mRNA level in the Cortex of C57BL/6N mice** |
| \| Number of families \| 1 \|  \|  \|  \|  \|  \| \| --- \| --- \| --- \| --- \| --- \| --- \| --- \| \| Number of comparisons per family \| 3 \|  \|  \|  \|  \|  \| \| Alpha \| 0.05 \|  \|  \|  \|  \|  \| \|  \|  \|  \|  \|  \|  \|  \| \| Tukey's multiple comparisons test \| Mean Diff. \| 95.00% CI of diff. \| Below threshold? \| Summary \| Adjusted P Value \|  \| \| - - vs. + - \| -1.072 \| -1.824 to -0.3203 \| Yes \| ** \| 0.0047 \| A-B \| \| - - vs. + + \| -0.6531 \| -1.405 to 0.09889 \| No \| ns \| 0.0964 \| A-C \| \| + - vs. + + \| 0.4192 \| -0.3327 to 1.171 \| No \| ns \| 0.3563 \| B-C \| |
| **Figure 6D. CHI3L1 mRNA level in the Hippocampus of C57BL/6N mice** |
| \| Number of families \| 1 \|  \|  \|  \|  \|  \| \| --- \| --- \| --- \| --- \| --- \| --- \| --- \| \| Number of comparisons per family \| 3 \|  \|  \|  \|  \|  \| \| Alpha \| 0.05 \|  \|  \|  \|  \|  \| \|  \|  \|  \|  \|  \|  \|  \| \| Tukey's multiple comparisons test \| Mean Diff. \| 95.00% CI of diff. \| Below threshold? \| Summary \| Adjusted P Value \|  \| \| - - vs. + - \| -7.672 \| -10.07 to -5.270 \| Yes \| **** \| <0.0001 \| D-E \| \| - - vs. + + \| -5.008 \| -7.410 to -2.606 \| Yes \| **** \| <0.0001 \| D-F \| \| + - vs. + + \| 2.664 \| 0.2624 to 5.066 \| Yes \| * \| 0.0280 \| E-F \| |
| **Figure 6E. CD44 mRNA level in the Cortex of C57BL/6N mice** |
| \| Number of families \| 1 \|  \|  \|  \|  \|  \| \| --- \| --- \| --- \| --- \| --- \| --- \| --- \| \| Number of comparisons per family \| 3 \|  \|  \|  \|  \|  \| \| Alpha \| 0.05 \|  \|  \|  \|  \|  \| \|  \|  \|  \|  \|  \|  \|  \| \| Tukey's multiple comparisons test \| Mean Diff. \| 95.00% CI of diff. \| Below threshold? \| Summary \| Adjusted P Value \|  \| \| - - vs. + - \| -11.43 \| -15.37 to -7.498 \| Yes \| **** \| <0.0001 \| A-B \| \| - - vs. + + \| -7.151 \| -11.09 to -3.215 \| Yes \| *** \| 0.0005 \| A-C \| \| + - vs. + + \| 4.283 \| 0.3467 to 8.218 \| Yes \| * \| 0.0314 \| B-C \| |
| **Figure 6E. CD44 mRNA level in the Hippocampus of C57BL/6N mice** |
| \| Number of families \| 1 \|  \|  \|  \|  \| \| --- \| --- \| --- \| --- \| --- \| --- \| \| Number of comparisons per family \| 3 \|  \|  \|  \|  \| \| Alpha \| 0.05 \|  \|  \|  \|  \| \|  \|  \|  \|  \|  \|  \| \| Newman-Keuls multiple comparisons test \| Mean Diff. \| Below threshold? \| Summary \|  \|  \| \| Column D vs. Column E \| -8.537 \| Yes \| *** \|  \| D-E \| \| Column D vs. Column F \| -4.191 \| Yes \| * \|  \| D-F \| \| Column E vs. Column F \| 4.346 \| Yes \| * \|  \| E-F \| |
| **Figure 6F. P2RY12 mRNA level in the Cortex of C57BL/6N mice** |
| \| Number of families \| 1 \|  \|  \|  \|  \|  \| \| --- \| --- \| --- \| --- \| --- \| --- \| --- \| \| Number of comparisons per family \| 3 \|  \|  \|  \|  \|  \| \| Alpha \| 0.05 \|  \|  \|  \|  \|  \| \|  \|  \|  \|  \|  \|  \|  \| \| Tukey's multiple comparisons test \| Mean Diff. \| 95.00% CI of diff. \| Below threshold? \| Summary \| Adjusted P Value \|  \| \| - - vs. + - \| 0.9728 \| 0.8996 to 1.046 \| Yes \| **** \| <0.0001 \| A-B \| \| - - vs. + + \| 0.9362 \| 0.8605 to 1.012 \| Yes \| **** \| <0.0001 \| A-C \| \| + - vs. + + \| -0.03663 \| -0.1123 to 0.03910 \| No \| ns \| 0.4534 \| B-C \| |
| **Figure 6F. P2RY12 mRNA level in the Hippocampus of C57BL/6N mice** |
| \| Number of families \| 1 \|  \|  \|  \|  \|  \| \| --- \| --- \| --- \| --- \| --- \| --- \| --- \| \| Number of comparisons per family \| 3 \|  \|  \|  \|  \|  \| \| Alpha \| 0.05 \|  \|  \|  \|  \|  \| \|  \|  \|  \|  \|  \|  \|  \| \| Tukey's multiple comparisons test \| Mean Diff. \| 95.00% CI of diff. \| Below threshold? \| Summary \| Adjusted P Value \|  \| \| - - vs. + - \| 0.9031 \| 0.6861 to 1.120 \| Yes \| **** \| <0.0001 \| D-E \| \| - - vs. + + \| 0.7783 \| 0.5612 to 0.9953 \| Yes \| **** \| <0.0001 \| D-F \| \| + - vs. + + \| -0.1249 \| -0.3345 to 0.08484 \| No \| ns \| 0.3092 \| E-F \| |
| **Figure 7A. NLRP3 mRNA level in the Cortex of C57BL/6N mice** |
| \| Number of families \| 1 \|  \|  \|  \|  \|  \| \| --- \| --- \| --- \| --- \| --- \| --- \| --- \| \| Number of comparisons per family \| 3 \|  \|  \|  \|  \|  \| \| Alpha \| 0.05 \|  \|  \|  \|  \|  \| \|  \|  \|  \|  \|  \|  \|  \| \| Tukey's multiple comparisons test \| Mean diff. \| 95.00% CI of diff. \| Below threshold? \| Summary \| Adjusted P Value \|  \| \| - - vs. + - \| -29.96 \| -38.02 to -21.90 \| Yes \| **** \| <0.0001 \| A-B \| \| - - vs. + + \| -18.61 \| -26.67 to -10.55 \| Yes \| **** \| <0.0001 \| A-C \| \| + - vs. + + \| 11.36 \| 3.297 to 19.42 \| Yes \| ** \| 0.0051 \| B-C \| |
| **Figure 7A. NLRP3 mRNA level in the Hippocampus of C57BL/6N mice** |
| \| Number of families \| 1 \|  \|  \|  \|  \|  \| \| --- \| --- \| --- \| --- \| --- \| --- \| --- \| \| Number of comparisons per family \| 3 \|  \|  \|  \|  \|  \| \| Alpha \| 0.05 \|  \|  \|  \|  \|  \| \|  \|  \|  \|  \|  \|  \|  \| \| Tukey's multiple comparisons test \| Mean Diff. \| 95.00% CI of diff. \| Below threshold? \| Summary \| Adjusted P Value \|  \| \| - - vs. + - \| -6.351 \| -9.001 to -3.702 \| Yes \| **** \| <0.0001 \| D-E \| \| - - vs. + + \| -3.207 \| -5.857 to -0.5580 \| Yes \| * \| 0.0160 \| D-F \| \| + - vs. + + \| 3.144 \| 0.4945 to 5.793 \| Yes \| * \| 0.0183 \| E-F \| |
| **Figure 7B. pro-IL-1β mRNA level in the Cortex of C57BL/6N mice** |
| \| Number of families \| 1 \|  \|  \|  \|  \|  \| \| --- \| --- \| --- \| --- \| --- \| --- \| --- \| \| Number of comparisons per family \| 3 \|  \|  \|  \|  \|  \| \| Alpha \| 0.05 \|  \|  \|  \|  \|  \| \|  \|  \|  \|  \|  \|  \|  \| \| Tukey's multiple comparisons test \| Mean diff. \| 95.00% CI of diff. \| Below threshold? \| Summary \| Adjusted P Value \|  \| \| - - vs. + - \| -1179 \| -1682 to -675.3 \| Yes \| **** \| <0.0001 \| A-B \| \| - - vs. + + \| -772.5 \| -1276 to -269.2 \| Yes \| ** \| 0.0025 \| A-C \| \| + - vs. + + \| 406.1 \| -97.18 to 909.4 \| No \| ns \| 0.1288 \| B-C \| |
| **Figure 7B. pro-IL-1β mRNA level in the Hippocampus of C57BL/6N mice** |
| \| Number of families \| 1 \|  \|  \|  \|  \|  \| \| --- \| --- \| --- \| --- \| --- \| --- \| --- \| \| Number of comparisons per family \| 3 \|  \|  \|  \|  \|  \| \| Alpha \| 0.05 \|  \|  \|  \|  \|  \| \|  \|  \|  \|  \|  \|  \|  \| \| Tukey's multiple comparisons test \| Mean Diff. \| 95.00% CI of diff. \| Below threshold? \| Summary \| Adjusted P Value \|  \| \| - - vs. + - \| -394.9 \| -627.0 to -162.7 \| Yes \| *** \| 0.0009 \| D-E \| \| - - vs. + + \| -127.2 \| -359.3 to 105.0 \| No \| ns \| 0.3685 \| D-F \| \| + - vs. + + \| 267.7 \| 35.55 to 499.8 \| Yes \| * \| 0.0220 \| E-F \| |
| **Figure 7C. BV2 (5 μM Selexipag, 6 hr), NLRP3 mRNA level** |
| \| Number of families \| 1 \|  \|  \|  \|  \|  \| \| --- \| --- \| --- \| --- \| --- \| --- \| --- \| \| Number of comparisons per family \| 3 \|  \|  \|  \|  \|  \| \| Alpha \| 0.05 \|  \|  \|  \|  \|  \| \|  \|  \|  \|  \|  \|  \|  \| \| Tukey's multiple comparisons test \| Mean Diff. \| 95.00% CI of diff. \| Below threshold? \| Summary \| Adjusted P Value \|  \| \| - - vs. + - \| -10.56 \| -12.60 to -8.526 \| Yes \| **** \| <0.0001 \| A-B \| \| - - vs. + + \| -5.378 \| -7.416 to -3.340 \| Yes \| *** \| 0.0001 \| A-C \| \| + - vs. + + \| 5.187 \| 3.149 to 7.225 \| Yes \| *** \| 0.0001 \| B-C \| |
| **Figure 7D, E. BV2 (5 μM Selexipag, 24 hr), NLRP3 protein level** |
| \| Number of families \| 1 \|  \|  \|  \|  \|  \| \| --- \| --- \| --- \| --- \| --- \| --- \| --- \| \| Number of comparisons per family \| 3 \|  \|  \|  \|  \|  \| \| Alpha \| 0.05 \|  \|  \|  \|  \|  \| \|  \|  \|  \|  \|  \|  \|  \| \| Tukey's multiple comparisons test \| Mean diff. \| 95.00% CI of diff. \| Below threshold? \| Summary \| Adjusted P Value \|  \| \| Column A vs. Column B \| -2375 \| -3040 to -1710 \| Yes \| **** \| <0.0001 \| A-B \| \| Column A vs. Column C \| -1658 \| -2323 to -993.5 \| Yes \| **** \| <0.0001 \| A-C \| \| Column B vs. Column C \| 716.7 \| 51.75 to 1382 \| Yes \| * \| 0.0329 \| B-C \| |
| **Figure 7F. BV2 (5 μM Selexipag, 6 hr), CASPASE-1 mRNA level** |
| \| Number of families \| 1 \|  \|  \|  \|  \| \| --- \| --- \| --- \| --- \| --- \| --- \| \| Number of comparisons per family \| 3 \|  \|  \|  \|  \| \| Alpha \| 0.05 \|  \|  \|  \|  \| \|  \|  \|  \|  \|  \|  \| \| Newman-Keuls multiple comparisons test \| Mean Diff. \| Below threshold? \| Summary \|  \|  \| \| - - vs. + - \| -1.449 \| Yes \| **** \|  \| A-B \| \| - - vs. + + \| -1.151 \| Yes \| **** \|  \| A-C \| \| + - vs. + + \| 0.2986 \| Yes \| * \|  \| B-C \| |
| **Figure 7G. BV2 (5 μM Selexipag, 6 hr), pro-IL-1β mRNA level** |
| \| Number of families \| 1 \|  \|  \|  \|  \|  \| \| --- \| --- \| --- \| --- \| --- \| --- \| --- \| \| Number of comparisons per family \| 3 \|  \|  \|  \|  \|  \| \| Alpha \| 0.05 \|  \|  \|  \|  \|  \| \|  \|  \|  \|  \|  \|  \|  \| \| Tukey's multiple comparisons test \| Mean Diff. \| 95.00% CI of diff. \| Below threshold? \| Summary \| Adjusted P Value \|  \| \| Column A vs. Column B \| -176.9 \| -256.1 to -97.72 \| Yes \| **** \| <0.0001 \| A-B \| \| Column A vs. Column C \| -40.21 \| -119.4 to 39.00 \| No \| ns \| 0.4218 \| A-C \| \| Column B vs. Column C \| 136.7 \| 57.50 to 215.9 \| Yes \| *** \| 0.0008 \| B-C \| |
| **Figure 7H. PMC (5 μM Selexipag, 6 hr), NLRP3 mRNA level** |
| \| Number of families \| 1 \|  \|  \|  \|  \|  \| \| --- \| --- \| --- \| --- \| --- \| --- \| --- \| \| Number of comparisons per family \| 3 \|  \|  \|  \|  \|  \| \| Alpha \| 0.05 \|  \|  \|  \|  \|  \| \|  \|  \|  \|  \|  \|  \|  \| \| Tukey's multiple comparisons test \| Mean diff. \| 95.00% CI of diff. \| Below threshold? \| Summary \| Adjusted P Value \|  \| \| - - vs. + - \| -32.15 \| -39.30 to -24.99 \| Yes \| **** \| <0.0001 \| A-B \| \| - - vs. + + \| -15.78 \| -22.94 to -8.623 \| Yes \| **** \| <0.0001 \| A-C \| \| + - vs. + + \| 16.36 \| 9.206 to 23.52 \| Yes \| **** \| <0.0001 \| B-C \| |
| **Figure 7I. PMC (5 μM Selexipag, 6 hr), CASPASE-1 mRNA level** |
| \| Number of families \| 1 \|  \|  \|  \|  \|  \| \| --- \| --- \| --- \| --- \| --- \| --- \| --- \| \| Number of comparisons per family \| 3 \|  \|  \|  \|  \|  \| \| Alpha \| 0.05 \|  \|  \|  \|  \|  \| \|  \|  \|  \|  \|  \|  \|  \| \| Tukey's multiple comparisons test \| Mean diff. \| 95.00% CI of diff. \| Below threshold? \| Summary \| Adjusted P Value \|  \| \| - - vs. + - \| -3.201 \| -4.051 to -2.351 \| Yes \| **** \| <0.0001 \| A-B \| \| - - vs. + + \| -1.835 \| -2.685 to -0.9851 \| Yes \| **** \| <0.0001 \| A-C \| \| + - vs. + + \| 1.366 \| 0.5160 to 2.216 \| Yes \| ** \| 0.0016 \| B-C \| |
| **Figure 7J. PMC (5 μM Selexipag, 6 hr), pro-IL-1β mRNA level** |
| \| Number of families \| 1 \|  \|  \|  \|  \|  \| \| --- \| --- \| --- \| --- \| --- \| --- \| --- \| \| Number of comparisons per family \| 3 \|  \|  \|  \|  \|  \| \| Alpha \| 0.05 \|  \|  \|  \|  \|  \| \|  \|  \|  \|  \|  \|  \|  \| \| Tukey's multiple comparisons test \| Mean diff. \| 95.00% CI of diff. \| Below threshold? \| Summary \| Adjusted P Value \|  \| \| - - vs. + - \| -94.35 \| -113.6 to -75.09 \| Yes \| **** \| <0.0001 \| A-B \| \| - - vs. + + \| -51.84 \| -71.11 to -32.57 \| Yes \| **** \| <0.0001 \| A-C \| \| + - vs. + + \| 42.51 \| 23.25 to 61.78 \| Yes \| **** \| <0.0001 \| B-C \| |
| **Figure 8A. BV2 (NLRP3 siRNA transfected), NLRP3 mRNA level** |
| \| Number of families \| 1 \|  \|  \|  \|  \|  \| \| --- \| --- \| --- \| --- \| --- \| --- \| --- \| \| Number of comparisons per family \| 15 \|  \|  \|  \|  \|  \| \| Alpha \| 0.05 \|  \|  \|  \|  \|  \| \|  \|  \|  \|  \|  \|  \|  \| \| Tukey's multiple comparisons test \| Mean Diff. \| 95.00% CI of diff. \| Below threshold? \| Summary \| Adjusted P Value \|  \| \| - - vs. + - \| -6.036 \| -8.280 to -3.792 \| Yes \| **** \| <0.0001 \| A-B \| \| - - vs. + + \| -1.646 \| -3.890 to 0.5982 \| No \| ns \| 0.2635 \| A-C \| \| - - vs. Column D \| 0.8637 \| -1.380 to 3.108 \| No \| ns \| 0.8572 \| A-D \| \| - - vs. Column E \| -0.6376 \| -2.882 to 1.606 \| No \| ns \| 0.9562 \| A-E \| \| - - vs. Column F \| 0.3609 \| -1.962 to 2.684 \| No \| ns \| 0.9971 \| A-F \| \| + - vs. + + \| 4.390 \| 2.146 to 6.634 \| Yes \| **** \| <0.0001 \| B-C \| \| + - vs. Column D \| 6.900 \| 4.655 to 9.144 \| Yes \| **** \| <0.0001 \| B-D \| \| + - vs. Column E \| 5.398 \| 3.154 to 7.642 \| Yes \| **** \| <0.0001 \| B-E \| \| + - vs. Column F \| 6.397 \| 4.074 to 8.720 \| Yes \| **** \| <0.0001 \| B-F \| \| + + vs. Column D \| 2.510 \| 0.2655 to 4.754 \| Yes \| * \| 0.0205 \| C-D \| \| + + vs. Column E \| 1.008 \| -1.236 to 3.252 \| No \| ns \| 0.7597 \| C-E \| \| + + vs. Column F \| 2.007 \| -0.3161 to 4.330 \| No \| ns \| 0.1249 \| C-F \| \| Column D vs. Column E \| -1.501 \| -3.745 to 0.7427 \| No \| ns \| 0.3602 \| D-E \| \| Column D vs. Column F \| -0.5028 \| -2.826 to 1.820 \| No \| ns \| 0.9866 \| D-F \| \| Column E vs. Column F \| 0.9986 \| -1.324 to 3.321 \| No \| ns \| 0.7915 \| E-F \| |
| **Figure 8B. BV2 (NLRP3 siRNA transfected), COX-2 mRNA level** |
| \| Number of families \| 1 \|  \|  \|  \|  \|  \| \| --- \| --- \| --- \| --- \| --- \| --- \| --- \| \| Number of comparisons per family \| 15 \|  \|  \|  \|  \|  \| \| Alpha \| 0.05 \|  \|  \|  \|  \|  \| \|  \|  \|  \|  \|  \|  \|  \| \| Tukey's multiple comparisons test \| Mean Diff. \| 95.00% CI of diff. \| Below threshold? \| Summary \| Adjusted P Value \|  \| \| - - vs. + - \| -12.12 \| -18.39 to -5.853 \| Yes \| **** \| <0.0001 \| A-B \| \| - - vs. + + \| -0.5010 \| -6.771 to 5.769 \| No \| ns \| 0.9999 \| A-C \| \| - - vs. Column D \| -0.6192 \| -6.889 to 5.651 \| No \| ns \| 0.9997 \| A-D \| \| - - vs. Column E \| -4.249 \| -10.52 to 2.021 \| No \| ns \| 0.3462 \| A-E \| \| - - vs. Column F \| -6.860 \| -13.35 to -0.3695 \| Yes \| * \| 0.0329 \| A-F \| \| + - vs. + + \| 11.62 \| 5.352 to 17.89 \| Yes \| **** \| <0.0001 \| B-C \| \| + - vs. Column D \| 11.50 \| 5.234 to 17.77 \| Yes \| **** \| <0.0001 \| B-D \| \| + - vs. Column E \| 7.874 \| 1.604 to 14.14 \| Yes \| ** \| 0.0067 \| B-E \| \| + - vs. Column F \| 5.263 \| -1.227 to 11.75 \| No \| ns \| 0.1721 \| B-F \| \| + + vs. Column D \| -0.1182 \| -6.388 to 6.152 \| No \| ns \| >0.9999 \| C-D \| \| + + vs. Column E \| -3.748 \| -10.02 to 2.522 \| No \| ns \| 0.4853 \| C-E \| \| + + vs. Column F \| -6.359 \| -12.85 to 0.1315 \| No \| ns \| 0.0578 \| C-F \| \| Column D vs. Column E \| -3.630 \| -9.900 to 2.640 \| No \| ns \| 0.5206 \| D-E \| \| Column D vs. Column F \| -6.241 \| -12.73 to 0.2497 \| No \| ns \| 0.0656 \| D-F \| \| Column E vs. Column F \| -2.611 \| -9.101 to 3.880 \| No \| ns \| 0.8332 \| E-F \| |
| **Figure 8C. BV2 (NLRP3 siRNA transfected), IL-1β mRNA level** |
| \| Number of families \| 1 \|  \|  \|  \|  \|  \| \| --- \| --- \| --- \| --- \| --- \| --- \| --- \| \| Number of comparisons per family \| 15 \|  \|  \|  \|  \|  \| \| Alpha \| 0.05 \|  \|  \|  \|  \|  \| \|  \|  \|  \|  \|  \|  \|  \| \| Tukey's multiple comparisons test \| Mean Diff. \| 95.00% CI of diff. \| Below threshold? \| Summary \| Adjusted P Value \|  \| \| - - vs. + - \| -294.2 \| -416.8 to -171.6 \| Yes \| **** \| <0.0001 \| A-B \| \| - - vs. + + \| -11.32 \| -133.9 to 111.3 \| No \| ns \| 0.9998 \| A-C \| \| - - vs. Column D \| -0.008073 \| -122.6 to 122.6 \| No \| ns \| >0.9999 \| A-D \| \| - - vs. Column E \| -67.25 \| -189.9 to 55.36 \| No \| ns \| 0.5782 \| A-E \| \| - - vs. Column F \| -29.11 \| -156.0 to 97.81 \| No \| ns \| 0.9826 \| A-F \| \| + - vs. + + \| 282.9 \| 160.2 to 405.5 \| Yes \| **** \| <0.0001 \| B-C \| \| + - vs. Column D \| 294.2 \| 171.6 to 416.8 \| Yes \| **** \| <0.0001 \| B-D \| \| + - vs. Column E \| 226.9 \| 104.3 to 349.5 \| Yes \| **** \| <0.0001 \| B-E \| \| + - vs. Column F \| 265.1 \| 138.2 to 392.0 \| Yes \| **** \| <0.0001 \| B-F \| \| + + vs. Column D \| 11.31 \| -111.3 to 133.9 \| No \| ns \| 0.9998 \| C-D \| \| + + vs. Column E \| -55.94 \| -178.6 to 66.68 \| No \| ns \| 0.7480 \| C-E \| \| + + vs. Column F \| -17.79 \| -144.7 to 109.1 \| No \| ns \| 0.9982 \| C-F \| \| Column D vs. Column E \| -67.25 \| -189.9 to 55.37 \| No \| ns \| 0.5784 \| D-E \| \| Column D vs. Column F \| -29.10 \| -156.0 to 97.82 \| No \| ns \| 0.9826 \| D-F \| \| Column E vs. Column F \| 38.15 \| -88.77 to 165.1 \| No \| ns \| 0.9447 \| E-F \| |
| **Figure 8D. BV2 (NLRP3 siRNA transfected), IL-6 mRNA level** |
| \| Number of families \| 1 \|  \|  \|  \|  \|  \| \| --- \| --- \| --- \| --- \| --- \| --- \| --- \| \| Number of comparisons per family \| 15 \|  \|  \|  \|  \|  \| \| Alpha \| 0.05 \|  \|  \|  \|  \|  \| \|  \|  \|  \|  \|  \|  \|  \| \| Tukey's multiple comparisons test \| Mean Diff. \| 95.00% CI of diff. \| Below threshold? \| Summary \| Adjusted P Value \|  \| \| - - vs. + - \| -55.29 \| -69.12 to -41.47 \| Yes \| **** \| <0.0001 \| A-B \| \| - - vs. + + \| -3.680 \| -17.50 to 10.15 \| No \| ns \| 0.9667 \| A-C \| \| - - vs. Column D \| 0.9775 \| -12.85 to 14.80 \| No \| ns \| >0.9999 \| A-D \| \| - - vs. Column E \| 0.4283 \| -13.40 to 14.25 \| No \| ns \| >0.9999 \| A-E \| \| - - vs. Column F \| 0.6097 \| -13.70 to 14.92 \| No \| ns \| >0.9999 \| A-F \| \| + - vs. + + \| 51.61 \| 37.79 to 65.44 \| Yes \| **** \| <0.0001 \| B-C \| \| + - vs. Column D \| 56.27 \| 42.44 to 70.09 \| Yes \| **** \| <0.0001 \| B-D \| \| + - vs. Column E \| 55.72 \| 41.89 to 69.54 \| Yes \| **** \| <0.0001 \| B-E \| \| + - vs. Column F \| 55.90 \| 41.59 to 70.21 \| Yes \| **** \| <0.0001 \| B-F \| \| + + vs. Column D \| 4.657 \| -9.168 to 18.48 \| No \| ns \| 0.9130 \| C-D \| \| + + vs. Column E \| 4.108 \| -9.717 to 17.93 \| No \| ns \| 0.9473 \| C-E \| \| + + vs. Column F \| 4.289 \| -10.02 to 18.60 \| No \| ns \| 0.9453 \| C-F \| \| Column D vs. Column E \| -0.5492 \| -14.37 to 13.28 \| No \| ns \| >0.9999 \| D-E \| \| Column D vs. Column F \| -0.3678 \| -14.68 to 13.94 \| No \| ns \| >0.9999 \| D-F \| \| Column E vs. Column F \| 0.1814 \| -14.13 to 14.49 \| No \| ns \| >0.9999 \| E-F \| |
| **Figure 8E. BV2 (NLRP3 siRNA transfected), TNF-α mRNA level** |
| \| Number of families \| 1 \|  \|  \|  \|  \|  \| \| --- \| --- \| --- \| --- \| --- \| --- \| --- \| \| Number of comparisons per family \| 15 \|  \|  \|  \|  \|  \| \| Alpha \| 0.05 \|  \|  \|  \|  \|  \| \|  \|  \|  \|  \|  \|  \|  \| \| Tukey's multiple comparisons test \| Mean Diff. \| 95.00% CI of diff. \| Below threshold? \| Summary \| Adjusted P Value \|  \| \| - - vs. + - \| -29.32 \| -37.15 to -21.50 \| Yes \| **** \| <0.0001 \| A-B \| \| - - vs. + + \| -4.257 \| -12.09 to 3.571 \| No \| ns \| 0.5870 \| A-C \| \| - - vs. Column D \| -0.3365 \| -8.165 to 7.491 \| No \| ns \| >0.9999 \| A-D \| \| - - vs. Column E \| -3.352 \| -11.18 to 4.476 \| No \| ns \| 0.7942 \| A-E \| \| - - vs. Column F \| -3.248 \| -11.35 to 4.855 \| No \| ns \| 0.8353 \| A-F \| \| + - vs. + + \| 25.07 \| 17.24 to 32.89 \| Yes \| **** \| <0.0001 \| B-C \| \| + - vs. Column D \| 28.99 \| 21.16 to 36.82 \| Yes \| **** \| <0.0001 \| B-D \| \| + - vs. Column E \| 25.97 \| 18.14 to 33.80 \| Yes \| **** \| <0.0001 \| B-E \| \| + - vs. Column F \| 26.08 \| 17.97 to 34.18 \| Yes \| **** \| <0.0001 \| B-F \| \| + + vs. Column D \| 3.921 \| -3.907 to 11.75 \| No \| ns \| 0.6681 \| C-D \| \| + + vs. Column E \| 0.9056 \| -6.922 to 8.734 \| No \| ns \| 0.9993 \| C-E \| \| + + vs. Column F \| 1.010 \| -7.093 to 9.113 \| No \| ns \| 0.9990 \| C-F \| \| Column D vs. Column E \| -3.015 \| -10.84 to 4.813 \| No \| ns \| 0.8568 \| D-E \| \| Column D vs. Column F \| -2.911 \| -11.01 to 5.192 \| No \| ns \| 0.8889 \| D-F \| \| Column E vs. Column F \| 0.1043 \| -7.998 to 8.207 \| No \| ns \| >0.9999 \| E-F \| |
| **Figure 9B, cAMP protein levels in the Cortex** |
| \| Number of families \| 1 \|  \|  \|  \|  \| \| --- \| --- \| --- \| --- \| --- \| --- \| \| Number of comparisons per family \| 3 \|  \|  \|  \|  \| \| Alpha \| 0.05 \|  \|  \|  \|  \| \|  \|  \|  \|  \|  \|  \| \| Newman-Keuls multiple comparisons test \| Mean Diff. \| Below threshold? \| Summary \|  \|  \| \| - - vs. + - \| 20.27 \| Yes \| * \|  \| A-B \| \| - - vs. + + \| 27.40 \| Yes \| * \|  \| A-C \| \| + - vs. + + \| 7.131 \| No \| ns \|  \| B-C \| |
| **Figure 9B, cAMP protein levels in the Hippocampus** |
| \| Number of families \| 1 \|  \|  \|  \|  \| \| --- \| --- \| --- \| --- \| --- \| --- \| \| Number of comparisons per family \| 3 \|  \|  \|  \|  \| \| Alpha \| 0.05 \|  \|  \|  \|  \| \|  \|  \|  \|  \|  \|  \| \| Newman-Keuls multiple comparisons test \| Mean Diff. \| Below threshold? \| Summary \|  \|  \| \| - - vs. + - \| 13.39 \| Yes \| * \|  \| D-E \| \| - - vs. + + \| -2.328 \| No \| ns \|  \| D-F \| \| + - vs. + + \| -15.72 \| Yes \| * \|  \| E-F \| |
| **Figure 9C, D. BV2 (5 μM Selexipag, 90 min), p-p38 protein level** |
| \| Number of families \| 1 \|  \|  \|  \|  \|  \| \| --- \| --- \| --- \| --- \| --- \| --- \| --- \| \| Number of comparisons per family \| 3 \|  \|  \|  \|  \|  \| \| Alpha \| 0.05 \|  \|  \|  \|  \|  \| \|  \|  \|  \|  \|  \|  \|  \| \| Tukey's multiple comparisons test \| Mean diff. \| 95.00% CI of diff. \| Below threshold? \| Summary \| Adjusted P Value \|  \| \| - - vs. + - \| -370.3 \| -472.6 to -267.9 \| Yes \| **** \| <0.0001 \| A-B \| \| - - vs. + + \| -221.2 \| -326.7 to -115.7 \| Yes \| **** \| <0.0001 \| A-C \| \| + - vs. + + \| 149.1 \| 43.53 to 254.6 \| Yes \| ** \| 0.0048 \| B-C \| |
| **Figure 9C, E. BV2 (5 μM Selexipag, 90 min), p38 protein level** |
| \| Number of families \| 1 \|  \|  \|  \|  \|  \| \| --- \| --- \| --- \| --- \| --- \| --- \| --- \| \| Number of comparisons per family \| 3 \|  \|  \|  \|  \|  \| \| Alpha \| 0.05 \|  \|  \|  \|  \|  \| \|  \|  \|  \|  \|  \|  \|  \| \| Tukey's multiple comparisons test \| Mean diff. \| 95.00% CI of diff. \| Below threshold? \| Summary \| Adjusted P Value \|  \| \| - - vs. + - \| 11.26 \| -23.57 to 46.09 \| No \| ns \| 0.7021 \| A-B \| \| - - vs. + + \| 5.862 \| -28.97 to 40.69 \| No \| ns \| 0.9076 \| A-C \| \| + - vs. + + \| -5.399 \| -40.23 to 29.43 \| No \| ns \| 0.9210 \| B-C \| |
| **Figure 9F. BV2 (P38 inhibitor), IL-1β mRNA level** |
| \| Number of families \| 1 \|  \|  \|  \|  \|  \| \| --- \| --- \| --- \| --- \| --- \| --- \| --- \| \| Number of comparisons per family \| 10 \|  \|  \|  \|  \|  \| \| Alpha \| 0.05 \|  \|  \|  \|  \|  \| \|  \|  \|  \|  \|  \|  \|  \| \| Tukey's multiple comparisons test \| Mean diff. \| 95.00% CI of diff. \| Below threshold? \| Summary \| Adjusted P Value \|  \| \| Column A vs. Column B \| -1510 \| -1800 to -1220 \| Yes \| **** \| <0.0001 \| A-B \| \| Column A vs. Column C \| -589.1 \| -879.1 to -299.1 \| Yes \| **** \| <0.0001 \| A-C \| \| Column A vs. Column D \| -246.1 \| -536.1 to 43.83 \| No \| ns \| 0.1284 \| A-D \| \| Column A vs. Column E \| -263.3 \| -553.3 to 26.62 \| No \| ns \| 0.0901 \| A-E \| \| Column B vs. Column C \| 921.1 \| 631.2 to 1211 \| Yes \| **** \| <0.0001 \| B-C \| \| Column B vs. Column D \| 1264 \| 974.1 to 1554 \| Yes \| **** \| <0.0001 \| B-D \| \| Column B vs. Column E \| 1247 \| 956.9 to 1537 \| Yes \| **** \| <0.0001 \| B-E \| \| Column C vs. Column D \| 343.0 \| 52.99 to 632.9 \| Yes \| * \| 0.0137 \| C-D \| \| Column C vs. Column E \| 325.8 \| 35.79 to 615.7 \| Yes \| * \| 0.0212 \| C-E \| \| Column D vs. Column E \| -17.21 \| -307.2 to 272.8 \| No \| ns \| 0.9998 \| D-E \| |
| **Figure 9G. BV2 (P38 inhibitor), IL-6 mRNA level** |
| \| Number of families \| 1 \|  \|  \|  \|  \|  \| \| --- \| --- \| --- \| --- \| --- \| --- \| --- \| \| Number of comparisons per family \| 10 \|  \|  \|  \|  \|  \| \| Alpha \| 0.05 \|  \|  \|  \|  \|  \| \|  \|  \|  \|  \|  \|  \|  \| \| Tukey's multiple comparisons test \| Mean diff. \| 95.00% CI of diff. \| Below threshold? \| Summary \| Adjusted P Value \|  \| \| Column A vs. Column B \| -547.0 \| -645.8 to -448.3 \| Yes \| **** \| <0.0001 \| A-B \| \| Column A vs. Column C \| -96.16 \| -194.9 to 2.594 \| No \| ns \| 0.0594 \| A-C \| \| Column A vs. Column D \| -23.98 \| -122.7 to 74.78 \| No \| ns \| 0.9556 \| A-D \| \| Column A vs. Column E \| -12.05 \| -110.8 to 86.70 \| No \| ns \| 0.9966 \| A-E \| \| Column B vs. Column C \| 450.9 \| 352.1 to 549.6 \| Yes \| **** \| <0.0001 \| B-C \| \| Column B vs. Column D \| 523.1 \| 424.3 to 621.8 \| Yes \| **** \| <0.0001 \| B-D \| \| Column B vs. Column E \| 535.0 \| 436.2 to 633.8 \| Yes \| **** \| <0.0001 \| B-E \| \| Column C vs. Column D \| 72.18 \| -26.57 to 170.9 \| No \| ns \| 0.2422 \| C-D \| \| Column C vs. Column E \| 84.11 \| -14.64 to 182.9 \| No \| ns \| 0.1263 \| C-E \| \| Column D vs. Column E \| 11.93 \| -86.83 to 110.7 \| No \| ns \| 0.9967 \| D-E \| |
| **Figure 9H. BV2 (P38 inhibitor), COX-2 mRNA level** |
| \| Number of families \| 1 \|  \|  \|  \|  \|  \| \| --- \| --- \| --- \| --- \| --- \| --- \| --- \| \| Number of comparisons per family \| 10 \|  \|  \|  \|  \|  \| \| Alpha \| 0.05 \|  \|  \|  \|  \|  \| \|  \|  \|  \|  \|  \|  \|  \| \| Tukey's multiple comparisons test \| Mean diff. \| 95.00% CI of diff. \| Below threshold? \| Summary \| Adjusted P Value \|  \| \| Column A vs. Column B \| -42.53 \| -51.81 to -33.26 \| Yes \| **** \| <0.0001 \| A-B \| \| Column A vs. Column C \| -15.57 \| -24.85 to -6.290 \| Yes \| *** \| 0.0003 \| A-C \| \| Column A vs. Column D \| -14.08 \| -23.36 to -4.803 \| Yes \| *** \| 0.0010 \| A-D \| \| Column A vs. Column E \| -16.95 \| -26.23 to -7.670 \| Yes \| **** \| <0.0001 \| A-E \| \| Column B vs. Column C \| 26.97 \| 17.69 to 36.25 \| Yes \| **** \| <0.0001 \| B-C \| \| Column B vs. Column D \| 28.45 \| 19.17 to 37.73 \| Yes \| **** \| <0.0001 \| B-D \| \| Column B vs. Column E \| 25.59 \| 16.31 to 34.86 \| Yes \| **** \| <0.0001 \| B-E \| \| Column C vs. Column D \| 1.486 \| -7.793 to 10.77 \| No \| ns \| 0.9903 \| C-D \| \| Column C vs. Column E \| -1.381 \| -10.66 to 7.898 \| No \| ns \| 0.9927 \| C-E \| \| Column D vs. Column E \| -2.867 \| -12.15 to 6.412 \| No \| ns \| 0.8993 \| D-E \| |
| **Figure 9I. BV2 (P38 inhibitor), TNF-α mRNA level** |
| \| Number of families \| 1 \|  \|  \|  \|  \|  \| \| --- \| --- \| --- \| --- \| --- \| --- \| --- \| \| Number of comparisons per family \| 10 \|  \|  \|  \|  \|  \| \| Alpha \| 0.05 \|  \|  \|  \|  \|  \| \|  \|  \|  \|  \|  \|  \|  \| \| Tukey's multiple comparisons test \| Mean diff. \| 95.00% CI of diff. \| Below threshold? \| Summary \| Adjusted P Value \|  \| \| Column A vs. Column B \| -35.71 \| -40.87 to -30.55 \| Yes \| **** \| <0.0001 \| A-B \| \| Column A vs. Column C \| -6.076 \| -11.24 to -0.9168 \| Yes \| * \| 0.0143 \| A-C \| \| Column A vs. Column D \| -13.83 \| -18.99 to -8.667 \| Yes \| **** \| <0.0001 \| A-D \| \| Column A vs. Column E \| -9.647 \| -14.81 to -4.487 \| Yes \| **** \| <0.0001 \| A-E \| \| Column B vs. Column C \| 29.63 \| 24.47 to 34.79 \| Yes \| **** \| <0.0001 \| B-C \| \| Column B vs. Column D \| 21.88 \| 16.72 to 27.04 \| Yes \| **** \| <0.0001 \| B-D \| \| Column B vs. Column E \| 26.06 \| 20.90 to 31.22 \| Yes \| **** \| <0.0001 \| B-E \| \| Column C vs. Column D \| -7.750 \| -12.91 to -2.590 \| Yes \| ** \| 0.0011 \| C-D \| \| Column C vs. Column E \| -3.570 \| -8.730 to 1.589 \| No \| ns \| 0.2921 \| C-E \| \| Column D vs. Column E \| 4.180 \| -0.9798 to 9.340 \| No \| ns \| 0.1599 \| D-E \| |
| **Supplementary Figure 1A, B. BV2 (5 μM Selexipag), p-ERK protein level** |
| \| Number of families \| 1 \|  \|  \|  \|  \|  \| \| --- \| --- \| --- \| --- \| --- \| --- \| --- \| \| Number of comparisons per family \| 3 \|  \|  \|  \|  \|  \| \| Alpha \| 0.05 \|  \|  \|  \|  \|  \| \|  \|  \|  \|  \|  \|  \|  \| \| Tukey's multiple comparisons test \| Mean Diff. \| 95.00% CI of diff. \| Below threshold? \| Summary \| Adjusted P Value \|  \| \| - - vs. + - \| -45.50 \| -76.66 to -14.34 \| Yes \| ** \| 0.0045 \| A-B \| \| - - vs. + + \| -34.52 \| -65.68 to -3.357 \| Yes \| * \| 0.0290 \| A-C \| \| + - vs. + + \| 10.98 \| -20.18 to 42.14 \| No \| ns \| 0.6425 \| B-C \| |
| **Supplementary Figure 1A, C. BV2 (5 μM Selexipag), ERK protein level** |
| \| Number of families \| 1 \|  \|  \|  \|  \|  \| \| --- \| --- \| --- \| --- \| --- \| --- \| --- \| \| Number of comparisons per family \| 3 \|  \|  \|  \|  \|  \| \| Alpha \| 0.05 \|  \|  \|  \|  \|  \| \|  \|  \|  \|  \|  \|  \|  \| \| Tukey's multiple comparisons test \| Mean Diff. \| 95.00% CI of diff. \| Below threshold? \| Summary \| Adjusted P Value \|  \| \| - - vs. + - \| 0.9970 \| -32.59 to 34.58 \| No \| ns \| 0.9970 \| A-B \| \| - - vs. + + \| -7.155 \| -40.74 to 26.43 \| No \| ns \| 0.8564 \| A-C \| \| + - vs. + + \| -8.152 \| -41.74 to 25.43 \| No \| ns \| 0.8181 \| B-C \| |
| **Supplementary Figure 2A. BV2 (IP receptor antagonist), COX-2 mRNA level** |
| \| Number of families \| 1 \|  \|  \|  \|  \|  \| \| --- \| --- \| --- \| --- \| --- \| --- \| --- \| \| Number of comparisons per family \| 6 \|  \|  \|  \|  \|  \| \| Alpha \| 0.05 \|  \|  \|  \|  \|  \| \|  \|  \|  \|  \|  \|  \|  \| \| Tukey's multiple comparisons test \| Mean diff. \| 95.00% CI of diff. \| Below threshold? \| Summary \| Adjusted P Value \|  \| \| Column A vs. Column B \| -94.45 \| -111.6 to -77.32 \| Yes \| **** \| <0.0001 \| A-B \| \| Column A vs. Column C \| -21.26 \| -38.39 to -4.136 \| Yes \| * \| 0.0118 \| A-C \| \| Column A vs. Column D \| -19.48 \| -36.60 to -2.350 \| Yes \| * \| 0.0223 \| A-D \| \| Column B vs. Column C \| 73.19 \| 56.06 to 90.31 \| Yes \| **** \| <0.0001 \| B-C \| \| Column B vs. Column D \| 74.97 \| 57.85 to 92.10 \| Yes \| **** \| <0.0001 \| B-D \| \| Column C vs. Column D \| 1.786 \| -15.34 to 18.91 \| No \| ns \| 0.9911 \| C-D \| |
| **Supplementary Figure 2B. BV2 (IP receptor antagonist), IL-1β mRNA level** |
| \| Number of families \| 1 \|  \|  \|  \|  \|  \| \| --- \| --- \| --- \| --- \| --- \| --- \| --- \| \| Number of comparisons per family \| 6 \|  \|  \|  \|  \|  \| \| Alpha \| 0.05 \|  \|  \|  \|  \|  \| \|  \|  \|  \|  \|  \|  \|  \| \| Tukey's multiple comparisons test \| Mean diff. \| 95.00% CI of diff. \| Below threshold? \| Summary \| Adjusted P Value \|  \| \| Column A vs. Column B \| -6368 \| -8828 to -3907 \| Yes \| **** \| <0.0001 \| A-B \| \| Column A vs. Column C \| -5111 \| -7571 to -2650 \| Yes \| **** \| <0.0001 \| A-C \| \| Column A vs. Column D \| -4372 \| -6833 to -1912 \| Yes \| *** \| 0.0004 \| A-D \| \| Column B vs. Column C \| 1257 \| -1203 to 3717 \| No \| ns \| 0.4963 \| B-C \| \| Column B vs. Column D \| 1996 \| -464.6 to 4456 \| No \| ns \| 0.1388 \| B-D \| \| Column C vs. Column D \| 738.9 \| -1722 to 3199 \| No \| ns \| 0.8346 \| C-D \| |
| **Supplementary Figure 2C. BV2 (1 μM IP receptor antagonist), cAMP protein level** |
| \| Number of families \| 1 \|  \|  \|  \|  \|  \| \| --- \| --- \| --- \| --- \| --- \| --- \| --- \| \| Number of comparisons per family \| 10 \|  \|  \|  \|  \|  \| \| Alpha \| 0.05 \|  \|  \|  \|  \|  \| \|  \|  \|  \|  \|  \|  \|  \| \| Tukey's multiple comparisons test \| Mean diff. \| 95.00% CI of diff. \| Below threshold? \| Summary \| Adjusted P Value \|  \| \| Column A vs. Column B \| 2.005 \| -2.300 to 6.309 \| No \| ns \| 0.6692 \| A-B \| \| Column A vs. Column C \| -3.393 \| -7.698 to 0.9113 \| No \| ns \| 0.1801 \| A-C \| \| Column A vs. Column D \| 10.40 \| 6.091 to 14.70 \| Yes \| **** \| <0.0001 \| A-D \| \| Column A vs. Column E \| 11.41 \| 7.101 to 15.71 \| Yes \| **** \| <0.0001 \| A-E \| \| Column B vs. Column C \| -5.398 \| -9.702 to -1.094 \| Yes \| ** \| 0.0080 \| B-C \| \| Column B vs. Column D \| 8.390 \| 4.086 to 12.69 \| Yes \| **** \| <0.0001 \| B-D \| \| Column B vs. Column E \| 9.401 \| 5.096 to 13.71 \| Yes \| **** \| <0.0001 \| B-E \| \| Column C vs. Column D \| 13.79 \| 9.484 to 18.09 \| Yes \| **** \| <0.0001 \| C-D \| \| Column C vs. Column E \| 14.80 \| 10.49 to 19.10 \| Yes \| **** \| <0.0001 \| C-E \| \| Column D vs. Column E \| 1.010 \| -3.294 to 5.315 \| No \| ns \| 0.9606 \| D-E \| |
| **Supplementary Figure 2C. BV2 (0.01 nM IP receptor antagonist), IL-1β mRNA level** |
| \| Number of families \| 1 \|  \|  \|  \|  \|  \| \| --- \| --- \| --- \| --- \| --- \| --- \| --- \| \| Number of comparisons per family \| 10 \|  \|  \|  \|  \|  \| \| Alpha \| 0.05 \|  \|  \|  \|  \|  \| \|  \|  \|  \|  \|  \|  \|  \| \| Tukey's multiple comparisons test \| Mean diff. \| 95.00% CI of diff. \| Below threshold? \| Summary \| Adjusted P Value \|  \| \| Column A vs. Column B \| -3156 \| -4124 to -2187 \| Yes \| **** \| <0.0001 \| A-B \| \| Column A vs. Column C \| -1446 \| -2415 to -478.2 \| Yes \| ** \| 0.0012 \| A-C \| \| Column A vs. Column D \| -2313 \| -3281 to -1344 \| Yes \| **** \| <0.0001 \| A-D \| \| Column A vs. Column E \| -1891 \| -2860 to -923.0 \| Yes \| **** \| <0.0001 \| A-E \| \| Column B vs. Column C \| 1709 \| 741.0 to 2678 \| Yes \| *** \| 0.0001 \| B-C \| \| Column B vs. Column D \| 843.0 \| -125.3 to 1811 \| No \| ns \| 0.1130 \| B-D \| \| Column B vs. Column E \| 1264 \| 296.2 to 2233 \| Yes \| ** \| 0.0054 \| B-E \| \| Column C vs. Column D \| -866.3 \| -1835 to 102.0 \| No \| ns \| 0.0978 \| C-D \| \| Column C vs. Column E \| -444.9 \| -1413 to 523.4 \| No \| ns \| 0.6803 \| C-E \| \| Column D vs. Column E \| 421.4 \| -546.9 to 1390 \| No \| ns \| 0.7218 \| D-E \| |
| **Supplementary Figure 3A, B. p-c-Jun^S73^ protein levels in the Hippocampus of C57BL/6N mice** |
| \| Number of families \| 1 \|  \|  \|  \|  \| \| --- \| --- \| --- \| --- \| --- \| --- \| \| Number of comparisons per family \| 3 \|  \|  \|  \|  \| \| Alpha \| 0.05 \|  \|  \|  \|  \| \|  \|  \|  \|  \|  \|  \| \| Newman-Keuls multiple comparisons test \| Mean Diff. \| Below threshold? \| Summary \|  \|  \| \| - - vs. + - \| -107.2 \| Yes \| *** \|  \| A-B \| \| - - vs. + + \| -54.09 \| Yes \| * \|  \| A-C \| \| + - vs. + + \| 53.14 \| Yes \| * \|  \| B-C \| |
| **Supplementary Figure 3A, C. c-Jun protein levels in the Hippocampus of C57BL/6N mice** |
| \| Number of families \| 1 \|  \|  \|  \|  \|  \| \| --- \| --- \| --- \| --- \| --- \| --- \| --- \| \| Number of comparisons per family \| 3 \|  \|  \|  \|  \|  \| \| Alpha \| 0.05 \|  \|  \|  \|  \|  \| \|  \|  \|  \|  \|  \|  \|  \| \| Tukey's multiple comparisons test \| Mean Diff. \| 95.00% CI of diff. \| Below threshold? \| Summary \| Adjusted P Value \|  \| \| - - vs. + - \| -57.73 \| -100.6 to -14.83 \| Yes \| ** \| 0.0074 \| A-B \| \| - - vs. + + \| -40.87 \| -83.78 to 2.033 \| No \| ns \| 0.0637 \| A-C \| \| + - vs. + + \| 16.86 \| -26.04 to 59.77 \| No \| ns \| 0.5907 \| B-C \| |
| **Supplementary Figure 3D, E. p-c-Jun^S73^ protein levels in the Cortex of C57BL/6N mice** |
| \| Number of families \| 1 \|  \|  \|  \|  \| \| --- \| --- \| --- \| --- \| --- \| --- \| \| Number of comparisons per family \| 3 \|  \|  \|  \|  \| \| Alpha \| 0.05 \|  \|  \|  \|  \| \|  \|  \|  \|  \|  \|  \| \| Newman-Keuls multiple comparisons test \| Mean Diff. \| Below threshold? \| Summary \|  \|  \| \| - - vs. + - \| -154.8 \| Yes \| ** \|  \| A-B \| \| - - vs. + + \| -103.0 \| Yes \| * \|  \| A-C \| \| + - vs. + + \| 51.83 \| No \| ns \|  \| B-C \| |
| **Supplementary Figure 3D, F. c-Jun protein levels in the Cortex of C57BL/6N mice** |
| \| Number of families \| 1 \|  \|  \|  \|  \|  \| \| --- \| --- \| --- \| --- \| --- \| --- \| --- \| \| Number of comparisons per family \| 3 \|  \|  \|  \|  \|  \| \| Alpha \| 0.05 \|  \|  \|  \|  \|  \| \|  \|  \|  \|  \|  \|  \|  \| \| Tukey's multiple comparisons test \| Mean Diff. \| 95.00% CI of diff. \| Below threshold? \| Summary \| Adjusted P Value \|  \| \| - - vs. + - \| -137.5 \| -194.2 to -80.89 \| Yes \| **** \| <0.0001 \| A-B \| \| - - vs. + + \| -124.2 \| -180.8 to -67.50 \| Yes \| **** \| <0.0001 \| A-C \| \| + - vs. + + \| 13.39 \| -43.27 to 70.05 \| No \| ns \| 0.8239 \| B-C \| |
| **Supplementary Figure 4A, B. p-STAT^Y705^ protein levels in the Hippocampus of C57BL/6N mice** |
| \| Number of families \| 1 \|  \|  \|  \|  \|  \| \| --- \| --- \| --- \| --- \| --- \| --- \| --- \| \| Number of comparisons per family \| 3 \|  \|  \|  \|  \|  \| \| Alpha \| 0.05 \|  \|  \|  \|  \|  \| \|  \|  \|  \|  \|  \|  \|  \| \| Tukey's multiple comparisons test \| Mean Diff. \| 95.00% CI of diff. \| Below threshold? \| Summary \| Adjusted P Value \|  \| \| Vehicle vs. LPS \| -12330 \| -16194 to -8467 \| Yes \| **** \| <0.0001 \| A-B \| \| Vehicle vs. Selexipag \| -12470 \| -16333 to -8606 \| Yes \| **** \| <0.0001 \| A-C \| \| LPS vs. Selexipag \| -139.5 \| -4003 to 3724 \| No \| ns \| 0.9954 \| B-C \| |
| **Supplementary Figure 4A, C. STAT3 protein levels in the Hippocampus of C57BL/6N mice** |
| \| Number of families \| 1 \|  \|  \|  \|  \|  \| \| --- \| --- \| --- \| --- \| --- \| --- \| --- \| \| Number of comparisons per family \| 3 \|  \|  \|  \|  \|  \| \| Alpha \| 0.05 \|  \|  \|  \|  \|  \| \|  \|  \|  \|  \|  \|  \|  \| \| Tukey's multiple comparisons test \| Mean Diff. \| 95.00% CI of diff. \| Below threshold? \| Summary \| Adjusted P Value \|  \| \| Vehicle vs. LPS \| -2.976 \| -25.82 to 19.87 \| No \| ns \| 0.9425 \| A-B \| \| Vehicle vs. Selexipag \| -0.6033 \| -23.45 to 22.24 \| No \| ns \| 0.9976 \| A-C \| \| LPS vs. Selexipag \| 2.372 \| -20.48 to 25.22 \| No \| ns \| 0.9630 \| B-C \| |
| **Supplementary Figure 4D, E. p-STAT^Y705^ protein levels in the Cortex of C57BL/6N mice** |
| \| Number of families \| 1 \|  \|  \|  \|  \|  \| \| --- \| --- \| --- \| --- \| --- \| --- \| --- \| \| Number of comparisons per family \| 3 \|  \|  \|  \|  \|  \| \| Alpha \| 0.05 \|  \|  \|  \|  \|  \| \|  \|  \|  \|  \|  \|  \|  \| \| Tukey's multiple comparisons test \| Mean Diff. \| 95.00% CI of diff. \| Below threshold? \| Summary \| Adjusted P Value \|  \| \| Vehicle vs. LPS \| -18273 \| -26995 to -9552 \| Yes \| **** \| <0.0001 \| A-B \| \| Vehicle vs. LPS+BMS \| -19356 \| -28077 to -10634 \| Yes \| **** \| <0.0001 \| A-C \| \| LPS vs. LPS+BMS \| -1082 \| -9804 to 7639 \| No \| ns \| 0.9476 \| B-C \| |
| **Supplementary Figure 4D, F. STAT3 protein levels in the Cortex of C57BL/6N mice** |
| \| Number of families \| 1 \|  \|  \|  \|  \|  \| \| --- \| --- \| --- \| --- \| --- \| --- \| --- \| \| Number of comparisons per family \| 3 \|  \|  \|  \|  \|  \| \| Alpha \| 0.05 \|  \|  \|  \|  \|  \| \|  \|  \|  \|  \|  \|  \|  \| \| Tukey's multiple comparisons test \| Mean Diff. \| 95.00% CI of diff. \| Below threshold? \| Summary \| Adjusted P Value \|  \| \| Vehicle vs. LPS \| 14.41 \| -27.11 to 55.93 \| No \| ns \| 0.6617 \| A-B \| \| Vehicle vs. LPS+BMS \| 11.64 \| -29.88 to 53.17 \| No \| ns \| 0.7623 \| A-C \| \| LPS vs. LPS+BMS \| -2.769 \| -44.29 to 38.76 \| No \| ns \| 0.9846 \| B-C \| |
| **Supplementary Figure 5A-B. BV2 (c-Jun inhibitor), p-c-Jun protein level** |
| \| Number of families \| 1 \|  \|  \|  \|  \|  \| \| --- \| --- \| --- \| --- \| --- \| --- \| --- \| \| Number of comparisons per family \| 10 \|  \|  \|  \|  \|  \| \| Alpha \| 0.05 \|  \|  \|  \|  \|  \| \|  \|  \|  \|  \|  \|  \|  \| \| Tukey's multiple comparisons test \| Mean diff. \| 95.00% CI of diff. \| Below threshold? \| Summary \| Adjusted P Value \|  \| \| Column A vs. Column B \| -316.5 \| -553.1 to -79.85 \| Yes \| ** \| 0.0042 \| A-B \| \| Column A vs. Column C \| -101.7 \| -338.4 to 134.9 \| No \| ns \| 0.7308 \| A-C \| \| Column A vs. Column D \| -141.1 \| -377.7 to 95.55 \| No \| ns \| 0.4387 \| A-D \| \| Column A vs. Column E \| -59.77 \| -296.4 to 176.9 \| No \| ns \| 0.9490 \| A-E \| \| Column B vs. Column C \| 214.8 \| -21.86 to 451.4 \| No \| ns \| 0.0904 \| B-C \| \| Column B vs. Column D \| 175.4 \| -61.23 to 412.0 \| No \| ns \| 0.2301 \| B-D \| \| Column B vs. Column E \| 256.7 \| 20.08 to 493.4 \| Yes \| * \| 0.0279 \| B-E \| \| Column C vs. Column D \| -39.37 \| -276.0 to 197.3 \| No \| ns \| 0.9889 \| C-D \| \| Column C vs. Column E \| 41.95 \| -194.7 to 278.6 \| No \| ns \| 0.9859 \| C-E \| \| Column D vs. Column E \| 81.32 \| -155.3 to 318.0 \| No \| ns \| 0.8589 \| D-E \| |
| **Supplementary Figure 5C. BV2 (c-Jun inhibitor), IL-1β mRNA level** |
| \| Number of families \| 1 \|  \|  \|  \|  \|  \| \| --- \| --- \| --- \| --- \| --- \| --- \| --- \| \| Number of comparisons per family \| 10 \|  \|  \|  \|  \|  \| \| Alpha \| 0.05 \|  \|  \|  \|  \|  \| \|  \|  \|  \|  \|  \|  \|  \| \| Tukey's multiple comparisons test \| Mean diff. \| 95.00% CI of diff. \| Below threshold? \| Summary \| Adjusted P Value \|  \| \| Column A vs. Column B \| -899.4 \| -1207 to -591.7 \| Yes \| **** \| <0.0001 \| A-B \| \| Column A vs. Column C \| -532.2 \| -839.9 to -224.4 \| Yes \| *** \| 0.0002 \| A-C \| \| Column A vs. Column D \| -1449 \| -1757 to -1142 \| Yes \| **** \| <0.0001 \| A-D \| \| Column A vs. Column E \| -923.4 \| -1231 to -615.6 \| Yes \| **** \| <0.0001 \| A-E \| \| Column B vs. Column C \| 367.2 \| 59.49 to 674.9 \| Yes \| * \| 0.0127 \| B-C \| \| Column B vs. Column D \| -550.0 \| -857.7 to -242.3 \| Yes \| **** \| <0.0001 \| B-D \| \| Column B vs. Column E \| -23.99 \| -331.7 to 283.7 \| No \| ns \| 0.9994 \| B-E \| \| Column C vs. Column D \| -917.2 \| -1225 to -609.5 \| Yes \| **** \| <0.0001 \| C-D \| \| Column C vs. Column E \| -391.2 \| -698.9 to -83.48 \| Yes \| ** \| 0.0070 \| C-E \| \| Column D vs. Column E \| 526.0 \| 218.3 to 833.7 \| Yes \| *** \| 0.0002 \| D-E \| |
| **Supplementary Figure 5D. BV2 (c-Jun inhibitor), IL-6 mRNA level** |
| \| Number of families \| 1 \|  \|  \|  \|  \|  \| \| --- \| --- \| --- \| --- \| --- \| --- \| --- \| \| Number of comparisons per family \| 10 \|  \|  \|  \|  \|  \| \| Alpha \| 0.05 \|  \|  \|  \|  \|  \| \|  \|  \|  \|  \|  \|  \|  \| \| Tukey's multiple comparisons test \| Mean diff. \| 95.00% CI of diff. \| Below threshold? \| Summary \| Adjusted P Value \|  \| \| Column A vs. Column B \| -303.4 \| -330.3 to -276.6 \| Yes \| **** \| <0.0001 \| A-B \| \| Column A vs. Column C \| -42.86 \| -69.71 to -16.02 \| Yes \| *** \| 0.0005 \| A-C \| \| Column A vs. Column D \| -306.7 \| -333.5 to -279.8 \| Yes \| **** \| <0.0001 \| A-D \| \| Column A vs. Column E \| -68.48 \| -95.32 to -41.63 \| Yes \| **** \| <0.0001 \| A-E \| \| Column B vs. Column C \| 260.6 \| 233.7 to 287.4 \| Yes \| **** \| <0.0001 \| B-C \| \| Column B vs. Column D \| -3.271 \| -30.12 to 23.57 \| No \| ns \| 0.9966 \| B-D \| \| Column B vs. Column E \| 234.9 \| 208.1 to 261.8 \| Yes \| **** \| <0.0001 \| B-E \| \| Column C vs. Column D \| -263.8 \| -290.7 to -237.0 \| Yes \| **** \| <0.0001 \| C-D \| \| Column C vs. Column E \| -25.61 \| -52.46 to 1.232 \| No \| ns \| 0.0675 \| C-E \| \| Column D vs. Column E \| 238.2 \| 211.4 to 265.1 \| Yes \| **** \| <0.0001 \| D-E \| |
| **Supplementary Figure 5E. BV2 (c-Jun inhibitor), COX-2 mRNA level** |
| \| Number of families \| 1 \|  \|  \|  \|  \|  \| \| --- \| --- \| --- \| --- \| --- \| --- \| --- \| \| Number of comparisons per family \| 10 \|  \|  \|  \|  \|  \| \| Alpha \| 0.05 \|  \|  \|  \|  \|  \| \|  \|  \|  \|  \|  \|  \|  \| \| Tukey's multiple comparisons test \| Mean diff. \| 95.00% CI of diff. \| Below threshold? \| Summary \| Adjusted P Value \|  \| \| Column A vs. Column B \| -56.90 \| -76.00 to -37.81 \| Yes \| **** \| <0.0001 \| A-B \| \| Column A vs. Column C \| -24.36 \| -43.46 to -5.258 \| Yes \| ** \| 0.0068 \| A-C \| \| Column A vs. Column D \| -83.28 \| -102.4 to -64.18 \| Yes \| **** \| <0.0001 \| A-D \| \| Column A vs. Column E \| -44.07 \| -63.17 to -24.97 \| Yes \| **** \| <0.0001 \| A-E \| \| Column B vs. Column C \| 32.55 \| 13.45 to 51.65 \| Yes \| *** \| 0.0002 \| B-C \| \| Column B vs. Column D \| -26.37 \| -45.47 to -7.275 \| Yes \| ** \| 0.0030 \| B-D \| \| Column B vs. Column E \| 12.83 \| -6.266 to 31.93 \| No \| ns \| 0.3201 \| B-E \| \| Column C vs. Column D \| -58.92 \| -78.02 to -39.82 \| Yes \| **** \| <0.0001 \| C-D \| \| Column C vs. Column E \| -19.72 \| -38.81 to -0.6163 \| Yes \| * \| 0.0402 \| C-E \| \| Column D vs. Column E \| 39.21 \| 20.11 to 58.31 \| Yes \| **** \| <0.0001 \| D-E \| |
| **Supplementary Figure 5F. BV2 (c-Jun inhibitor), TNF-α mRNA level** |
| \| Number of families \| 1 \|  \|  \|  \|  \|  \| \| --- \| --- \| --- \| --- \| --- \| --- \| --- \| \| Number of comparisons per family \| 10 \|  \|  \|  \|  \|  \| \| Alpha \| 0.05 \|  \|  \|  \|  \|  \| \|  \|  \|  \|  \|  \|  \|  \| \| Tukey's multiple comparisons test \| Mean diff. \| 95.00% CI of diff. \| Below threshold? \| Summary \| Adjusted P Value \|  \| \| Column A vs. Column B \| -55.91 \| -62.37 to -49.46 \| Yes \| **** \| <0.0001 \| A-B \| \| Column A vs. Column C \| -12.72 \| -19.17 to -6.259 \| Yes \| **** \| <0.0001 \| A-C \| \| Column A vs. Column D \| -59.32 \| -65.78 to -52.86 \| Yes \| **** \| <0.0001 \| A-D \| \| Column A vs. Column E \| -17.61 \| -24.07 to -11.16 \| Yes \| **** \| <0.0001 \| A-E \| \| Column B vs. Column C \| 43.20 \| 36.74 to 49.65 \| Yes \| **** \| <0.0001 \| B-C \| \| Column B vs. Column D \| -3.405 \| -9.863 to 3.053 \| No \| ns \| 0.5595 \| B-D \| \| Column B vs. Column E \| 38.30 \| 31.84 to 44.76 \| Yes \| **** \| <0.0001 \| B-E \| \| Column C vs. Column D \| -46.60 \| -53.06 to -40.14 \| Yes \| **** \| <0.0001 \| C-D \| \| Column C vs. Column E \| -4.897 \| -11.35 to 1.561 \| No \| ns \| 0.2110 \| C-E \| \| Column D vs. Column E \| 41.70 \| 35.25 to 48.16 \| Yes \| **** \| <0.0001 \| D-E \| |
| **Supplementary Figure 6A. GSDMD mRNA level in the Cortex of C57BL/6N mice** |
| \| Number of families \| 1 \|  \|  \|  \|  \|  \| \| --- \| --- \| --- \| --- \| --- \| --- \| --- \| \| Number of comparisons per family \| 3 \|  \|  \|  \|  \|  \| \| Alpha \| 0.05 \|  \|  \|  \|  \|  \| \|  \|  \|  \|  \|  \|  \|  \| \| Tukey's multiple comparisons test \| Mean Diff. \| 95.00% CI of diff. \| Below threshold? \| Summary \| Adjusted P Value \|  \| \| - - vs. + - \| -3.022 \| -3.853 to -2.191 \| Yes \| **** \| <0.0001 \| A-B \| \| - - vs. + + \| -1.490 \| -2.321 to -0.6588 \| Yes \| *** \| 0.0005 \| A-C \| \| + - vs. + + \| 1.532 \| 0.7015 to 2.363 \| Yes \| *** \| 0.0004 \| B-C \| |
| **Supplementary Figure 6A. GSDMD mRNA level in the Hippocampus of C57BL/6N mice** |
| \| Number of families \| 1 \|  \|  \|  \|  \|  \| \| --- \| --- \| --- \| --- \| --- \| --- \| --- \| \| Number of comparisons per family \| 3 \|  \|  \|  \|  \|  \| \| Alpha \| 0.05 \|  \|  \|  \|  \|  \| \|  \|  \|  \|  \|  \|  \|  \| \| Tukey's multiple comparisons test \| Mean Diff. \| 95.00% CI of diff. \| Below threshold? \| Summary \| Adjusted P Value \|  \| \| - - vs. + - \| -1.804 \| -2.576 to -1.032 \| Yes \| **** \| <0.0001 \| D-E \| \| - - vs. + + \| -1.021 \| -1.793 to -0.2485 \| Yes \| ** \| 0.0085 \| D-F \| \| + - vs. + + \| 0.7835 \| 0.01123 to 1.556 \| Yes \| * \| 0.0464 \| E-F \| |
| **Supplementary Figure 6B. NLRP6 mRNA level in the Cortex of C57BL/6N mice** |
| \| Number of families \| 1 \|  \|  \|  \|  \|  \| \| --- \| --- \| --- \| --- \| --- \| --- \| --- \| \| Number of comparisons per family \| 3 \|  \|  \|  \|  \|  \| \| Alpha \| 0.05 \|  \|  \|  \|  \|  \| \|  \|  \|  \|  \|  \|  \|  \| \| Tukey's multiple comparisons test \| Mean Diff. \| 95.00% CI of diff. \| Below threshold? \| Summary \| Adjusted P Value \|  \| \| - - vs. + - \| -4.538 \| -6.209 to -2.866 \| Yes \| **** \| <0.0001 \| A-B \| \| - - vs. + + \| -4.086 \| -5.757 to -2.414 \| Yes \| **** \| <0.0001 \| A-C \| \| + - vs. + + \| 0.4520 \| -1.219 to 2.124 \| No \| ns \| 0.7766 \| B-C \| |
| **Supplementary Figure 6B. NLRP6 mRNA level in the Hippocampus of C57BL/6N mice** |
| \| Number of families \| 1 \|  \|  \|  \|  \|  \| \| --- \| --- \| --- \| --- \| --- \| --- \| --- \| \| Number of comparisons per family \| 3 \|  \|  \|  \|  \|  \| \| Alpha \| 0.05 \|  \|  \|  \|  \|  \| \|  \|  \|  \|  \|  \|  \|  \| \| Tukey's multiple comparisons test \| Mean Diff. \| 95.00% CI of diff. \| Below threshold? \| Summary \| Adjusted P Value \|  \| \| - - vs. + - \| -0.9376 \| -1.559 to -0.3164 \| Yes \| ** \| 0.0029 \| D-E \| \| - - vs. + + \| -0.7188 \| -1.340 to -0.09759 \| Yes \| * \| 0.0216 \| D-F \| \| + - vs. + + \| 0.2188 \| -0.4025 to 0.8400 \| No \| ns \| 0.6538 \| E-F \| |
| **Supplementary Figure 6C. CASPASE-1 mRNA level in the Cortex of C57BL/6N mice** |
| \| Number of families \| 1 \|  \|  \|  \|  \|  \| \| --- \| --- \| --- \| --- \| --- \| --- \| --- \| \| Number of comparisons per family \| 3 \|  \|  \|  \|  \|  \| \| Alpha \| 0.05 \|  \|  \|  \|  \|  \| \|  \|  \|  \|  \|  \|  \|  \| \| Tukey's multiple comparisons test \| Mean Diff. \| 95.00% CI of diff. \| Below threshold? \| Summary \| Adjusted P Value \|  \| \| - - vs. + - \| -0.6503 \| -1.060 to -0.2410 \| Yes \| ** \| 0.0018 \| A-B \| \| - - vs. + + \| -0.4200 \| -0.8293 to -0.01066 \| Yes \| * \| 0.0437 \| A-C \| \| + - vs. + + \| 0.2303 \| -0.1790 to 0.6396 \| No \| ns \| 0.3498 \| B-C \| |
| **Supplementary Figure 6C. CASPASE-1 mRNA level in the Hippocampus of C57BL/6N mice** |
| \| Number of families \| 1 \|  \|  \|  \|  \|  \| \| --- \| --- \| --- \| --- \| --- \| --- \| --- \| \| Number of comparisons per family \| 3 \|  \|  \|  \|  \|  \| \| Alpha \| 0.05 \|  \|  \|  \|  \|  \| \|  \|  \|  \|  \|  \|  \|  \| \| Tukey's multiple comparisons test \| Mean Diff. \| 95.00% CI of diff. \| Below threshold? \| Summary \| Adjusted P Value \|  \| \| - - vs. + - \| -0.02077 \| -0.3601 to 0.3185 \| No \| ns \| 0.9870 \| D-E \| \| - - vs. + + \| -0.07615 \| -0.4155 to 0.2632 \| No \| ns \| 0.8396 \| D-F \| \| + - vs. + + \| -0.05538 \| -0.3947 to 0.2839 \| No \| ns \| 0.9113 \| E-F \| |
| **Supplementary Figure 6D. ASC mRNA level in the Cortex of C57BL/6N mice** |
| \| Number of families \| 1 \|  \|  \|  \|  \|  \| \| --- \| --- \| --- \| --- \| --- \| --- \| --- \| \| Number of comparisons per family \| 3 \|  \|  \|  \|  \|  \| \| Alpha \| 0.05 \|  \|  \|  \|  \|  \| \|  \|  \|  \|  \|  \|  \|  \| \| Tukey's multiple comparisons test \| Mean Diff. \| 95.00% CI of diff. \| Below threshold? \| Summary \| Adjusted P Value \|  \| \| - - vs. + - \| -1.140 \| -1.673 to -0.6079 \| Yes \| **** \| <0.0001 \| A-B \| \| - - vs. + + \| -0.9072 \| -1.440 to -0.3747 \| Yes \| *** \| 0.0009 \| A-C \| \| + - vs. + + \| 0.2333 \| -0.2993 to 0.7658 \| No \| ns \| 0.5222 \| B-C \| |
| **Supplementary Figure 6D. ASC mRNA level in the Hippocampus of C57BL/6N mice** |
| \| Number of families \| 1 \|  \|  \|  \|  \|  \| \| --- \| --- \| --- \| --- \| --- \| --- \| --- \| \| Number of comparisons per family \| 3 \|  \|  \|  \|  \|  \| \| Alpha \| 0.05 \|  \|  \|  \|  \|  \| \|  \|  \|  \|  \|  \|  \|  \| \| Tukey's multiple comparisons test \| Mean Diff. \| 95.00% CI of diff. \| Below threshold? \| Summary \| Adjusted P Value \|  \| \| - - vs. + - \| -1.600 \| -2.403 to -0.7975 \| Yes \| *** \| 0.0002 \| D-E \| \| - - vs. + + \| -1.045 \| -1.848 to -0.2422 \| Yes \| ** \| 0.0096 \| D-F \| \| + - vs. + + \| 0.5553 \| -0.2476 to 1.358 \| No \| ns \| 0.2131 \| E-F \| |
| **Supplementary Figure 6E. IL-18 mRNA level in the Cortex of C57BL/6N mice** |
| \| Number of families \| 1 \|  \|  \|  \|  \|  \| \| --- \| --- \| --- \| --- \| --- \| --- \| --- \| \| Number of comparisons per family \| 3 \|  \|  \|  \|  \|  \| \| Alpha \| 0.05 \|  \|  \|  \|  \|  \| \|  \|  \|  \|  \|  \|  \|  \| \| Tukey's multiple comparisons test \| Mean Diff. \| 95.00% CI of diff. \| Below threshold? \| Summary \| Adjusted P Value \|  \| \| - - vs. + - \| -0.02148 \| -0.5112 to 0.4682 \| No \| ns \| 0.9933 \| A-B \| \| - - vs. + + \| -0.06288 \| -0.5526 to 0.4268 \| No \| ns \| 0.9440 \| A-C \| \| + - vs. + + \| -0.04140 \| -0.5311 to 0.4483 \| No \| ns \| 0.9753 \| B-C \| |
| **Supplementary Figure 6E. IL-18 mRNA level in the Hippocampus of C57BL/6N mice** |
| \|  \|  \|  \|  \|  \|  \|  \| \| --- \| --- \| --- \| --- \| --- \| --- \| --- \| \| Number of families \| 1 \|  \|  \|  \|  \|  \| \| Number of comparisons per family \| 3 \|  \|  \|  \|  \|  \| \| Alpha \| 0.05 \|  \|  \|  \|  \|  \| \|  \|  \|  \|  \|  \|  \|  \| \| Tukey's multiple comparisons test \| Mean Diff. \| 95.00% CI of diff. \| Below threshold? \| Summary \| Adjusted P Value \|  \| \| - - vs. + - \| -0.2465 \| -0.8063 to 0.3133 \| No \| ns \| 0.5187 \| D-E \| \| - - vs. + + \| 0.2251 \| -0.3347 to 0.7849 \| No \| ns \| 0.5767 \| D-F \| \| + - vs. + + \| 0.4716 \| -0.08819 to 1.031 \| No \| ns \| 0.1091 \| E-F \| |
| **Supplementary Figure 6F. HMGB1 mRNA level in the Cortex of C57BL/6N mice** |
| \| Number of families \| 1 \|  \|  \|  \|  \|  \| \| --- \| --- \| --- \| --- \| --- \| --- \| --- \| \| Number of comparisons per family \| 3 \|  \|  \|  \|  \|  \| \| Alpha \| 0.05 \|  \|  \|  \|  \|  \| \|  \|  \|  \|  \|  \|  \|  \| \| Tukey's multiple comparisons test \| Mean Diff. \| 95.00% CI of diff. \| Below threshold? \| Summary \| Adjusted P Value \|  \| \| - - vs. + - \| -0.01580 \| -0.2737 to 0.2421 \| No \| ns \| 0.9869 \| A-B \| \| - - vs. + + \| 0.1498 \| -0.1081 to 0.4077 \| No \| ns \| 0.3279 \| A-C \| \| + - vs. + + \| 0.1656 \| -0.09231 to 0.4235 \| No \| ns \| 0.2602 \| B-C \| |
| **Supplementary Figure 6F. HMGB1 mRNA level in the Hippocampus of C57BL/6N mice** |
| \| Number of families \| 1 \|  \|  \|  \|  \|  \| \| --- \| --- \| --- \| --- \| --- \| --- \| --- \| \| Number of comparisons per family \| 3 \|  \|  \|  \|  \|  \| \| Alpha \| 0.05 \|  \|  \|  \|  \|  \| \|  \|  \|  \|  \|  \|  \|  \| \| Tukey's multiple comparisons test \| Mean Diff. \| 95.00% CI of diff. \| Below threshold? \| Summary \| Adjusted P Value \|  \| \| - - vs. + - \| -0.1147 \| -0.3168 to 0.08741 \| No \| ns \| 0.3439 \| D-E \| \| - - vs. + + \| 0.07160 \| -0.1305 to 0.2737 \| No \| ns \| 0.6506 \| D-F \| \| + - vs. + + \| 0.1863 \| -0.01581 to 0.3884 \| No \| ns \| 0.0743 \| E-F \| |
